# The hematopoietic stem cell MYB enhancer is essential for and recurrently amplified during T cell leukemogenesis

**DOI:** 10.1172/JCI187998

**Published:** 2025-10-23

**Authors:** Carea Mullin, Karena Lin, Elizabeth Choe, Cher Sha, Zeel Shukla, Koral Campbell, Anna C. McCarter, Annie Wang, Jannaldo Nieves-Salva, Sarah Khan, Theresa M. Keeley, Shannon Liang, Qing Wang, Ashley F. Melnick, Pearl Evans, Alexander C. Monovich, Ashwin Iyer, Rohan Kodgule, Yamei Deng, Felipe da Veiga Leprevost, Kelly R. Barnett, Petri Pölönen, Rami Khoriaty, Daniel Savic, David T. Teachey, Charles G. Mullighan, Marcin Cieslik, Alexey I. Nesvizhskii, Linda C. Samuelson, Morgan Jones, Qing Li, Russell J.H. Ryan, Mark Y. Chiang

**Affiliations:** 1 Division of Hematology-Oncology, Department of Internal Medicine, and; 2 Cellular and Molecular Biology Program, University of Michigan School of Medicine, Ann Arbor, Michigan, USA.; 3 Immunology and Microbiology Program, Baylor College of Medicine, Houston, Texas, USA.; 4 Molecular and Cellular Pathology Program, University of Michigan, Ann Arbor, Michigan, USA.; 5 Department of Anesthesiology, Perioperative and Pain Medicine, Stanford University, Stanford, California, USA.; 6 Immunology Program, University of Michigan School of Medicine, Ann Arbor, Michigan, USA.; 7 Department of Molecular and Integrative Physiology, University of Michigan, Ann Arbor, Michigan, USA.; 8 Regeneron Pharmaceuticals, Tarrytown, New York, USA.; 9 Department of Pathology and Immunology, WashU Medicine, St. Louis, Missouri, USA.; 10 Department of Pathology, University of Michigan, Ann Arbor, Michigan, USA.; 11 Department of Pharmacy and Pharmaceutical Sciences, and; 12 Department of Pathology, St. Jude Children’s Research Hospital, Memphis, Tennessee, USA.; 13 Department of Pediatrics, Children’s Hospital of Philadelphia, Philadelphia, Pennsylvania, USA.; 14 Gilbert S. Omenn Department of Computational Medicine and Bioinformatics, University of Michigan, Ann Arbor, Michigan, USA.

**Keywords:** Cell biology, Hematology, Oncology, Hematopoietic stem cells, Leukemias, Mouse models

## Abstract

There is an urgent need to find targeted agents for T cell acute lymphoblastic leukemia (T-ALL). *NOTCH1* is the most frequently mutated oncogene in T-ALL, but clinical trials showed that pan-Notch inhibitors caused dose-limiting toxicities. Thus, we shifted our focus to ETS1, which is one of the transcription factors that most frequently co-bind Notch-occupied regulatory elements in the T-ALL context. To identify the most essential enhancers, we performed a genome-wide CRISPRi screen of the strongest ETS1-dependent regulatory elements. The top-ranked element is located in an intron of *AHI1* that interacts with the MYB promoter and is amplified with *MYB* in approximately 8.5% of patients with T-ALL. Using mouse models, we showed that this enhancer promoted self-renewal of hematopoietic stem cells and T cell leukemogenesis, maintained early T cell precursors, and restrained myeloid expansion with aging. We named this enhancer the hematopoietic stem cell MYB enhancer (H-Me). The H-Me showed limited activity and function in committed T cell progenitors but was accessed during leukemogenesis. In one T-ALL context, ETS1 bound the ETS motif in the H-Me to recruit cBAF to promote chromatin accessibility and activation. ETS1 or cBAF degraders impaired H-Me function. Thus, we identified a targetable stem cell element that was co-opted for T cell transformation.

## Abstract 2

The authors showed that the gene ETS1 turns on a DNA control center reserved for stem cells to give leukemia cells access to cancer-causing functions.

## Graphical abstract

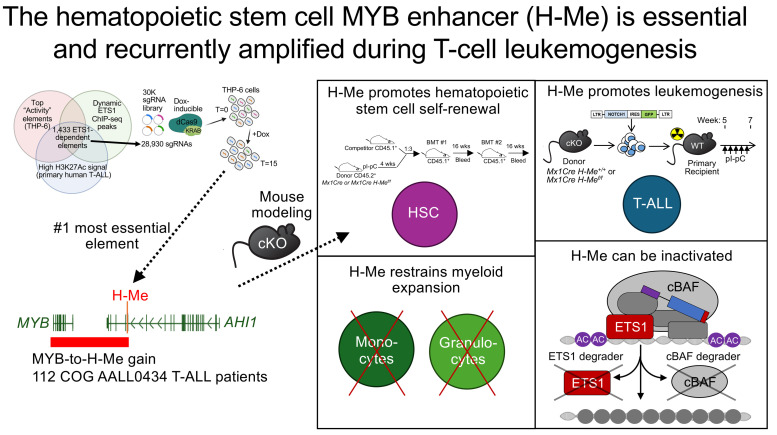

## Introduction

The discovery of Notch-activated tumors, involved in approximately 60% of cases of T-ALL, spurred excitement to clinically test pan-Notch inhibitors, such as γ-secretase inhibitors (GSIs), for the treatment of human cancers (1–3). Unfortunately, early clinical studies reported excessive toxicities with continuous GSI dosing (4–6). GSI toxicities result from abrogation of Notch signals crucial for normal homeostasis, particularly in the intestine (7–9). Proteomic, transcriptional genomic, and biochemical studies show that the core Notch/RBPJ complex can interact with context-dependent transcriptional regulators that co-bind its response elements (10–21). Other regulators also bind near Notch at these elements (15, 22–25). If T-lineage regulators are hijacked to drive Notch-activated T-ALL, then inhibiting them might oppose oncogenic Notch signals while circumventing the toxicities of systemic Notch inhibition. Among these factors, we prioritized ETS1 (26–28). At Notch-bound regulatory elements, we and others showed that ETS is the first- or second-ranked non-RBPJ motif in T-ALL in both frequency and statistical significance (21, 29). Our groups also showed that ETS1 overlaps a strikingly high percentage (76%–82%) of Notch/RBPJ-occupied elements (21, 30), more than any other transcription factor tested. Further, an ETS1 inhibitor is predicted to be safer than GSI given that postnatal *ETS1* expression was highest in the thymus and other lymphoid sites relative to other tissues (31) (Supplemental Figure 1A; supplemental material available online with this article; https://doi.org/10.1172/JCI187998DS1). In contrast, Notch receptor expression showed low tissue specificity (Supplemental Figure 1, B–E). Accordingly, we previously showed that like GSI, Ets1 deprivation in mice suppressed Notch-induced target genes, thymopoiesis, and leukemogenesis but unlike GSI had no significant effects on overall health (21).

For the above reasons, we shifted our focus from Notch to ETS1. Here, we sought to identify the ETS1-dependent network of essential regulatory elements through a genome-wide CRISPR interference (CRISPRi) screen. Our study identified a long-range Notch and ETS1-bound MYB enhancer as the top-ranked essential element; established the important physiological role of this element in hematopoietic stem cells and early T cell progenitors (ETPs); determined the essential oncogenic role of this element for T cell leukemogenesis; showed the dependence of this element on ETS1 for chromatin accessibility and activity in one T-ALL context; and highlighted SWI/SNF and ETS1 degradation but not Notch inhibition as approaches that might target this element, among multiple other effects.

## Results

### Genome-wide CRISPRi essentiality screen of ETS1-dependent regulatory elements nominates the +140 kb MYB enhancer as the most essential element.

**Figure 1:**
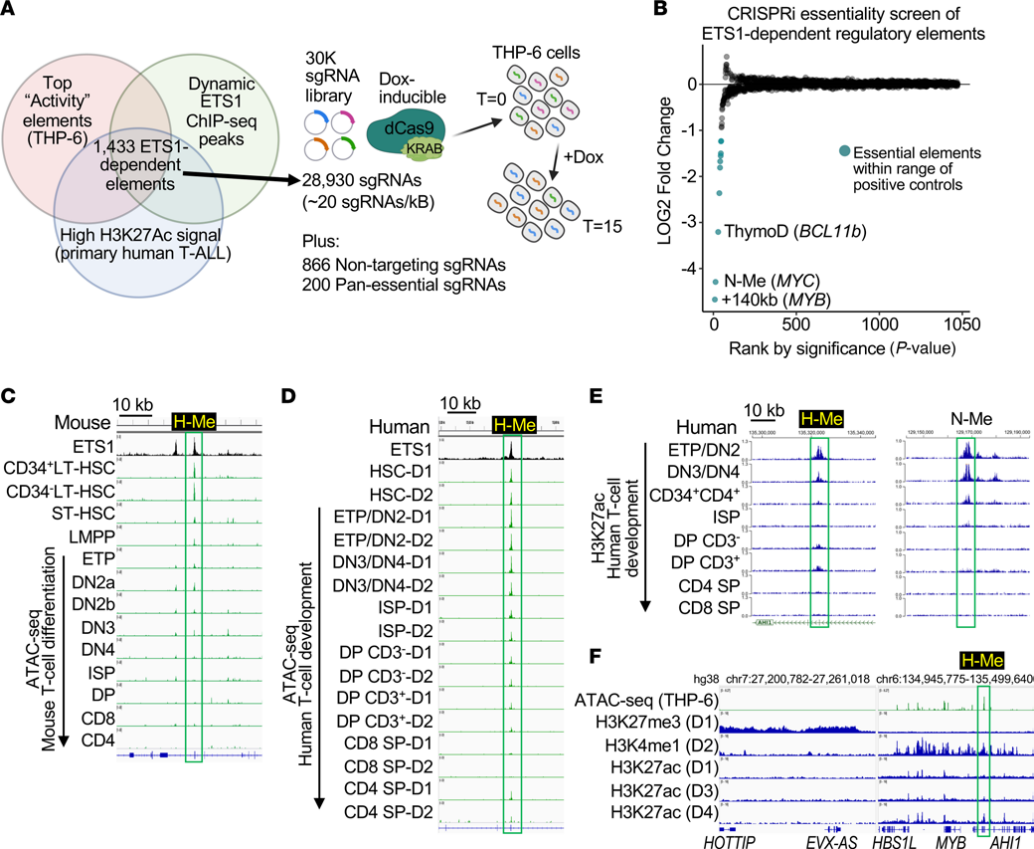
The hematopoietic stem cell MYB enhancer (H-Me) is a top-ranked essential regulatory element in T-ALL cells that appears relatively quiescent in the developmental stages from which T-ALL initiates. (**A**) Schematic of the CRISPRi screen to identify essential ETS1-dependent enhancers in the THP-6 T-ALL cell line. (**B**) CRISPRi essentiality screen results. MAGeCK analysis provided in Supplemental Table 2. (**C**) ATAC-seq profiles of the murine H-Me in LT-HSCs through T cell development (Immgen). (**D**) ATAC-seq profiles of the human H-Me in sorted thymocyte subsets (GSE151075) (105). D1, donor 1; HSC, CD34^+^ cord blood; ETP/DN2, CD34^+^CD4^–^CD1^–^; DN3/DN4, CD34^+^CD4^–^CD1^+^; ISP, CD28^+^CD4^+^CD3^–^CD8^–^; DP, CD4^+^CD8^+^; SP, single positive. (**E**) H3K27ac ChIP-seq profiles of normal thymocyte subsets at the H-Me and the N-Me in 50 kb windows (St. Jude Cloud, https://viz.stjude.cloud/mullighan-lab/collection/the-genomic-basis-of-childhood-t-lineageacute-lymphoblastic-leukemia~29). (**F**) Histone chromatin profiles of DP cells of 4 donors (D1–D4; BLUEPRINT project) comparing a silenced region with the *MYB-AHI1* region.

To assess downstream mechanisms of ETS1, we aimed to characterize the ETS1 “essential regulome” through a high-throughput doxycycline-inducible (DOX-inducible) CRISPRi essentiality screen. CRISPRi is the preferred screening methodology to identify essential regulatory elements (32). We first used our previously published H3K27Ac ChIP-seq and ATAC-seq datasets in THP-6 cells (21) and an activity model (33) to predict highly active T-ALL regulatory elements (Figure 1A). Next, we intersected these elements with “dynamic” ETS1 peaks, which we defined as ETS1 peaks that give decreased H3K27Ac and ETS1 ChIP-seq signal upon *ETS1* knockdown in THP-6 cells (21). Last, we intersected these elements with strong H3K27Ac ChIP-seq peaks in primary T-ALL samples (Blueprint) to identify 1,433 candidate ETS1-dependent regulatory elements. We then transduced THP-6 cells with a custom approximately 30,000 sgRNA library targeting these elements (mean 20 sgRNAs/kb). Model-based Analysis of Genome-wide CRISPR/Cas9 Knockout (MAGeCK) on paired time T = 0 and T = 15 population growth fold doublings (*n* = 3) identified 10 essential elements that were as negatively selected as 39 positive control promoters of pan-essential genes (Figure 1B). The second- and third-ranked elements were the previously reported Notch-dependent *MYC* enhancer (“N-Me”) (34, 35) and the *BCL11B* enhancer (“ThymoD”), respectively (36, 37), thus validating our screen. Whole genome sequencing (WGS) analysis of THP-6 cells revealed a structural variant involving ThymoD and the *NKX2-5* oncogene (Supplemental Figure 2A), similar to previous reports (38–40), that led to *NKX2-5* overexpression (Supplemental Figure 2B). Further WGS and short tandem repeat (STR) analysis showed that THP-6 cells and CEM-CCRF cells were genetically divergent with a common origin. The first-ranked element was an enhancer that lies +140kb from the *MYB* TSS and within intron 23 of *AHI1*. *MYB* is a highly expressed oncogene across all T-ALL subgroups (41). This element was previously identified as a highly conserved enhancer (“MYB-enh-3”) in erythroid leukemia cells that shows correlation between *MYB* expression and chromatin accessibility in human blood cells (42). This enhancer is an ETS1-dependent super-enhancer and makes long-range contacts with *MYB* in T-ALL cells (Supplemental Figure 3, A and B) (21).

### The +140 kb element is a hematopoietic stem cell MYB enhancer that appears relatively quiescent in the developmental stages from which T-ALL initiates.

Publicly available ATAC-seq data in sorted murine cells showed that accessibility of the +140 kb element was high in long-term hematopoietic stem cells (LT-HSCs) but quickly began to fall in short-term HSCs and reduced to baseline at the DN4 stage (Figure 1C). Thus, we presumptively named this element “H-Me” for hematopoietic stem cell MYB enhancer. In human cells, the chromatin was accessible in hematopoietic progenitor cells through DN3/DN4 cells and then went down to baseline at the ISP stage (Figure 1D). However, despite being accessible, H3K27ac profiling showed that enhancer activity dropped considerably at the DN3/DN4 stage (Figure 1E). Histone profiling of human DP thymocytes suggested that the H-Me did not acquire H3K27me3 upon further differentiation (Figure 1F). These data suggest that compared with stem cells, the H-Me was less accessible or active in immature T cells (DN3 to ISP) but was “primed” for reactivation. Thus, T-ALL cells did not necessarily use active enhancers native to their developmental stage during oncogenesis.

### The H-Me restricts HSC numbers and maintains ETPs, but has limited function after T cell commitment.

**Figure 2:**
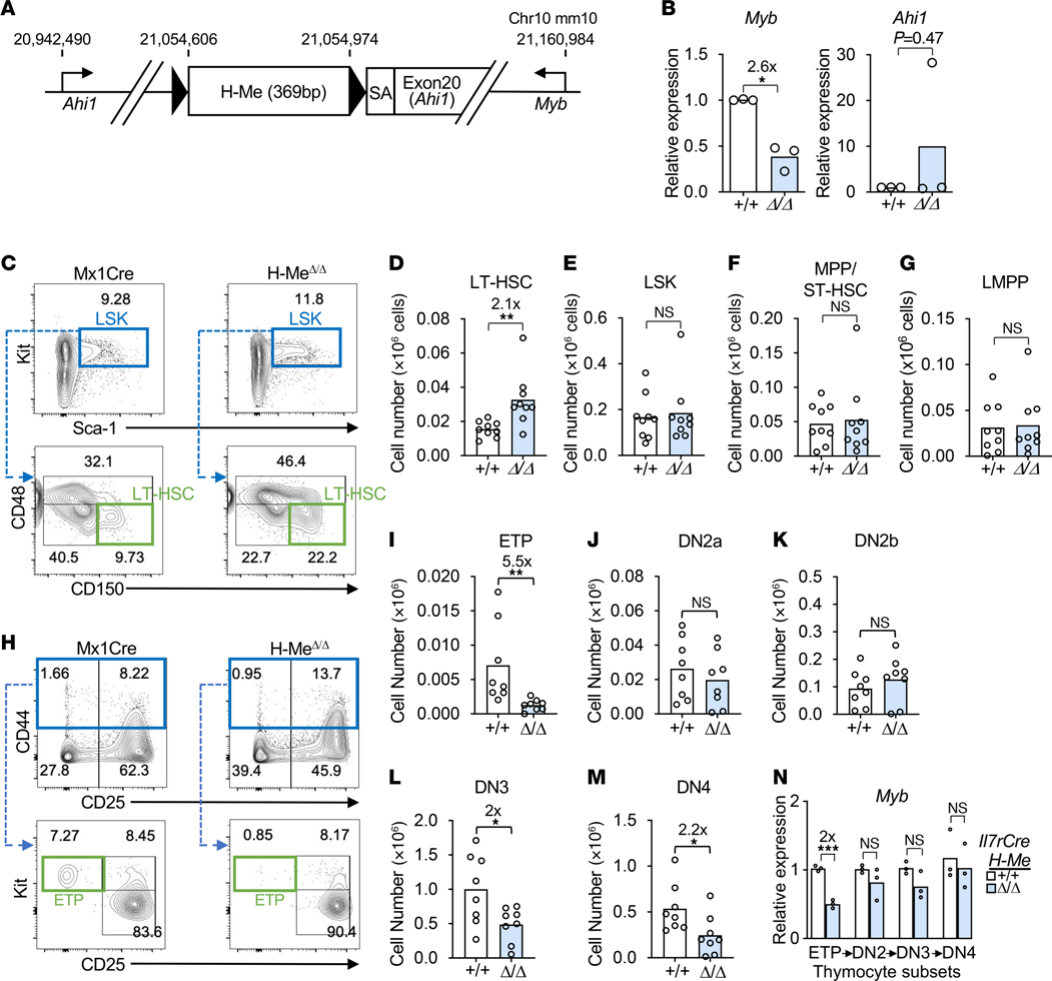
The H-Me normally restricts HSC numbers and maintains ETPs under steady-state conditions. (**A**) Schematic of the floxed H-Me allele (fl). SA, rabbit β-globin splice acceptor sequence (103). (**B**) RT-qPCR of *Myb* and *Ahi1* in sorted LT-HSCs (CD150^+^CD48^–^ LSK cells) from *Mx1Cre H-Me*^Δ*/*Δ^ (Δ/Δ) and littermate control *Mx1Cre* (+/+) mice. (**C**–**G**) Representative BM Lineage^–^ flow cytometry plots (**C**) and absolute numbers of LT-HSCs (**D**), LSK cells (**E**), MPP/ST-HSCs (CD150^+^CD48^–^ LSKs) (**F)**, and LMPPs (Lineage^–^Sca-1^+^Kit^hi^Flt3^hi^) (**G**). (**H**–**M**) Representative thymus Lineage^–^ flow cytometry plots (**H**) and absolute numbers of ETP (Lineage^–^CD44^+^CD25^–^c-Kit^hi^) (**I**) and DN2a (Lineage^–^CD44^+^CD25^+^cKit^hi^) (**J**), DN2b (Lineage^–^CD44^+^CD25^+^c-Kit^lo^) (**K**), DN3 (Lineage^–^CD44^–^CD25^+^) (**L**), and DN4 (Lineage^–^CD44^–^CD25^–^) (**M**) cells. (**N**) *Myb* RT-qPCR in sorted DN cells from *Il7rCre H-Me*^Δ*/*Δ^ (Δ/Δ) and littermate control *Il7rCre* (+/+) mice. Unpaired 2-tailed *t* test. *P < 0.05; **P < 0.01; ***P < 0.001.

To investigate the function of the H-Me, we generated H-Me conditional KO mice (*H-Me^fl/fl^* mice). These mice contain loxP sites flanking the H-Me element, a 369 bp nucleosome-free region homologous to the human H-Me (Figure 2A). We crossed these mice with *Mx1Cre* transgenic mice (43) to generate *Mx1Cre H-Me^fl/fl^* mice and littermate *Mx1Cre* controls. To delete the H-Me in HSCs, we injected 4- to 6-week-old mice with poly(I:C). At 6 weeks, we observed efficient H-Me deletion (Supplemental Figure 4A) and an approximately 61% reduction in *Myb* expression in sorted HSCs (Figure 2B). In contrast, we saw no statistically significant effects on *Ahi1* expression or in hematopoietic stem and progenitor cells (Lineage^–^Sca-1^+^Kit^+^ [LSK] cells; Supplemental Figure 4B). H-Me deletion led to an approximately 2.1-fold increase in long-term HSC numbers (Figure 2, C and D).

In contrast to HSCs, no effects were observed in LSKs (Figure 2E), multipotent progenitor/short-term HSCs (Figure 2F), or lymphoid-primed multipotent progenitor cells (LMPPs; Figure 2G). In the thymus, we observed an approximately 5.5-fold reduction in early T cell precursors (ETPs; Figure 2, H and I); no effects on DN2a (Figure 2J) and DN2b (Figure 2K) cells; an approximately 2-fold reduction in T-committed DN3 (Figure 2L) and DN4 (Figure 2M) cells; and no effect on more differentiated thymocytes (Supplemental Figure 4, C–G) or total thymocytes (Supplemental Figure 4H). H-Me deletion did not affect numbers of BM myeloid/erythroid progenitors; total BM cells; peripheral myeloid and lymphoid cells; or peripheral blood counts (Supplemental Figure 4, I–W). These data suggest that the H-Me has important roles in restricting HSC population growth and in maintaining ETPs. However, there were relatively modest effects, if any, on other cell types.

Next, we crossed *H-Me^fl/fl^* mice with *Il7rCre*-transgenic mice (44), in which the transgene is lymphocyte specific, to generate *Il7rCre H-Me^fl/fl^* mice and *Il7rCre* littermate controls. We observed efficient deletion of the H-Me in the thymus (Supplemental Figure 5A). *Myb* expression decreased ~50% in ETPs but was restored in subsequent stages (Figure 2N). *Ahi1* expression was not affected (Supplemental Figure 5B). Consistent with the effects on *Myb* expression, H-Me deletion reduced ETPs by approximately 3.4-fold (Supplemental Figure 5, C–D) but had no statistically significant effect on subsequent stages (Supplemental Figure 5, E–O). These data suggest that the H-Me has ETP stage–restricted effects in maintaining cell number and *Myb* expression during thymopoiesis.

### Under stress conditions, the H-Me is important for LT-HSC self-renewal but not for primary engraftment.

**Figure 3:**
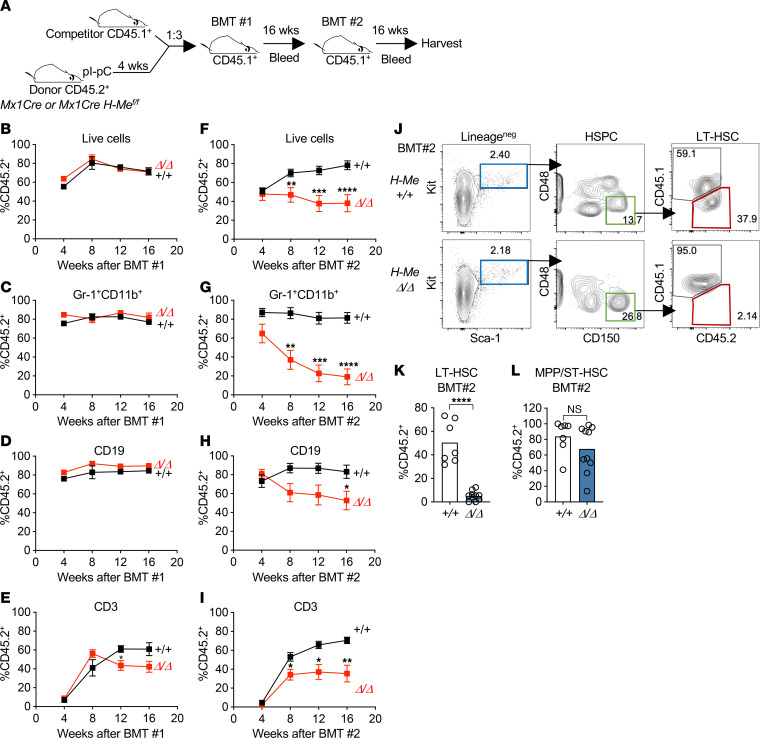
The H-Me is important for long-term HSC self-renewal under stress conditions. (**A**) Schematic of serial competitive BMT experiment. (**B**–**I**) Peripheral blood analysis of indicated subsets tracking percent donor-derived (CD45.2^+^) cells after the first BMT (**B**–**E**) and the second BMT (**F**–**I**). (**J**–**L**) Representative Lineage^–^ flow cytometry plots (**J**) and percent donor-derived (CD45.2^+^) analysis of LT-HSCs (**K**) and MPP/ST-HSCs (**L**) at 16 weeks after the second BMT. Unpaired 2-tailed *t* test. *P < 0.05; **P < 0.01; ***P < 0.001; ****P < 0.0001.

We wondered whether the increased numbers of phenotypically defined HSCs in *Mx1Cre H-Me^Δ/Δ^* mice might herald eventual depletion of LT-HSC capacity as a consequence of non-self-renewing divisions. To test this possibility, we performed serial competitive BM transplants (BMTs; Figure 3A). We observed modest, if any, changes in primary reconstitution of peripheral blood compartments after the first BMT (Figure 3, B–E). However, after the second BMT, H-Me deletion resulted in a significant loss in reconstitution of peripheral blood compartments (Figure 3, F–I). Consistent with these findings, we observed no effect of H-Me deletion on reconstitution of HSCs or MPP compartments after the first BMT (Supplemental Figure 6, A–C) but saw significantly impaired HSC reconstitution after the second BMT (Figure 3, J and K). Total MPP was not affected (Figure 3L). Similarly, in the thymus, *H-Me* deletion modestly reduced reconstitution after the first BMT (Supplemental Figure 6D) but strongly impaired reconstitution after the second transplant (Supplemental Figure 6E). These data suggest that under stress conditions, the H-Me was important for LT-HSC self-renewal but had relatively modest, if any, effects on primary engraftment of stem cell and thymopoietic compartments.

### The floxed H-Me is a hypomorphic enhancer.

The introduction of LoxP sites might have affected expression of the 3 genes in the topologically associating domain (TAD) (*Hbs1l*, *Myb*, and *Ahi1*). To address this, we measured *Hbs1l*, *Myb*, and *Ahi1* expression in HSCs and ETPs from 5- to 8-week-old WT control and *H-Me^fl/fl^* mice. *H-Me^fl/fl^* HSCs showed reduced *Myb* expression compared with controls (Supplemental Figure 7A). *Ahi1* and *Hbs1l* were modestly or not significantly affected (Supplemental Figure 7, B and C). *H-Me^fl/fl^* ETPs showed reduced *Myb* expression compared with controls (Supplemental Figure 7D). *Ahi1* and *Hbs1l* were not significantly affected (Supplemental Figure 7, E and F). We also observed a nonsignificant increase in HSCs (Supplemental Figure 7, G and H) and a decrease in ETPs (Supplemental Figure 7, I and J) in *H-Me^fl/fl^* mice. The effect sizes of the floxed H-Me on *Myb* expression and HSC/ETP numbers were smaller than the effect sizes of H-Me deletion (Figure 2, B, D, and I, and Supplemental Figure 5, B and D). Taken together, these data suggest that the floxed H-Me is a hypomorphic Myb enhancer. The data show a limitation of the mouse model and raise the importance of transplantation experiments for ruling out cell nonautonomous effects.

### The H-Me limits myeloid expansion and HSC loss with aging.

**Figure 4:**
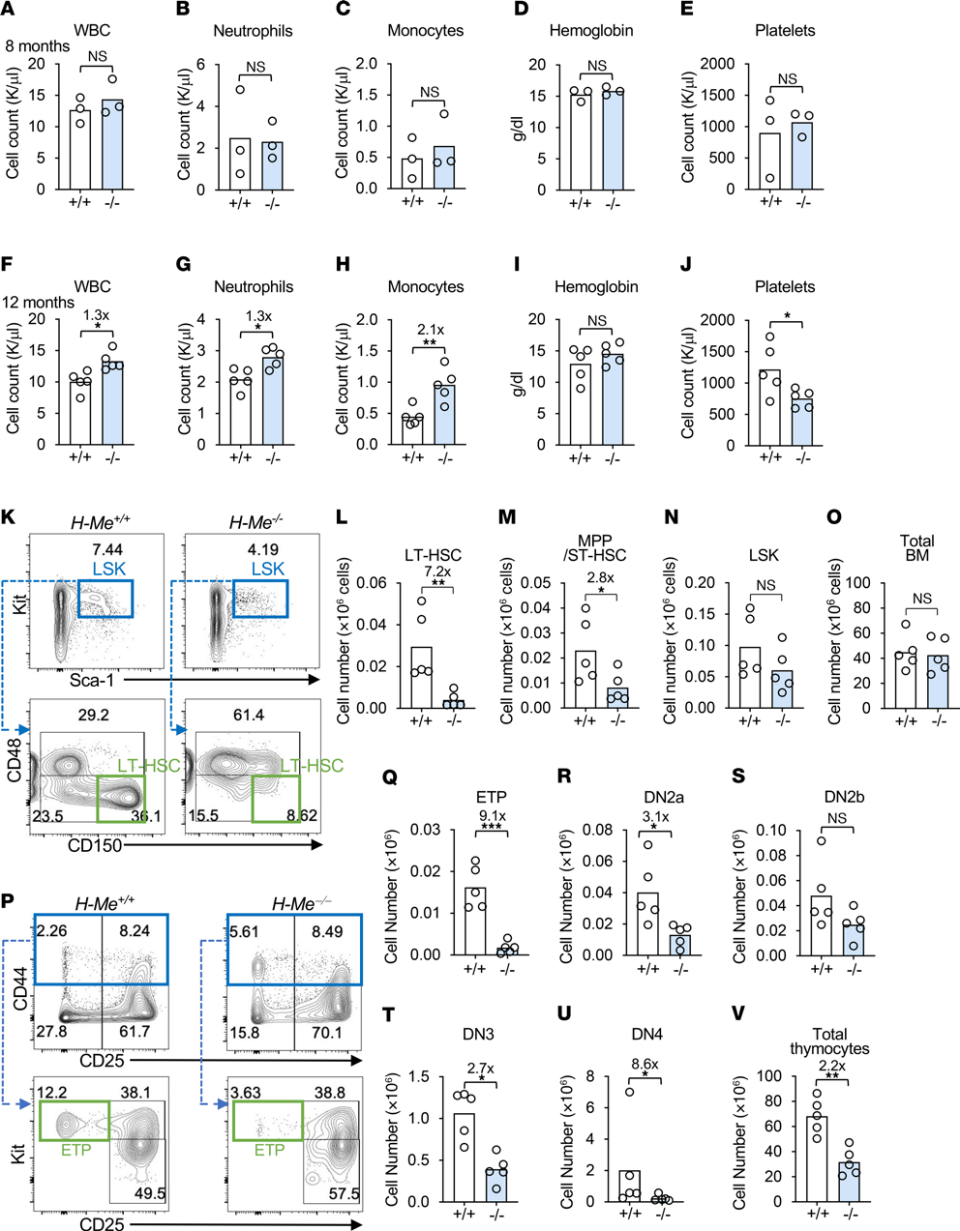
The H-Me limits myeloid expansion and HSC loss with aging. (**A**–**J**) WBC counts (**A** and **F**), neutrophil counts (**B** and **G**), monocyte counts (**C** and **H**), hemoglobin concentrations (**D** and **I**), and platelet counts (**E** and **J**) comparing the indicated mice at 8 (**A**–**E**) and 12 (**F**–**J**) months of age. (**K**–**O**) Representative flow cytometric plots (**K**) and absolute numbers of LT-HSCs (**L**), MPP/ST-HSCs (**M**), hematopoietic stem and progenitor cells (LSK) **N**), and total BM cells (**O**) in 1-year old mice as defined for Figure 2. (**P**–**V**) Representative flow cytometric plots (**P**) and absolute numbers of ETPs (**Q**) and DN2a (**R**), DN2b (**S**), DN3 (**T**), DN4 (**U**), and total thymocyte cells (**V**) as defined for Figure 1. Unpaired 2-tailed *t* test. *P < 0.05; **P < 0.01; ***P < 0.001.

To understand possible toxicities of systemic H-Me inhibition, we compared H3K27ac signals in a variety of tissues in ENCODE (Supplemental Figure 8A). H3K27ac signals at the H-Me were relatively strong in T-ALL cell lines and primary tumors, moderate in erythroid/B cell–derived cells, and undetectable in non-blood cells. Based on these profiles, we predicted that systemic H-Me inactivation would be tolerable and avoid the toxicities of Notch inhibition. To test this, we bred *H-Me^fl/fl^* mice with *Rosa26CreER^T2^* mice. The progeny spontaneously recombined the floxed H-Me and were intercrossed to generate germline *H-Me^–/–^* mice. Next, we observed H-Me–deficient and littermate control mice from birth to 12 months of age. Initially, these mice showed no differences in weight up to 6 months of age. Afterward, H-Me–deficient mice showed 6%–10% reductions in weight that were stable over several months (Supplemental Figure 8B). Importantly, overall survival was not affected (Supplemental Figure 8C). Since our serial transplant studies revealed the importance of the H-Me for LT-HSC self-renewal, we wondered whether H-Me inactivation might cause long-term hematopoietic toxicity. At 8 months of age, peripheral blood analysis showed no differences (Figure 4, A–E). However, at 12 months of age, WBC, neutrophil, and monocyte counts were increased 1.3-fold, 1.3-fold, and 2.1-fold, respectively, while platelet counts were mildly decreased (Figure 4, F–J). In the BM, LT-HSCs were depleted 7.2-fold, while MPP/ST-HSCs were depleted 2.8-fold (Figure 4, K–O). In the thymus, ETPs were severely depleted 9.1-fold (Figure 4, P–Q), while more differentiated populations and total thymocytes decreased by 1.9- to 8.6-fold (Figure 4, R–U) and 2.2-fold (Figure 4V), respectively. These data suggest that the H-Me was important for maintaining HSCs and thymopoiesis and restraining myeloid cell expansion with aging.

### Effects of H-Me deletion on hematopoietic stem and progenitor cells at steady state.

To examine the cell-cycle status of *H-Me^–/–^* as compared with WT hematopoietic stem and progenitor cells at steady state, we injected 10- to13-week-old mice with EdU, then subjected them to a 3-day maintenance period with EdU-containing water. We chose the 10- to 13-week age range in order to reliably analyze adult LT-HSCs at steady state. No difference was observed between groups with respect to EdU uptake in the LSK or LT-HSC populations (Supplemental Figure 9, A and B). Further, Ki-67 staining showed no differences in LSK or LT-HSC cells in the G_0_, G_1_, or S-G_2_-M phases of the cell cycle (Supplemental Figure 9, C and D). These data suggest that cell cycle dynamics were unchanged in adult *H-Me^–/–^* as compared with *H-Me^+/+^* HSPCs at steady state.

To determine whether H-Me deficiency leads to changes in the progenitor cell populations at steady state, we enumerated HSCs and MPPs (45). This staining protocol fractionates MPPs into MPP1 (CD229^–^CD244^–^; with balanced lymphoid and myeloid potential), MPP2 (CD229^+^CD244^–^), MPP3 (CD229^+^CD244^+^; a myeloid-biased subpopulation), and a poorly characterized “MPP other” population (Supplemental Figure 10A) (46). At 10–13 weeks of age, there were no differences in the overall cellularity of BM (Supplemental Figure 10B). However, there was a nonsignificant, 1.9-fold increase in HSCs (Supplemental Figure 10C), which aligned with the HSC expansion seen at an earlier time point (Figure 2D). We did not observe any differences between control and H-Me–knockout mice in the total MPP, LSK, and individual MPP populations (Supplemental Figure 10, D–H). However, we observed a trend toward increased cell number and relative expansion of the myeloid-biased MPP3 population compared with other MPPs (Supplemental Figure 10, H and I). These data are consistent with the eventual myeloid expansion seen in aged H-Me–deficient mice (Figure 4, F–H). The data suggest that at steady state, the HSC expansion seen acutely after H-Me deletion had weakened and there were hints of the myeloid bias seen later in life.

### Effects of H-Me deletion on intestinal morphology.

Given the weight loss of H-Me–deficient mice over time and given the importance of *Myb* and *Ets1* for intestinal homeostasis (21, 47–51), we compared intestinal tissue in the above 10- to 13-week-old H-Me–deficient mice compared with *H-Me^+/+^* control mice. Overall morphology of the intestinal tissue was not changed, as visualized by H&E staining (Supplemental Figure 11A) or PAS/AB staining of mucous cells (Supplemental Figure 11B).

### The H-Me is important for murine Notch-activated T-ALL leukemogenesis.

**Figure 5:**
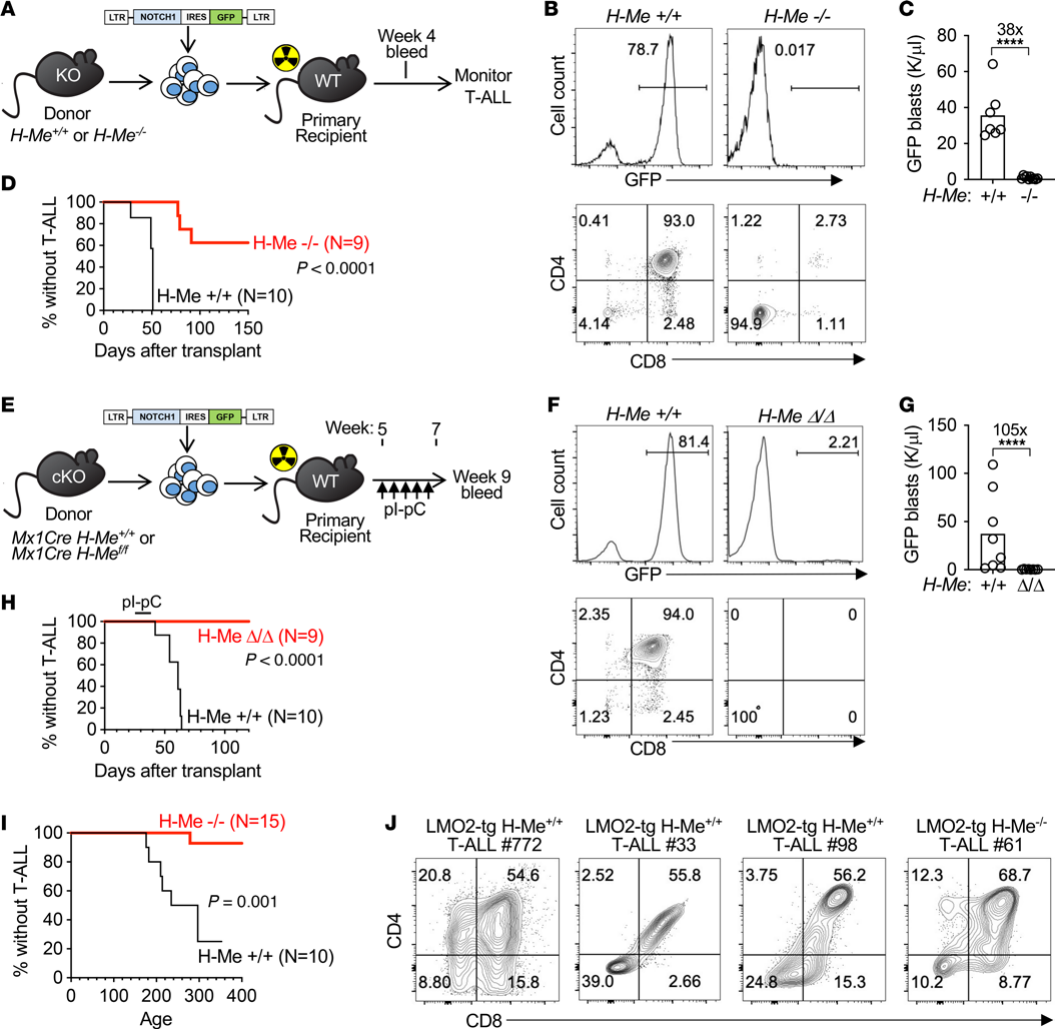
The H-Me is an essential regulatory element for T-ALL leukemogenesis in multiple genetically engineered mouse models. (**A**) Schematic showing generation of Notch-induced T-ALL by transducing *ΔE/Notch1* retrovirus (54, 55) into hematopoietic stem and progenitor cells from *H-Me^–/–^* mice or littermate controls, followed by transplantation. (**B**–**D**) Representative flow cytometry plots (**B**), GFP^+^ blast counts at 4 weeks after transplant (**C**), and leukemia-free survival curves (**D**) for the experiment in **A**. (**E**) Schematic showing generation of Notch-induced T-ALL by transducing *ΔE/Notch1* retrovirus into hematopoietic stem and progenitor cells from *Mx1Cre H-Me^fl/fl^* mice and *Mx1Cre* littermate controls, followed by transplantation and injection of pI-pC to delete the H-Me. (**F**–**H**) Representative flow cytometry plots (**F**), GFP^+^ blast counts at 4 weeks after injection of pI-pC (**G**), and leukemia-free survival curves (**H**) for the experiment in **E**. (**I** and **J**) *Lmo2-tg* *H-Me^–/–^* and littermate control *H-Me^+/+^* mice were observed for survival (**I**) and development of T-ALL, which was confirmed by flow cytometry of thymic mass (mice #33, #98, and #61) or spleen mass (mouse #772) (**J**). Unpaired 2-tailed *t* test and log-rank test. ****P < 0.0001.

Since the H-Me seemed less accessible, active, and functional in early T-committed thymocytes compared with more primitive ancestors, we wondered whether H-Me activity is increased during transformation to drive T-ALL initiation. To test this possibility, we used a well-established murine model of Notch-induced T-ALL (52, 53). We transduced BM stem and progenitor cells from *H-Me^–/–^* mice with an activated *Notch1* allele (*ΔE/Notch1*) (54, 55) and transplanted these cells into recipient mice to generate T-ALL (Figure 5A). H-Me–deleted mice generated approximately 38-fold fewer peripheral T-ALL blasts (Figure 5, B and C) and showed significantly prolonged survival (Figure 5D) relative to littermate control mice.

To further test whether the H-Me is important for T-ALL leukemogenesis, we transduced BM stem and progenitor cells from *Mx1Cre H-Me^fl/fl^* mice with *ΔE/Notch1*, transplanted these cells into recipient mice, and injected pI-pC at week 5 (Figure 5E). H-Me deletion induced an approximately 105-fold reduction in peripheral T-ALL blasts (Figure 5, F and G) and significantly prolonged survival (Figure 5H) relative to *Mx1Cre* littermate control mice. Further, H-Me deletion significantly impaired leukemogenesis in a second Notch-activated T-ALL mouse model (Lmo2-tg) that is induced by an *LMO2* transgene in which activating *Notch1* mutations are acquired (Figure 5, I and J) (56). These data suggest that the H-Me is important for murine Notch-activated T-ALL leukemogenesis.

### The H-Me is frequently coamplified with MYB in T-ALL patients.

**Figure 6:**
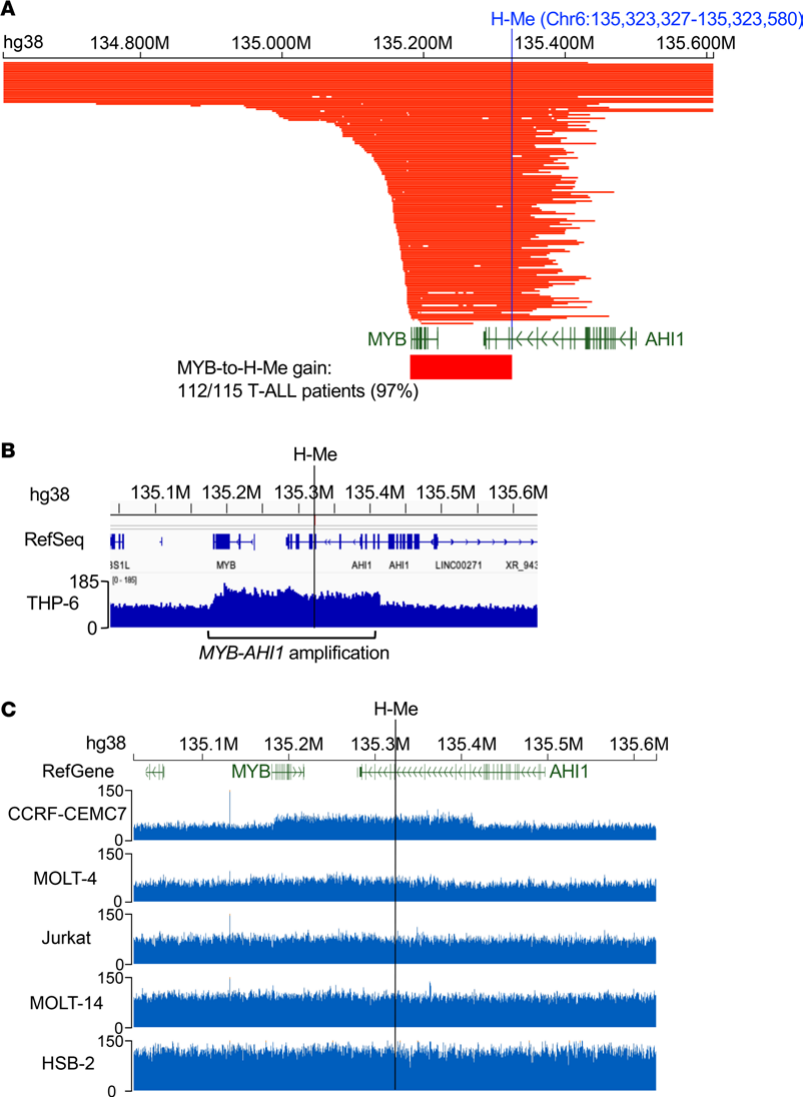
The H-Me is coamplified with *MYB* in nearly all T-ALL patients with *MYB* amplifications. (**A**) Amplified region in primary human T-ALL in the WGS dataset from AALL0434 (*n* = 1,309 patients) (40). Each row represents a unique patient (*n* = 115). Bar shows the *MYB*–to–H-Me region. (**B** and **C**) WGS tracks showing sequence reads in the *MYB-AHI1* region including the H-Me in THP-6 cells (**B**) and other T-ALL cell lines used in this study (**C**).

Previous studies showed that the *MYB-AHI1* region is a frequent target of tandem duplications in approximately 8%–40% of pediatric T-ALL patient samples (57–61). Thus, we wondered whether there was positive selection for including the H-Me in these duplications in human T-ALL patients. Consistently, WGS analysis in 1,309 T-ALL samples from Children’s Oncology Group clinical trial AALL0434 (40) showed that 8.8% of patients had *MYB* amplifications, of which 97% involved the H-Me (Figure 6A). The genomic segment extending from the *MYB* gene to the H-Me appeared to be a minimally amplified region. Further, approximately 50% of cell lines were found to carry *MYB* duplications, such as CEM cells (58). Consistently, WGS analysis showed that THP-6 cells contained MYB/H-ME duplications and thus constituted a clinically relevant model (Figure 6B). WGS confirmed duplication in CEM cells but did not detect it in other T-ALL cell lines used in this study (see below) (Figure 6C). These data support the importance of the H-Me in T-ALL patients and contribute to solving the mystery of why *MYB* duplications nearly always contain part of the *AHI1* gene.

### The H-Me is important for human T-ALL maintenance.

**Figure 7:**
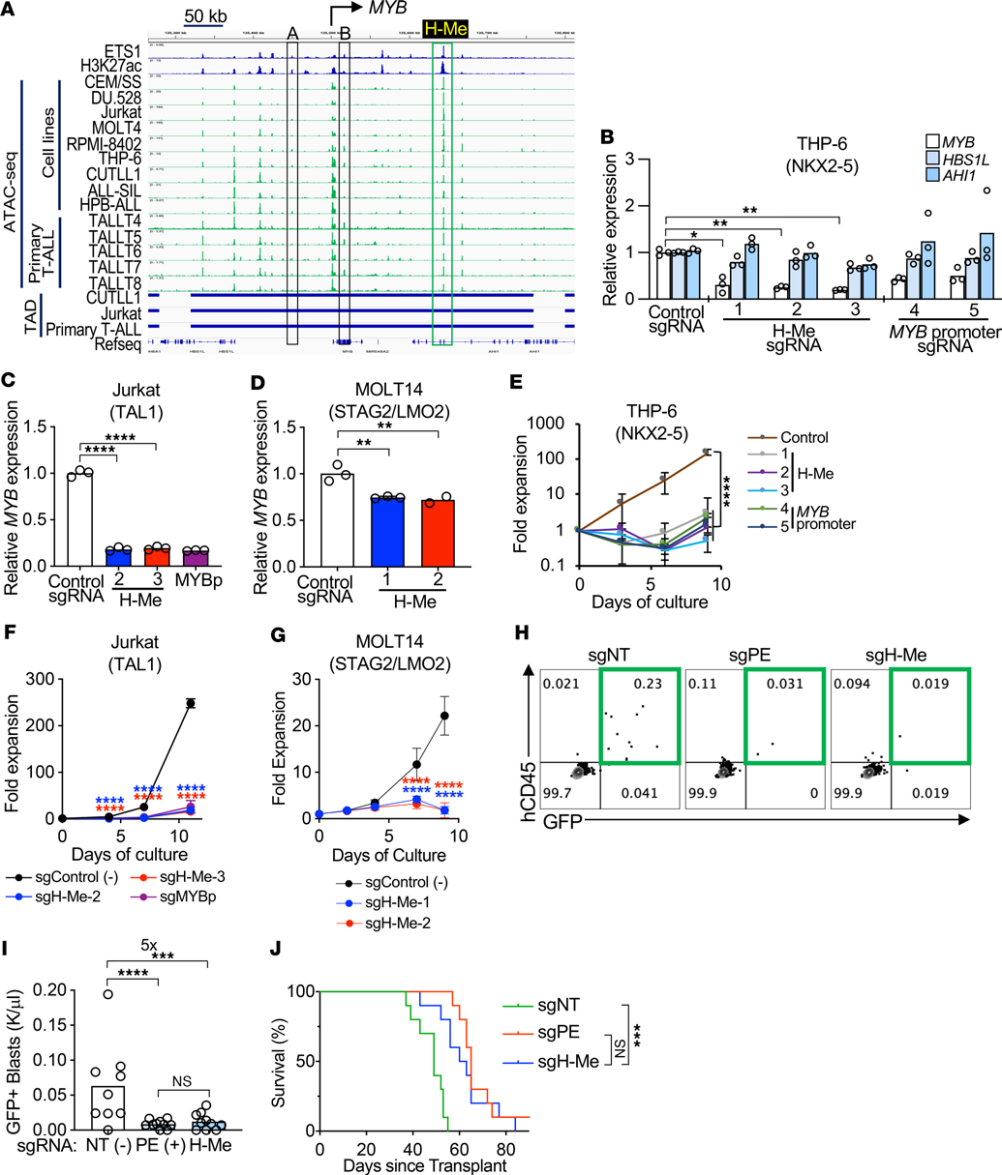
The H-Me is important in diverse models of human T-ALL maintenance. (**A**) ATAC-seq profiles (green) of a panel of T-ALL cell lines and primary samples across the *MYB* TAD. ETS1 and H3K27ac tracks in THP-6 cells are shown in blue (GSE138516). MYB enhancers labeled A and B were previously described (62–64). ATAC-seq datasets from GSE129086, GSE110630, GSE263585, GSE263977, and GSE225559; TAD datasets from GSE134761. (**B**–**G**) THP-6 (**B** and **E**), Jurkat (**C** and **F**), and MOLT14 (**D** and **G**) cells were transduced with constitutive (THP-6) or Dox-inducible (Jurkat, MOLT14) dCas9-KRAB and sgRNAs against the H-Me or the *MYB* promoter and then tested for expression of *MYB* (**B**–**D**) and measured for cell growth (**E**–**G**). *HBS1L* and *AHI1* are flanking genes of *MYB*. (**H**–**J**) CEM cells, related to THP-6, transduced with the indicated sgRNAs were injected into NSG mice and 2–5 days later treated with Dox in drinking water to activate dCas9-KRAB–GFP. Representative GFP/hCD45.2 flow cytometry plots of peripheral blood at 4 weeks after injection (**H**), GFP^+^/hCD45^+^ blast counts (**I**), and survival (log-rank test *P* values) (**J**) were measured. *n* = 9 (control); *n* = 10 (PE); *n* = 10 (H-Me). NT, nontargeting; PE, pan-essential gene *RPL34*. 1-way ANOVA. *P < 0.05; **P < 0.01; ***P < 0.001; ****P < 0.0001.

Within the TAD that contains *MYB*, ATAC-seq of T-ALL cell lines and primary samples showed that the H-Me ranked among the strongest nucleosome-free signal, stronger than previously identified *MYB* enhancers in T-ALL (62–64) (enhancers A and B in Figure 7A). To test the importance of the H-Me, we transduced H-Me sgRNAs and dCas9-KRAB into T-ALL cell lines that represent genomically defined subtypes (40) expressing a range of *MYB* levels in *ETS1*-enriched T-ALL (Supplemental Figure 12). We selected several *NOTCH1*-mutated cell lines — THP-6 (NKX2-5), Jurkat (TAL1) (62), MOLT14 (STAG2/LMO2) (65), CEM (NKX2-5) (39) (related to THP-6), SUP-T1 (TAL1-negative NOTCH1 t[7;9] otherwise not classified) (66, 67), and MOLT4 (TAL1) (68) — as well as one *NOTCH1*-WT cell line, HSB2 (TAL1) (69). The H-Me sgRNAs repressed expression of *MYB* by 1.3-fold to 5.5-fold (Figure 7, B–D, and Supplemental Figure 13, A–D) and impaired cell growth by 1.6-fold to 113-fold (Figure 7, E–G, and Supplemental Figure 13, E–H). MOLT4 cells appeared to be MYB independent, as MYB promoter guides suppressed *MYB* expression but not growth. To rule out non-MYB effects of the H-Me, we ectopically expressed *Myb* and then transduced sgH-Me. Enforced *Myb* expression rescued growth of H-Me repressed cells (Supplemental Figure 13, I and J). To test the importance of the H-Me in vivo, we injected NSG mice with Dox-inducible dCas9-KRAB-expressing CEM cells transduced with sgRNA and then treated the mice with Dox. At 4 weeks, sgH-Me repressed peripheral blood blast count by 5-fold (Figure 7, H and I) and significantly extended survival (Figure 7J). These effects were just as strong as with repressing a pan-essential gene. Taken together, these data show that the H-Me had limited function in normal T-lineage-committed progenitors but rose in importance for malignant transformation, induction of *MYB*, and maintenance of T-ALL cells without compensation by other *MYB* enhancers.

### The ETS motif in the H-Me is primarily bound by ETS1 and is important for H-Me activity in one T-ALL context.

**Figure 8:**
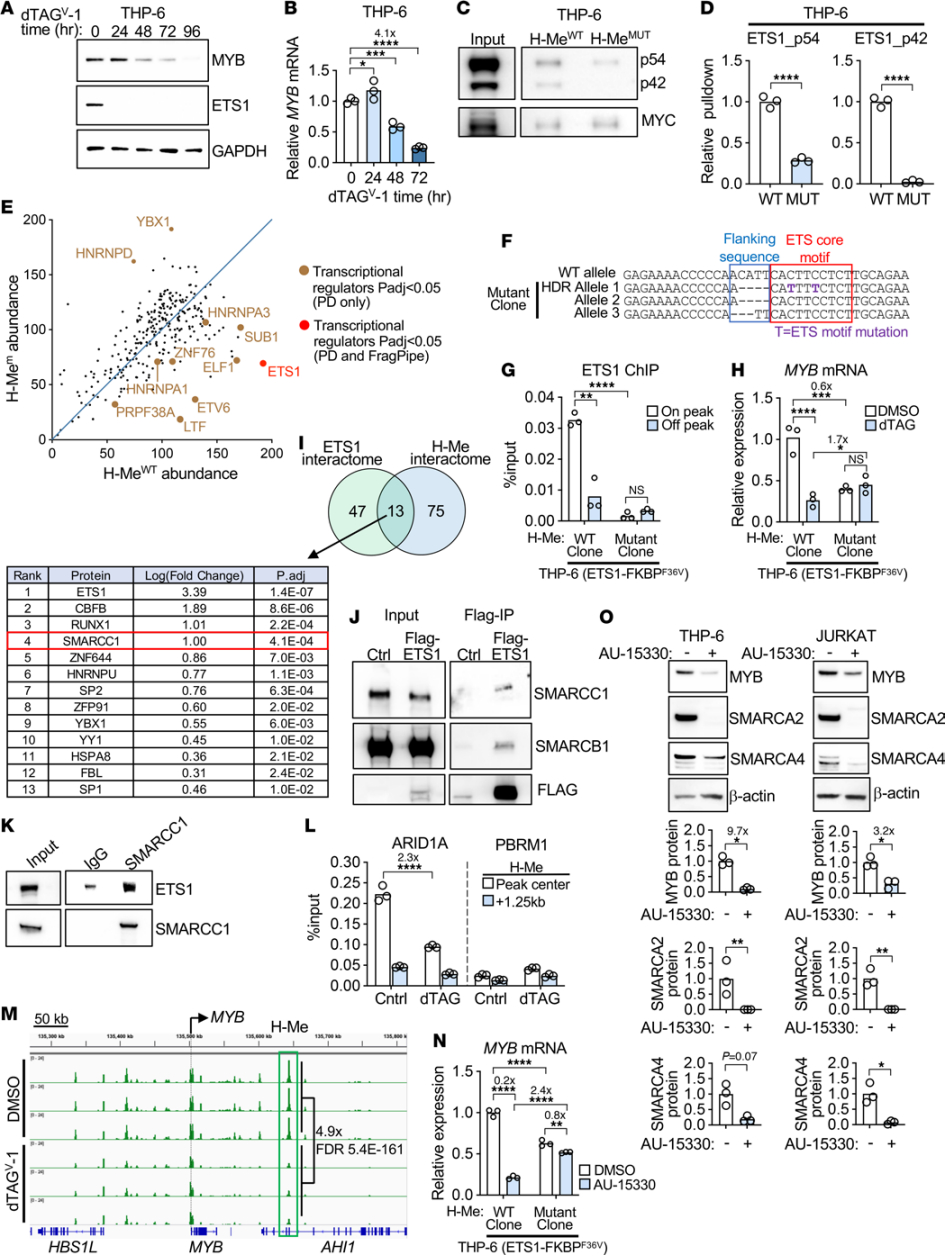
ETS1 recruits cBAF through the ETS motif in the H-Me to activate *MYB* expression in THP-6/CEM cells. (**A** and **B**) Western blot of ETS1 and MYB proteins (**A**) and *MYB* RT-qPCR (**B**) showing the effect of 500 nM dTAG in degrading ETS1 in ETS1-FKBP^F36V^ knockin THP-6 cells. (**C** and **D**) Representative Western blots (**C**) and quantitative ImageJ analyses (**D**) showing effect of the ETS motif mutation on ETS1 binding (p54 and p42 isoforms) in reverse ChIP in THP-6 cells. (**E**) Normalized abundance plot of transcriptional regulators that were pulled down by reverse ChIP and identified by mass spectrometry comparing WT and ETS motif–mutated H-Me; and analyzed with PD (Thermo Fisher Proteome Discoverer) and FragPipe (106–108). Full results are provided in Supplemental Table 3. (**F**–**H**) Sequences of homozygous partially mutated ETS sites in the 3 H-Me alleles (**F**), ETS1 qChIP at the H-Me (**G**); and *MYB* RT-qPCR (**H**) in a subclone of ETS1-FKBP^F36V^–knockin THP-6 cells after CRISPR/Cas9 editing and HDR. (**I**) Venn diagram showing intersection of the H-Me and ETS1 interactomes ranked by strength of interaction with Flag-ETS1. The H-Me interactome was supplemented with transcriptional regulators that met *P_adj_* < 0.1/ LFC (Log2 Fold Change). > 0 criteria by Proteome Discoverer. Full results are provided in Supplemental Tables 4 and 5. (**J**) Flag co-IP assay in vector-transduced (Ctrl) and Flag-ETS1–transduced CEM cells showing interactions with endogenous SMARCC1 and SMARCB1. (**K**) Reciprocal co-IP assay comparing IgG and anti-SMARCC1 pulldowns in CEM cells to detect interactions with endogenous ETS1. (**L**) ARID1A and PBRM1 qChIP using primers at the H-Me peak center or a negative control site 1.25 kb downstream in ETS1-FKBP^F36V^–knockin THP-6 cells treated with 500 nM dTAG to degrade ETS1. (**M**) ATAC-seq profiles of the *MYB* TAD in ETS1-FKBP^F36V^–knockin THP-6 cells treated with dTAG to degrade ETS1. DeSeq2 analysis. (**N**) *MYB* RT-qPCR in ETS1-FKBP^F36V^–knockin THP-6 cells with mutated ETS-binding sites in the H-Me (**F**) treated with DMSO versus AU-15330. (**O**) Western blot for the indicated proteins in DMSO-treated or AU-15330–treated T-ALL cells. 1-way ANOVA. *P < 0.05; **P < 0.01; ***P < 0.001; ****P < 0.0001.

To better understand how ETS1 regulates the H-Me, we first generated THP-6 and CEM (related to THP-6) cell lines in which ETS1 was fused to FKBP^F36V^. Adding dTAG^V^-1 (dTAG) robustly degraded ETS1 protein, resulting in suppression of *MYB* protein and reduction of *MYB* RNA by approximately 4.1-fold in THP-6 cells (Figure 8, A and B) and approximately 2.3-fold in CEM cells (Supplemental Figure 14, A and B). Next, we performed HOMER analysis of the H-Me across several species, which revealed strong conservation of several transcription factor motifs, including a single ETS motif (Supplemental Figure 15). Human-to-mouse conservation at the H-Me was high, at 93% compared with 12%–15% in introns overall (70–72). To test whether ETS1 bound this motif, we performed an assay, termed “reverse ChIP,” which was originally developed by the Pimanda laboratory (73). In this assay, nuclear extracts are incubated with a biotinylated 254bp H-Me DNA fragment containing a WT ETS motif (AGAGGAAGTG) or mutated ETS motif (AGAGAAAATG; full sequence in Supplemental Figure 16A). The mutant DNA fragment pulled down less ETS1 in THP-6, CEM, and Jurkat T-ALL cells compared with WT control (Figure 8, C and D, and Supplemental Figure 16, B–E). Next, we performed mass spectrometry of reverse ChIP pulldowns. As a pulldown assay, this assay could in theory detect closely related ETS factors. However, ETS1 was the only protein that was differentially pulled down by WT compared with mutated DNA fragment by 2 statistical methods (Figure 8E). In a complementary approach, we attempted to generate a mutant THP-6 ETS1-FKBP^F36V^ clone with homozygous deleted ETS binding sites. We observed strong selection against creation of this clone but eventually created one clone with homozygous partial mutations (Figure 8F). ChIP showed undetectable ETS1 occupancy at the mutant H-Me relative to WT control (Figure 8G). These data suggest that ETS1 binds the endogenous ETS site in the H-Me.

Next, we wondered whether the ETS site in the H-Me was functional. To test this, we measured *MYB* mRNA in WT and ETS-mutant cells with or without ETS1 degradation using dTAG. Consistently, *MYB* expression was reduced approximately 60% in mutant compared with WT control cells (Figure 8H). Further, ETS1 degradation reduced *MYB* expression in WT control but had no effect in the mutant clone. The lack of *MYB* responsiveness to ETS1 degradation was not due to clone-specific damage to ETS1-FKBP^F36V^ functionality, as dTAG treatment reduced expression of the ETS1 target genes *LYL1* and *HHEX* (21) (Supplemental Figure 16, F and G). For an orthogonal approach, we developed an H-Me luciferase reporter assay, which showed that the H-Me was active in T-ALL cells but not in U2OS or 293T cells (Supplemental Figure 16H). Mutating the ETS motif (Supplemental Figure 16I) or *ETS1* knockdown (Supplemental Figure 16J) impaired H-Me reporter activity. Although Notch/RBPJ bound the H-Me with γ-secretase dependence (Supplemental Figure 3A) (21), Notch inhibition with GSI did not impair H-Me activity (Supplemental Figure 16K). These data are consistent with multiple public RNA-seq datasets showing that Notch does not generally regulate *MYB* (Supplemental Figure 16L) (30, 74, 75). Thus, Notch appears to defer to ETS1 to induce *MYB* directly through the ETS site in the H-Me in one T-ALL context.

### The SWI/SNF complex is a top-ranked candidate ETS1 cofactor at the H-Me.

Since enhancers are difficult to target, we sought to identify ETS1-bound cofactors at the H-Me. Initially, we examined our H-Me reverse ChIP mass spectrometry data and identified 71 transcriptional regulators that differentially bound WT H-Me bait compared with bead controls (Supplemental Figure 17, A and B), including several DNA-binding sequence-specific transcription factors (Supplemental Figure 17C). Next, we transduced CEM cells with Flag-ETS1 and performed anti-Flag coimmunoprecipitation followed by mass spectrometry. We identified 60 transcriptional regulators that differentially bound Flag-ETS1 compared with vector control (Supplemental Figure 17, D and E), including known partners RUNX1 and its cofactor CBFB (76) (Supplemental Figure 17F). Finally, we intersected the H-Me–interacting proteins with the ETS1-interacting proteins to identify 13 proteins common to both groups and ranked them by strength of binding to ETS1 (Figure 8I). The top 2 proteins were RUNX1 and CBFB. The third-ranked protein was SMARCC1, which is a subunit of SWI/SNF complexes (Supplemental Figure 17C, blue dot). In addition to SMARCC1, 6 other SWI/SNF subunits differentially bound ETS1 (Supplemental Figure 17D, yellow cluster; Supplemental Figure 17F, blue dots). Co-IP confirmed the interaction between Flag-ETS1 and the endogenous SWI/SNF subunits SMARCC1 and SMARCB1 (Figure 8J). Reciprocal co-IP confirmed the interaction between endogenous SMARCC1 and ETS1 (Figure 8K). These data suggest that SWI/SNF complexes might be recruited by ETS1 to activate the H-Me.

### ETS1 recruits the cBAF complex to remodel chromatin at the H-Me in one T-ALL context.

To test whether ETS1 recruits SWI/SNF to the H-Me, we degraded ETS1 in THP-6 cells and performed ChIP for SMARCC1 at the H-Me. Consistently, ETS1 degradation reduced SMARCC1 occupancy by approximately 2.5-fold in THP-6 cells (Supplemental Figure 18A) and approximately 1.6-fold in CEM cells (related to THP-6; Supplemental Figure 18B). ETS1 degradation reduced H3K27ac by approximately 3.2-fold at the H-Me (Supplemental Figure 18C). ETS1 degradation reduced ARID1A signals (specific for cBAF) by approximately 2.3-fold but had no effect on PBRM1 occupancy (specific for PBAF) at the H-Me (Figure 8L). Next, we performed ATAC-seq. ETS1 degradation strongly reduced H-Me chromatin accessibility, by approximately 4.9-fold at an FDR less than 6.8 × 10^–15^ (Figure 8M). Next, we wondered whether the ETS motif in the H-Me is required for SWI/SNF function. To test this, we measured *MYB* mRNA in the ETS-mutant cells (Figure 8F) after SWI/SNF degradation with AU-15330. Consistently, SWI/SNF degradation reduced *MYB* expression in ETS-mutant cells by only approximately 20%, in contrast to approximately 80% in control cells (Figure 8N). The lack of *MYB* responsiveness to SWI/SNF degradation was not due to clone-specific damage as AU-15330 treatment strongly repressed the ETS1 target genes *LYL1* and *HHEX* (21) in this clone (Supplemental Figure 18, D and E). These data suggest that ETS1 recruits the cBAF complex to the H-Me to promote chromatin accessibility and *MYB* induction in one T-ALL context.

### cBAF inhibitors inactivate the H-Me and downregulate MYB in 2 T-ALL contexts.

The above data raised the possibility that proteolysis-targeting chimera (PROTAC) degraders of SMARCA2/4, such as ACBI1 (77) and AU-15330 (78), might be effective in targeting the H-Me. To test this, we generated dose-response curves with these compounds on CEM, Jurkat, and THP-6 cells. All 3 cell lines showed less than 100 nM sensitivity to AU-15330, which classifies them as “sensitive” (Supplemental Figure 19A) (78). Jurkat and THP-6 cells, but not CEM cells, were also sensitive to ACBI1 (Supplemental Figure 19B). To test whether SMARCA2/4 degradation inactivates the H-Me, we performed H3K27ac qChIP. AU-15330 treatment reduced H3K27ac signals by approximately 11.1-fold in THP-6 cells (Supplemental Figure 19C) and approximately 2.1-fold in CEM cells (Supplemental Figure 19D). Consistently, AU-15330 suppressed *MYB* transcripts by approximately 1.3- to 4-fold (Supplemental Figure 19, E–G) and MYB protein levels by approximately 2.3- to 9.7-fold (Figure 8O and Supplemental Figure 20A). ACBI1 failed to degrade SMARCA2/4 protein levels in CEM cells (Supplemental Figure 20B), which is consistent with its weak growth inhibitory effects and lack of effect on *MYB* transcripts (Supplemental Figure 20C) and protein (Supplemental Figure 20B) in these cells. In contrast, ACBI1 suppressed *MYB* transcripts by approximately 1.4- and 2-fold and MYB protein by approximately 7 and 25-fold in Jurkat and THP-6 cells, respectively. These data suggest that SMARCA2/4 degradation might be an effective strategy for inhibiting H-Me activity and *MYB* expression, among multiple other effects (79), in 2 T-ALL contexts.

## Discussion

While many T-ALL–associated transcriptional regulators have been identified, there remains limited understanding of the noncoding elements at which these factors assemble and coordinate in hierarchies to promote oncogenesis. Despite important work in this area (22, 62, 63, 80–82), there are knowledge gaps, particularly in identifying the most important oncogenic enhancers and finding ways to safely eject transcription factors bound to these elements as potential therapeutic strategies. To help close these knowledge gaps, we performed an unbiased essentiality screen of regulatory elements that bind ETS1 and are dependent on ETS1 for H3K27ac modification in the Notch-activated THP-6 cell line. Our screen showed that the most essential ETS1-dependent element was the H-Me, a Notch-independent superenhancer that induces *MYB*. In T-ALL, supraphysiological MYB activity can be induced through translocations, tandem duplications, and genetic variants conferring increased protein stability; however, most T-ALL tumors (~83%–95%) have no known *MYB* genetic lesion (57–63, 83). Thus, native elements such as the H-Me are likely important drivers of MYB expression in most cases, including tandem duplications, since they nearly always amplify the H-Me. Our screen also showed that the second most essential element was the Notch-dependent MYC enhancer (N-Me) (34, 35).

Although the H-Me and the N-Me share similarities as developmental enhancers that are co-opted to promote T-ALL oncogenesis, they confer different stage-specific dependences. The marked T cell developmental defects in *LckCre Myc^Δ/Δ^* mice phenocopy the marked defects in N-Me–deficient mice (34). In contrast, the marked T cell developmental defects in *LckCre Myb^Δ/Δ^* mice (84) are not seen in H-Me–deficient mice. These phenotypic differences might be explained by the disparate chromatin configurations of these enhancers in DN3/pre-T cells when *LckCre* initially becomes active (85). While N-Me accessibility peaks at DN3/DN4 (22), H-Me accessibility peaks developmentally earlier in primitive HSCs. While the N-Me showed high H3K27ac signals in DN3/DN4 cells, the H-Me showed low H3K27ac signals (Figure 1E). Hence, it is not surprising that N-Me deletion caused marked T cell developmental defects, whereas H-Me deletion did not. We and others previously showed that DN3, DN4, or ISP cells are highly enriched for leukemia-initiating cells, depending on the T-ALL mouse model (86–88) and similar to human T-ALL (89). Thus, the N-Me appears to be fully accessible to immature T cells as they transform to T-ALL. In contrast, it is not apparent how developing T-ALL cells can easily access the oncogenic potential of the H-Me. These cells presumably restructure the H-Me to a more open configuration or aberrantly sustain the stem cell configuration during T cell differentiation in order to hijack this stem cell element to drive oncogenesis. Emerging evidence suggests that developmental T-ALL enhancers can be separated into thymocyte-active elements (e.g., *Myc* and *Pten*) and stem cell–active elements (e.g., *Myb* and *Mycn*) (34, 35, 90, 91).

*Myb* deletion and H-Me deletion show similarities and differences. Conditional *Myb* deletion using *Mx1Cre* led to dramatic reductions in HSCs, MPPs, myeloid progenitors, and differentiated blood populations (92). In contrast, conditional H-Me deletion using *Mx1Cre* acutely led to an increase in HSCs and no statistically significant effects on MPPs, myeloid progenitors, or differentiated blood populations. Later, between intermediate and aged time points, the H-Me–deficient HSC expansion waned and then reversed to HSC depletion with evolving relative and/or absolute myeloid expansion, particularly monocytosis. The H-Me–deficient HSC and monocyte phenotypes are similar to what has been reported comparing 2-month-old and 12-month-old *Myb^+/–^* mice (93). Induction of *Myb* haploinsufficiency leads to time-dependent accumulation of myeloid proliferation disease by 22 months of age (93). Germline *Myb* deletion is embryonic lethal due to severe anemia (94), while germline H-Me–deleted mice have normal survival with normal blood counts and weights up until 6–8 months. A single competitive BMT of conditional *Myb*-deleted or *Myb^+/–^* BM revealed impaired HSC self-renewal or reconstitution (92, 93). However, conditional H-Me–deficient mice required serial competitive BMTs to reveal impaired HSC self-renewal or reconstitution. *Myb* deletion using *LckCre* led to and approximately 10-fold loss in thymus cellularity, particularly at the DP thymocyte stage (84). In contrast, conditional H-Me deletion using *Mx1Cre* or *Il7rCre* caused stage-restricted ETP loss during thymopoiesis. Taken together, the results indicate that H-Me deletion has milder and/or stage-restricted effects compared with *Myb* deletion. While these data highlight an example of a theoretical safety advantage of enhancer targeting over gene targeting, they also show that enhancer targeting would still have important ramifications, particularly in old age.

We are mindful that the H-Me is a single enhancer within a large transcriptional hub (Supplemental Figure 3A). Thus, it is possible that deletion of the H-Me or insertion of the LoxP sites might affect other Myb regulatory elements in the TAD. Insertion of the LoxP sites also creates a hypomorphic H-Me enhancer. This is an important limitation of our floxed mouse model, although the conclusions of this study are based on comparisons between *H-Me^+/+^* and *H-Me^Δ/Δ^* mice as well as orthogonal models, such as germline H-Me–knockout mice and human T-ALL cell lines.

The literature is replete with examples of Notch signaling being central to T-ALL oncogenesis (3). However, we here highlight a contrasting example of a top oncogenic element where Notch is not dominant, instead deferring to ETS1. The reason why NOTCH1 binds the H-Me is unclear. It is possible that NOTCH1 induced H-Me activity early during leukemogenesis, but subsequent compensatory Notch-independent signals later converged on the H-Me, rendering Notch occupancy dispensable. Consistently, HSB2 cells, which lack *NOTCH1* mutations, require Notch-independent signals at the H-Me for population cell growth. Mechanistically, ETS1 appears particularly important, since it binds and recruits the cBAF complex to the ETS site to promote accessibility and induce *MYB* in THP-6/CEM cells. Since open chromatin is required for transcription factor binding, the chromatin remodeling function of ETS1 likely explains our previous observations that *ETS1* knockdown reduced Notch and Notch cofactor occupancy at a subset of ETS1-bound enhancers (21) (Supplemental Figure 20D).

The importance of SWI/SNF and ETS1 occupancy at the H-Me might have therapeutic implications. Consistently, SMARCA2/4 and ETS1 degradation inactivated the H-Me, resulting in downregulation of *MYB*. The resultant growth-inhibitory effects are consistent with recent reports that cBAF is important for promoting chromatin accessibility and transcriptional activity of RUNX1/CBFB, which were the top ETS1 interaction partners in our screen (Figure 8I) (79, 95). While these findings are promising, there are some limitations. First, we caution that the details of how ETS1 activates the H-Me and *MYB* have not been tested in all T-ALL contexts. Second, our H-Me ETS motif–knockout reporter assay showed expected loss of gene activation; and while reporter assays are statistically predictive of true regulatory activity in high-throughput assays (96–100), effects of individual reporter constructs may not always recapitulate all regulatory effects in situ. While we were able to generate a THP-6 clone with homozygous H-Me mutations that led to loss of ETS1 binding and regulatory effects on *MYB*, we were unable to derive clones with homozygous loss of the core ETS motif, possibly due to strong negative fitness effects. Third, since transcriptional regulators such as ETS1 and cBAF are not specific to *MYB* or the H-Me but regulate thousands of target genes, inhibiting them might not be safe in humans (21, 79). Fourth, our study does not offer a unique mechanism or strategy to target a single gene out of thousands of genes or a single enhancer out of thousands of enhancers. Despite these limitations, given the safety of ubiquitous *Ets1* deletion in mice (21), and since SWI/SNF inhibitors are entering clinical trials (101), our study suggests that ETS1 and cBAF degradation are reasonable therapeutic strategies to test in human T-ALL.

## Methods

Further information is provided in Supplemental Methods.

### Sex as a biological variable.

For developmental and gene expression analyses, mice of both sexes generated from heterozygous-to-heterozygous matings were used to generate data. The data were combined when no differences were noted. Since no differences were noted, sex was not considered as a biological variable for all experiments.

### Mice.

C57/BL6N mice between 4 and 8 weeks of age were purchased from Taconic. 564 B6-Ly5.1/Cr mice between 4 and 8 weeks were purchased from Charles River Laboratories. Mice were backcrossed to the C57/BL6N strain at least 5 times. *Mx1Cre* mice were a gift from Qing Li (University of Michigan). Il7rCre mice were a gift of H.R. Rodewald (German Cancer Research Center) (44). NSG mice were obtained from The Jackson Laboratory (strain 005557). The conditional H-Me floxed mice were generated using Easi-CRISPR (102) by the University of Michigan Transgenic Animal Model Core. Briefly, ES cells were microinjected with a megamer donor containing 5′ and 3′ loxP sites flanked the 369 bp H-Me segment (chr10:21,054,606-21,054,974; mm10), Cas9, and 5′ and 3′ sgRNA guides. A 47-bp rabbit β-globin splice acceptor sequence (103) was placed in the megamer donor between the 3′ loxP site and Exon 20 of *Ahi1*. The *Mx1Cre H-Me^fl/fl^* and *Il7rCre H-Me^fl/fl^* mice were generated by crossing *H-Me^fl/fl^* mice with Mx1-Cre mice and Il7rCre mice, respectively. Cre expression in *Mx1Cre H-Me^fl/fl^* mice that were 4–6 weeks of age was induced with pI-pC (Amersham; 40 μg i.p. every 2 days, 5 times) and analyzed at 6 weeks after the last injection (11–13 weeks of age). *H-Me^fl/fl^* mice of both sexes were analyzed at 5–8 weeks of age. *Il7Cre H-Me^fl/fl^* mice of both sexes were analyzed at 5–10 weeks of age. Germline H-Me knockout mice of both sexes were analyzed at 10–13 weeks of age. Aged H-Me–deficient mice of both sexes were analyzed at 52–54 weeks of age.

### Constructs.

Flag-ETS1 construct was generated as previously described (1). H-Me^WT^ and H-Me^Mut^ constructs were generated by subcloning from designed gBlocks from IDT into the pGL3-luciferase-promoter vector (Promega). The TET3G activator plasmid was generated by subcloning the TET3G from pLVX-EF1a-Tet3G (Clontech 631359) into pRRLsin.cPPTCTS.MNDU3.BXE.PGK.NGFR.WPRE (gift from A. Weng, British Columbia Cancer Agency, Vancouver, British Columbia, Canada). CRISPRi sgRNAs were cloned into the sgOPTI virus (Addgene 85681; RRID:Addgene_85681) and cotransduced with TRE-KRAB-dCas9-IRES-GFP virus (Addgene 85556; RRID:Addgene_85556) and TET3G activator virus (104). These constructs were used for all CRISPRi experiments with the exception of CEM and THP-6 cells in Figure 3, I and J, which used a constitutive CRISPRi construct, pLV-hU6-sgRNA-hUbC-dCAS9-KRAB-T2a_puro (Addgene 71236; RRID:Addgene_71236).

### Cell lines.

CEM/SS, THP-6, SUP-T1, MOLT4, and Jurkat cells were obtained and cultured as previously published (20, 21). HSB2 and MOLT14 cells were cultured by our research team. DOX-inducible CRISPRi cell lines were generated as previously published (104). All human cell lines were authenticated using STR analysis prior to use to match an established cell line standard (DSMZ) or an internal standard when a reference was not available (THP-6) (Labcorp). All cell lines were cultured less than 3 months after resuscitation and tested for contaminants using MycoAlert (Lonza) every 1–3 months to ensure they were free of *Mycoplasma* contamination.

### Reagents.

Antibodies, sgRNAs, homology directed repair (HDR) templates, and primers are listed in Supplemental Table 1.

### Statistics.

Unless otherwise indicated, *P* values were derived from 2-sided 2-sample *t* tests of log_2_-transformed data for comparisons in experiments involving 2 groups and 1-way ANOVA for pairwise contrasts in experiments with more than 2 groups. Unless otherwise stated, horizontal lines are means and values are shown as mean ± SD. Survival curves (or time to event data) were tested with log-rank tests comparing pairs of groups. A *P* value less than 0.05 was considered significant.

### Data availability.

High-throughput sequencing data, results, and statistics were deposited in the NCBI’s Gene Expression Omnibus (GEO GSE263585, GSE263913, GSE263952, and GSE263977) and are publicly accessible. The publicly available next-generation sequencing (NGS) datasets used during the present study can be found in GEO under accession numbers GSE225559, GSE221345, GSE151075, GSE51800, GSE29600, GSE134761, GSE94000, GSE29181, GSE79422, GSE117749, GSE93755, GSE138516, GSE109125, GSE22601, GSE129086, GSE110630, GSE76783, GSE90715, GSE116873, and GSE138659. H3K27ac ChIP-seq profiles of sorted thymocyte subsets were obtained from the St. Jude Cloud, “The genomic basis of childhood T-lineage acute lymphoblastic leukemia” (https://viz.stjude.cloud/mullighan-lab/collection/the-genomic-basis-of-childhood-t-lineageacute-lymphoblastic-leukemia~29). BLUEPRINT project DP thymocyte histone profiles were obtained from EGAD00001002369. WGS datasets were obtained from phs002276.v2.p1, phs000218, phs000464. Supporting data values are provided in Supplemental Tables 2–5, including those for all data points in graphs (Supplemental Table 5).

## Author contributions

CM and KL are co-first authors. CM appears first in the author byline as they began work on this study prior to KL. MYC, CM, KL, RJHR, AC Monovich, PP, DTT, CGM, and MC conceptualized the study. CM, KL, CS, AC McCarter, EC, AFM, SL, QW, AI, KRB, MJ, KC, TMK, R Kogdule, PE, AW, JNS, SK, and ZS performed investigations. MYC, RJHR, CM, KL, EC, KC, MJ, TMK, and PP visualized the data. MYC, CM, KL, CS, EC, YD, FVL, KBR, and PP performed formal analysis. MYC, CM, KL, and DS curated the data. MYC and CGM acquired funding. MYC, CM, KL, EC, KC, MJ, and LCS wrote the original draft of the manuscript. CM, KL, CS, AC Monovich, EC, AFM, SL, QW, AI, AC McCarter, DTT, CGM, PP, MC, QL, MJ, LCS, YD, DS, FVL, AIN, and RJHR reviewed and edited the manuscript. MYC, AIN, R Khoriaty, DS, DTT, CGM, and RJHR supervised the study.

## Funding support

This work is the result of NIH funding, in whole or in part, and is subject to the NIH Public Access Policy. Through acceptance of this federal funding, the NIH has been given a right to make the work publicly available in PubMed Central.

- NIH, R01CA276117 (to MYC), R01AI136941 (to MYC), T32GM145470 (to KL), F30CA288021 (to KL), P30CA021765 (to CGM).
- University of Michigan Rackham Graduate School.
- Michigan Medicine Rogel Cancer Center.
- American Lebanese Syrian Associated Charities of St. Jude Children’s Research Hospital.
- Rally Foundation for Childhood Cancer Research.

## Supplementary Material

Supplemental data

Unedited blot and gel images

Supplemental table 1

Supplemental table 2

Supplemental table 3

Supplemental table 4

Supplemental table 5

Supplemental table 6

Supporting data values

## Version 1 10/23/2025

In-Press Preview

## Version 2 01/02/2026

Electronic publication
