# Supplementary material for: The hematopoietic stem cell MYB enhancer is essential for and recurrently amplified during T cell leukemogenesis
Source: J Clin Invest. 2025 Oct 23;136(1):e187998. doi: 10.1172/JCI187998 (PMC12721900; doi:10.1172/JCI187998)
Supplement: Supplemental data [file jci-136-187998-s155.pdf]

Fig. S1

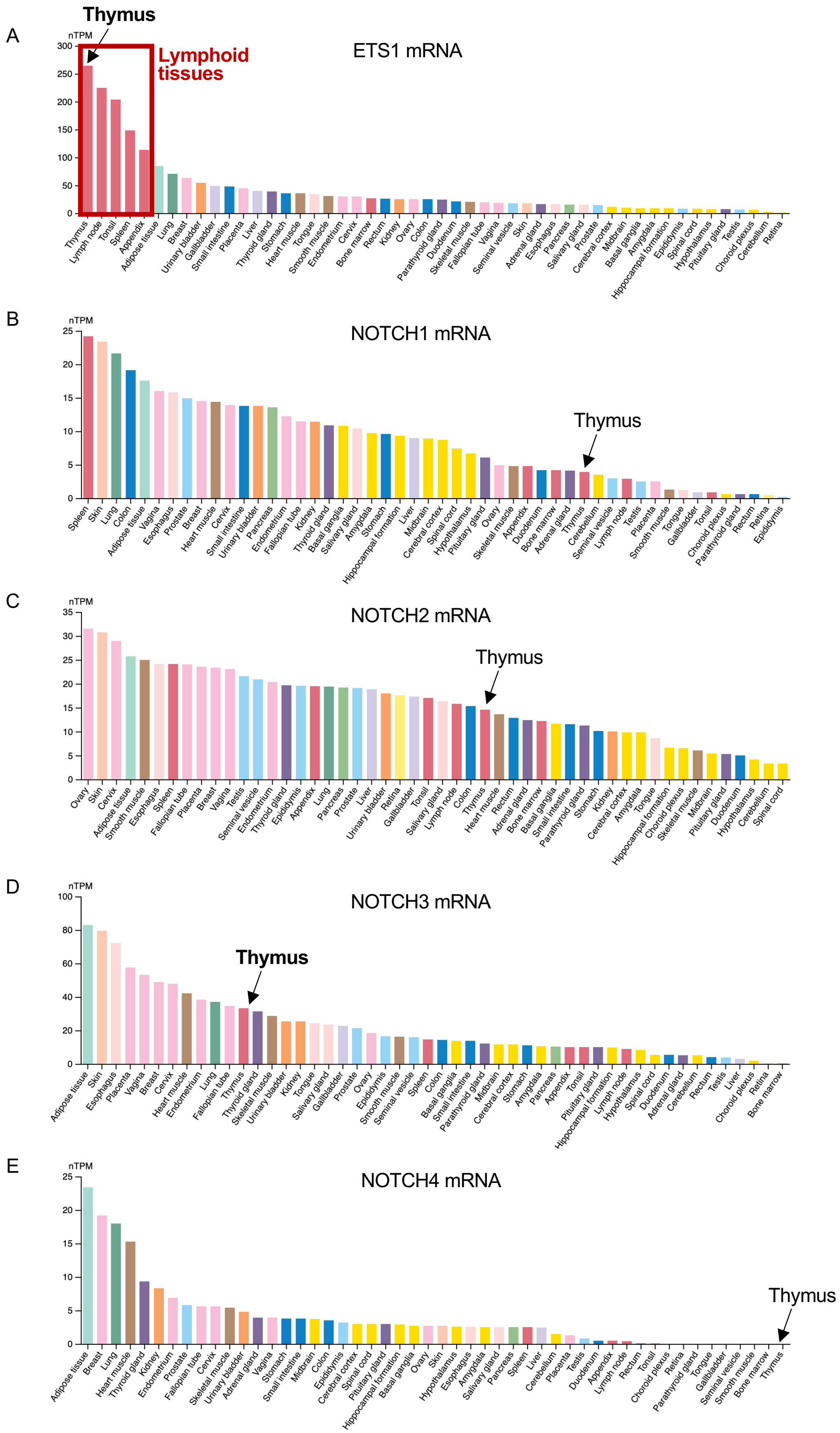

Figure S1. *ETS1* expression is highest in thymus and other lymphoid tissues. mRNA expression (nTPM) in the Human Protein Atlas Consensus RNA dataset for ETS1 (A), *NOTCH1* (B), *NOTCH2* (C), *NOTCH3* (D), *NOTCH4* (E). https://www.proteinatlas.org/ (downloaded 5/28/25).

A

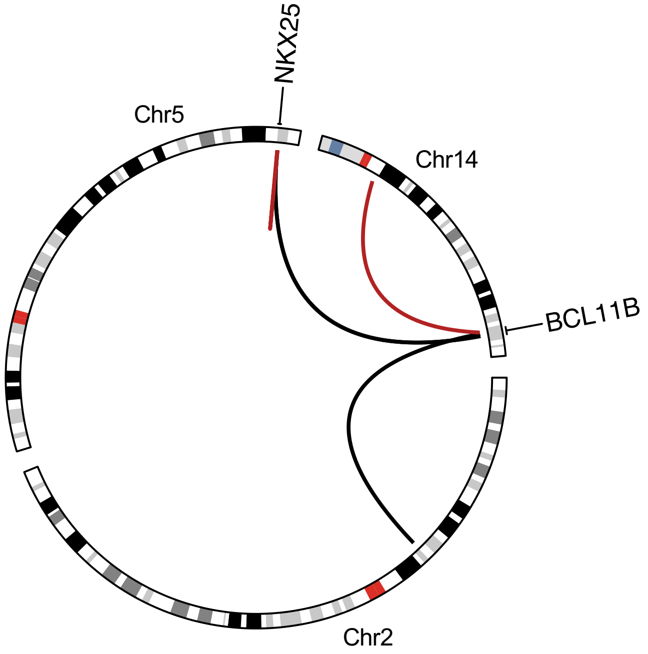

B

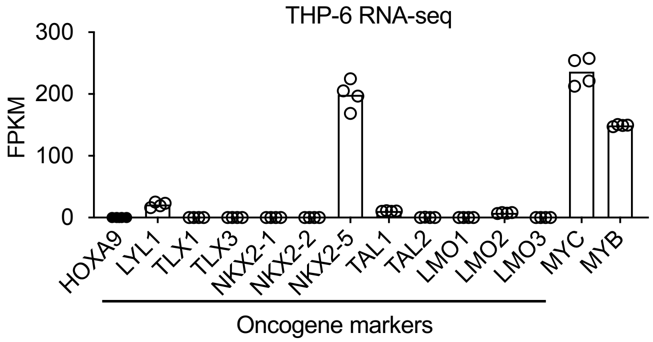

Figure S2. THP-6 cells have a structural variant involving the BCL11B enhancer (ThymoD) and the *NKX2-5* oncogene that activates *NKX2-5* expression. A) Circos plot showing translocations (black) and duplications (red) involving chromosomes 2, 5, and 14. B) THP-6 RNA-seq data (GSE138659) showing expression of *MYC, MYB,* and oncogene markers that help define T-ALL subtype.

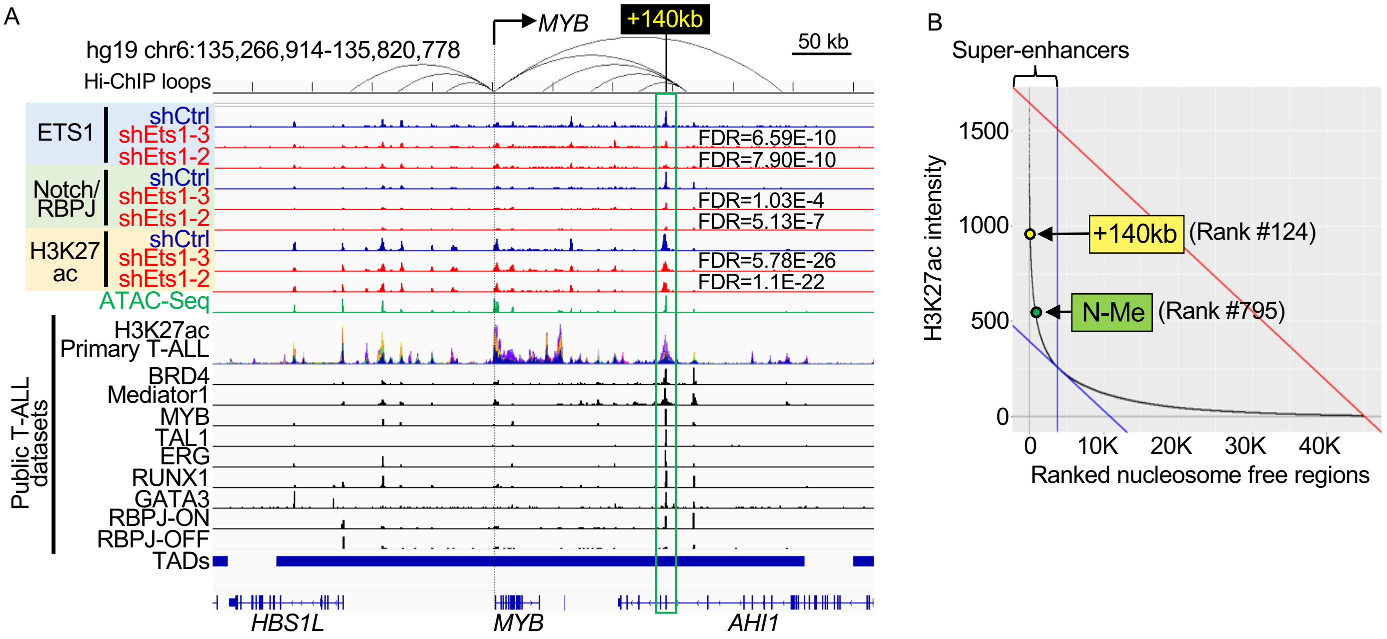

Figure S3. The +140kb MYB enhancer is an ETS1-dependent super-enhancer in T-ALL cells. A) ChIP-seq datasets in the MYB TAD from previous reports (GSE138516 (1) and GSE221345 (2)) comparing control shRNA and shETS1-transduced THP-6 cells were reanalyzed with DESeq2 analysis in the context of nucleosome-free regions (GSE225559). Public datasets were Blueprint H3K27ac (N=8), GSE51800, GSE29600, GSE134761, GSE29181, GSE94000. RBPJ OFF=GSI treatment, which abrogates RBPJ binding. B) Super-enhancer analysis showing ranking of the +140kb enhancer and the Notch-MYC enhancer (N-Me) (3, 4).

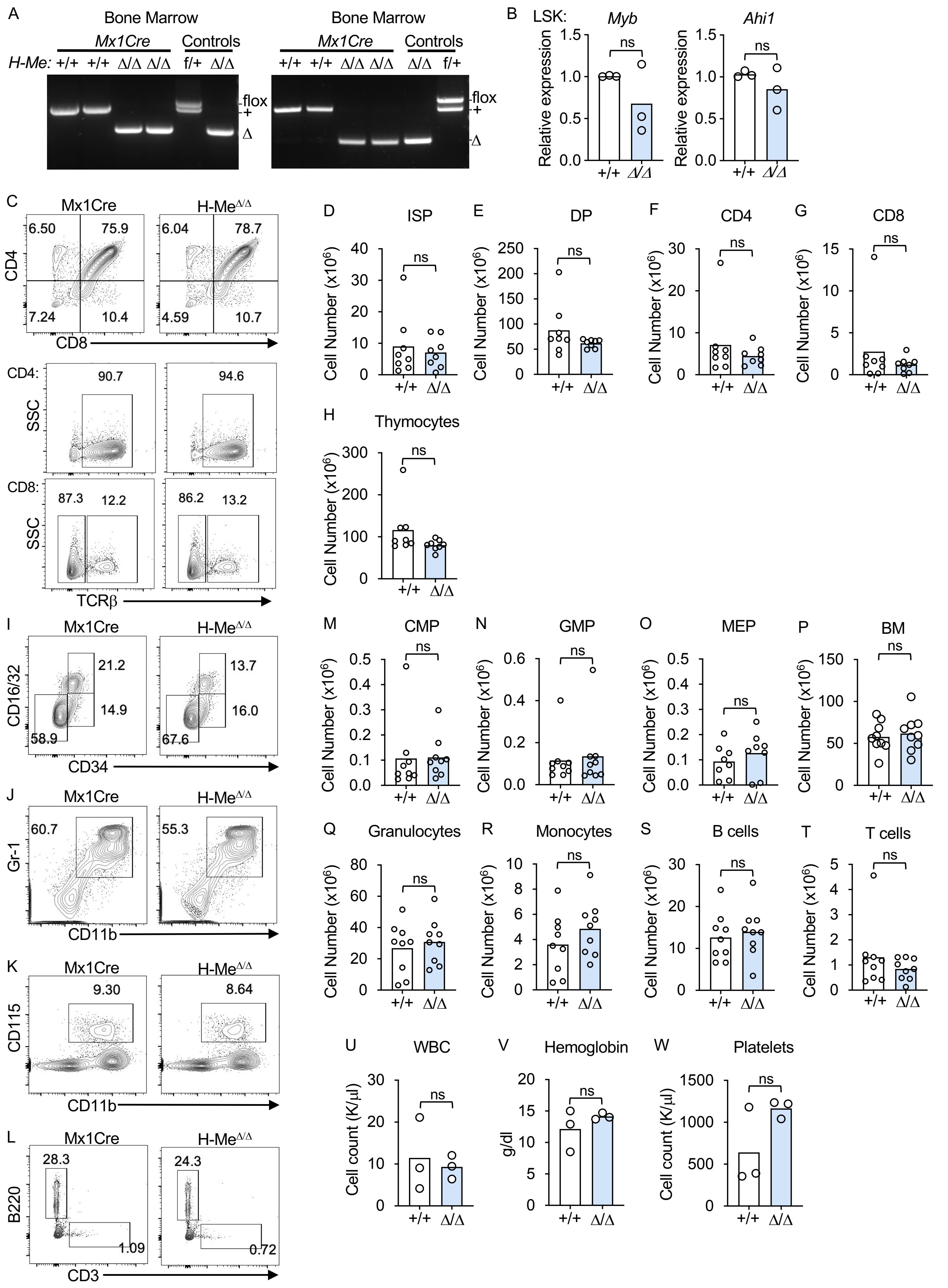
A
Bone Marrow
Mx1Cre
Controls
H-Me: +/+ +/+ Δ/Δ Δ/Δ f/+ Δ/Δ
+/+ +/+ Δ/Δ Δ/Δ Δ/Δ f/+
flox
+
Δ
B
LSK:
Myb
Ahi1
Relative expression
ns
+/+ Δ/Δ
C
Mx1Cre
H-Me$^{Δ/Δ}$
CD4
CD8
6.50 75.9 7.24 10.4
6.04 78.7 4.59 10.7
CD4:
90.7
94.6
SSC
CD8:
87.3 12.2
86.2 13.2
TCRβ
D ISP
E DP
F CD4
G CD8
H Thymocytes
Cell Number (x10^6)
I
CD16/32
CD34
21.2 14.9 58.9
13.7 16.0 67.6
M CMP
N GMP
O MEP
P BM
J
Gr-1
CD11b
60.7
55.3
K
CD115
9.30
8.64
L
B220
CD3
28.3 1.09
24.3 0.72
Q Granulocytes
R Monocytes
S B cells
T T cells
U WBC
V Hemoglobin
W Platelets
Cell count (K/μl)
g/dl

Figure S4. The hematopoietic stem cell MYB enhancer (H-Me) is functionally unimportant in post-DN immature T cells and non-T lineages. A) PCR analysis for H-Me deletion in *Mx1Cre H-Me*$^{\Delta/\Delta}$ mice (Δ/Δ) at 6 weeks after pI-pC injection. B) *Myb* and *Ahi1* qRT-PCR analysis in sorted LSK cells. C-H) Representative flow cytometry plots (C) and absolute numbers of ISP (D, $CD4^{-}CD8^{+}TCR\beta^{-}$), DP (E, $CD4^{+}CD8^{+}$), CD4 (F, $CD4^{+}CD8^{-}TCR\beta^{+}$), CD8 (G, $CD4^{-}CD8^{+}TCR\beta^{+}$) and total thymocytes (H) of *Mx1Cre H-Me*$^{\Delta/\Delta}$ (Δ/Δ) and littermate *Mx1Cre* control (+/+) mice. I-T) Representative flow cytometry plots (I-L) and absolute numbers of CMPs (M, $Lineage^{-}Kit^{+}Sca\text{-}1^{-}CD16/32^{lo}CD34^{+}$), GMPs (N, $Lineage^{-}Kit^{+}Sca\text{-}1^{-}CD16/32^{hi}CD34^{+}$), MEPs (O, $Lineage^{-}Kit^{+}Sca\text{-}1^{-}CD16/32^{-}CD34^{-}$), total bone marrow cells (P, BM), splenic granulocytes (Q, $Gr\text{-}1^{+}CD11b^{+}$), splenic monocytes (R, $CD11b^{+}CD115^{+}$), splenic B cells (S, $B220^{+}CD3^{-}$), and splenic T cells (T, $B220^{+}CD3^{-}$) comparing indicated mice. U-W) Peripheral blood white blood counts (WBC, U), hemoglobin concentrations (V), and platelet counts (W) comparing indicated mice.

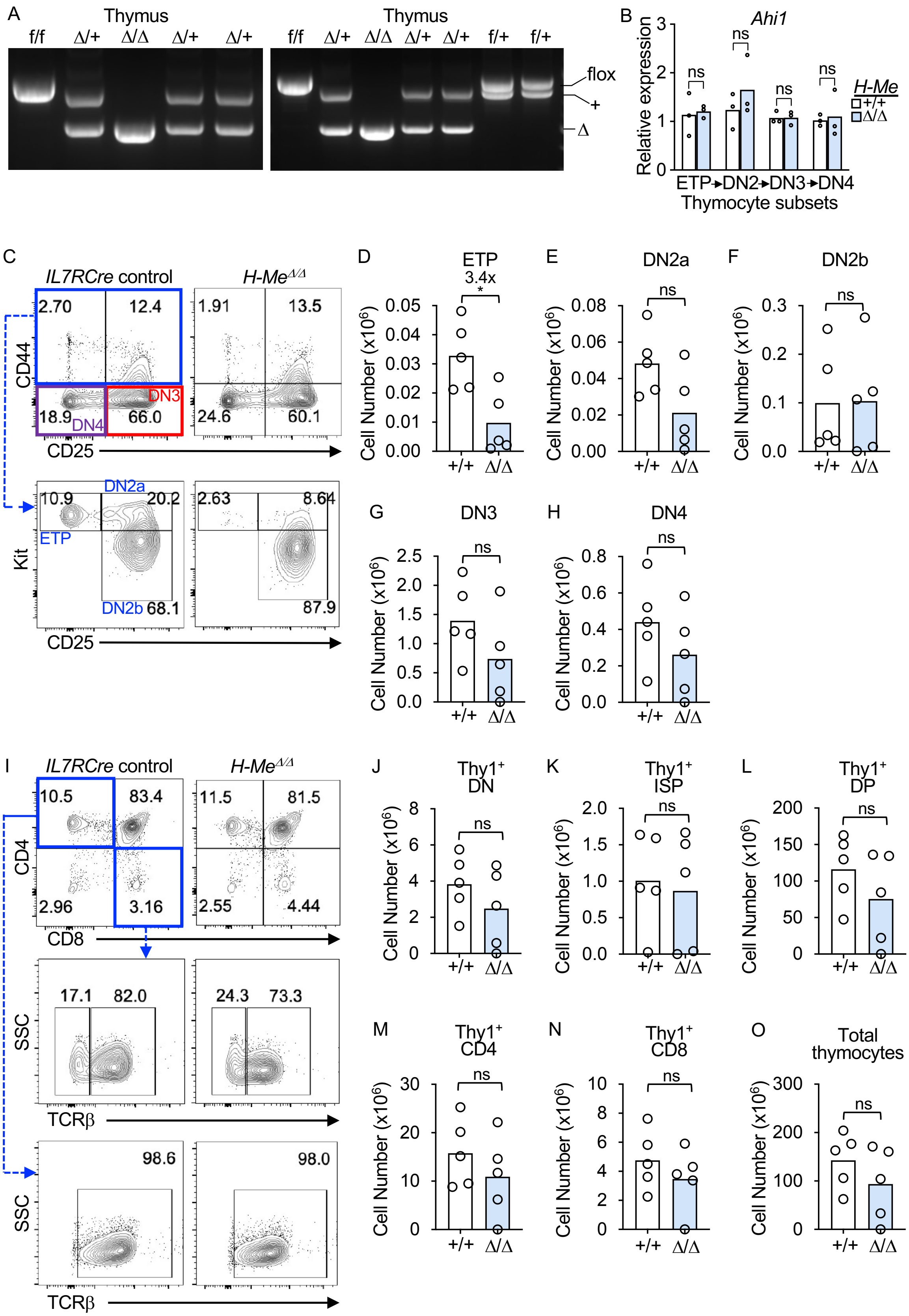

A
Thymus
f/f Δ/+ Δ/Δ Δ/+ Δ/+
Thymus
f/f Δ/+ Δ/Δ Δ/+ Δ/+ f/+ f/+
flox
+
Δ
B
Ahi1
Relative expression
ns
ETP DN2 DN3 DN4
Thymocyte subsets
H-Me
+/+
Δ/Δ
C
IL7RCre control
H-Me$^{\Delta/\Delta}$
2.70 12.4
18.9 DN4 66.0 DN3
1.91 13.5
24.6 60.1
CD44
CD25
DN2a
10.9 20.2
ETP
DN2b 68.1
2.63 8.64
87.9
Kit
CD25
D
ETP
3.4x
Cell Number (x10^6)
+/+ Δ/Δ
E
DN2a
F
DN2b
G
DN3
H
DN4
I
IL7RCre control
H-Me$^{\Delta/\Delta}$
10.5 83.4
2.96 3.16
11.5 81.5
2.55 4.44
CD4
CD8
17.1 82.0
24.3 73.3
SSC
TCRβ
98.6
98.0
J
Thy1$^+$ DN
K
Thy1$^+$ ISP
L
Thy1$^+$ DP
M
Thy1$^+$ CD4
N
Thy1$^+$ CD8
O
Total thymocytes

Figure S5. The H-Me maintains ETPs but has so significant function during subsequent stages of T-cell development. A) PCR analysis for H-Me deletion in *Il7rCre H-Me*$^{\Delta/\Delta}$ (Δ/Δ) and littermate *Il7rCre* control (+/+) mice. B) *Ahi1* qRT-PCR in sorted thymocyte subsets in indicated mice. C-H) Representative Lineage^{-} flow cytometry plots (C) and absolute numbers of ETP (D), DN2a (E), DN2b (F), DN3 (G), and DN4 (H) comparing indicated mice. I-O) Representative flow cytometry plots (I) and absolute numbers of Thy1^{+} DN cells (J), ISP (K), DP (L), CD4 (M), CD8 (N), and total thymocytes (O) as defined in Fig. S4 comparing indicated mice. *P<0.05.

Fig. S6

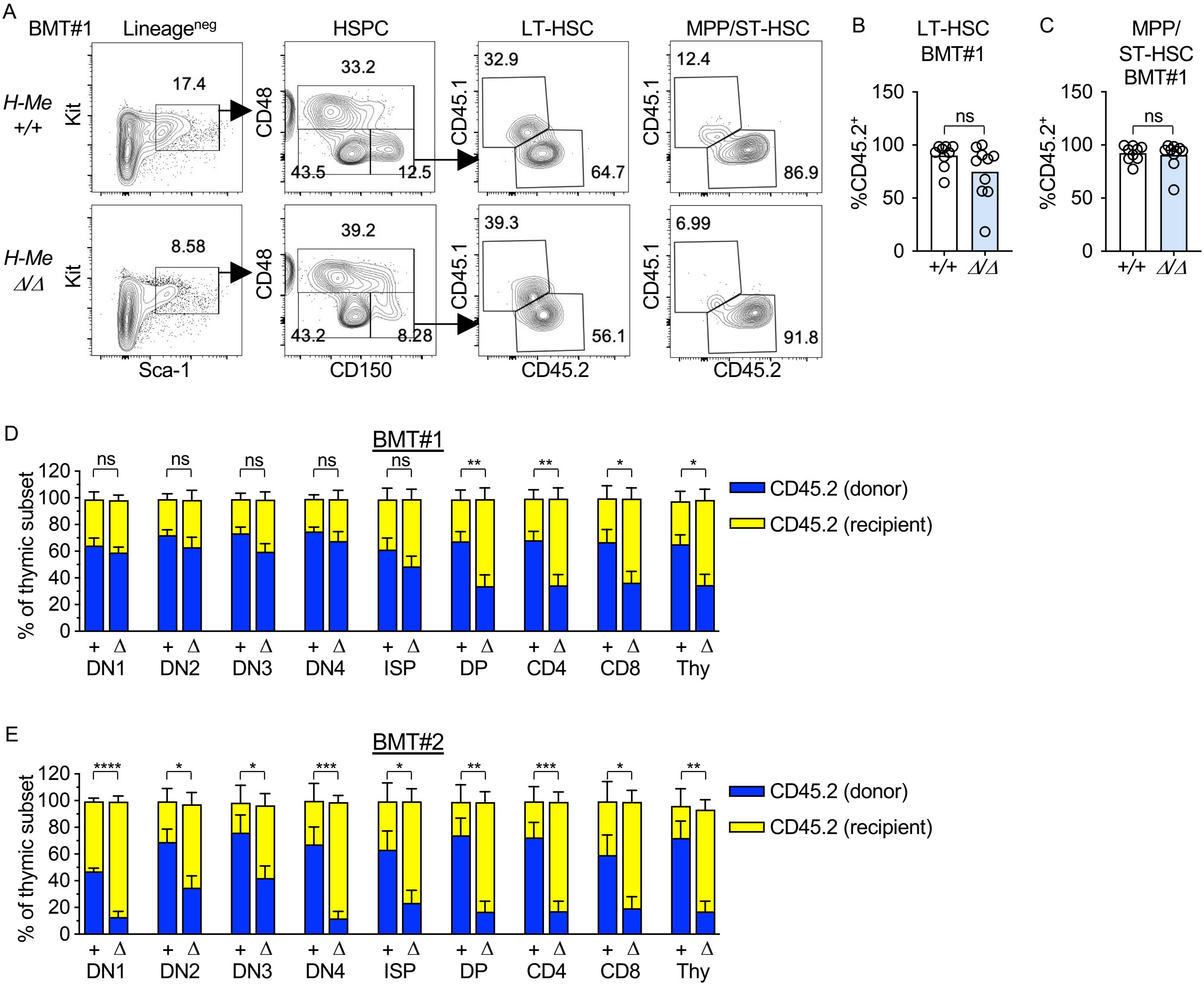

Figure S6. The H-Me is important for long-term HSC self-renewal under stress conditions. A-C) Representative Lineage$^-$ flow cytometry plots (A) and %donor-derived (CD45.2$^+$) analysis of LT-HSC (B) and MPP/ST-HSC (C) cells of *Mx1Cre H-Me*$^{\Delta/\Delta}$ (Δ/Δ) and littermate *Mx1Cre* control (+/+) donor cells at 16 weeks after the first BMT in Fig. 3A. D-E) %donor-derived (CD45.2$^+$) analysis of thymocyte subsets after BMT#1 (D) and BMT#2 (E) in Fig. 3A -- DN1 (Lineage$^-$CD44$^+$CD25$^-$), DN2 (Lineage$^-$CD44$^+$CD25$^+$) DN3 (Lineage$^-$CD44$^-$CD25$^+$), DN4 (Lineage$^-$CD44$^-$CD25$^-$, ISP (CD8$^+$TCRβ$^-$), DP (CD4$^+$CD8$^+$), CD4 (CD4$^+$CD8$^-$TCRβ$^+$), CD8 (CD4$^-$CD8$^+$TCRβ$^+$) and total thymocytes (Thy) of indicated donor cells. P values were obtained using non-transformed data. *P<0.05; **P<0.01; ***P<0.001; ****P<0.0001.

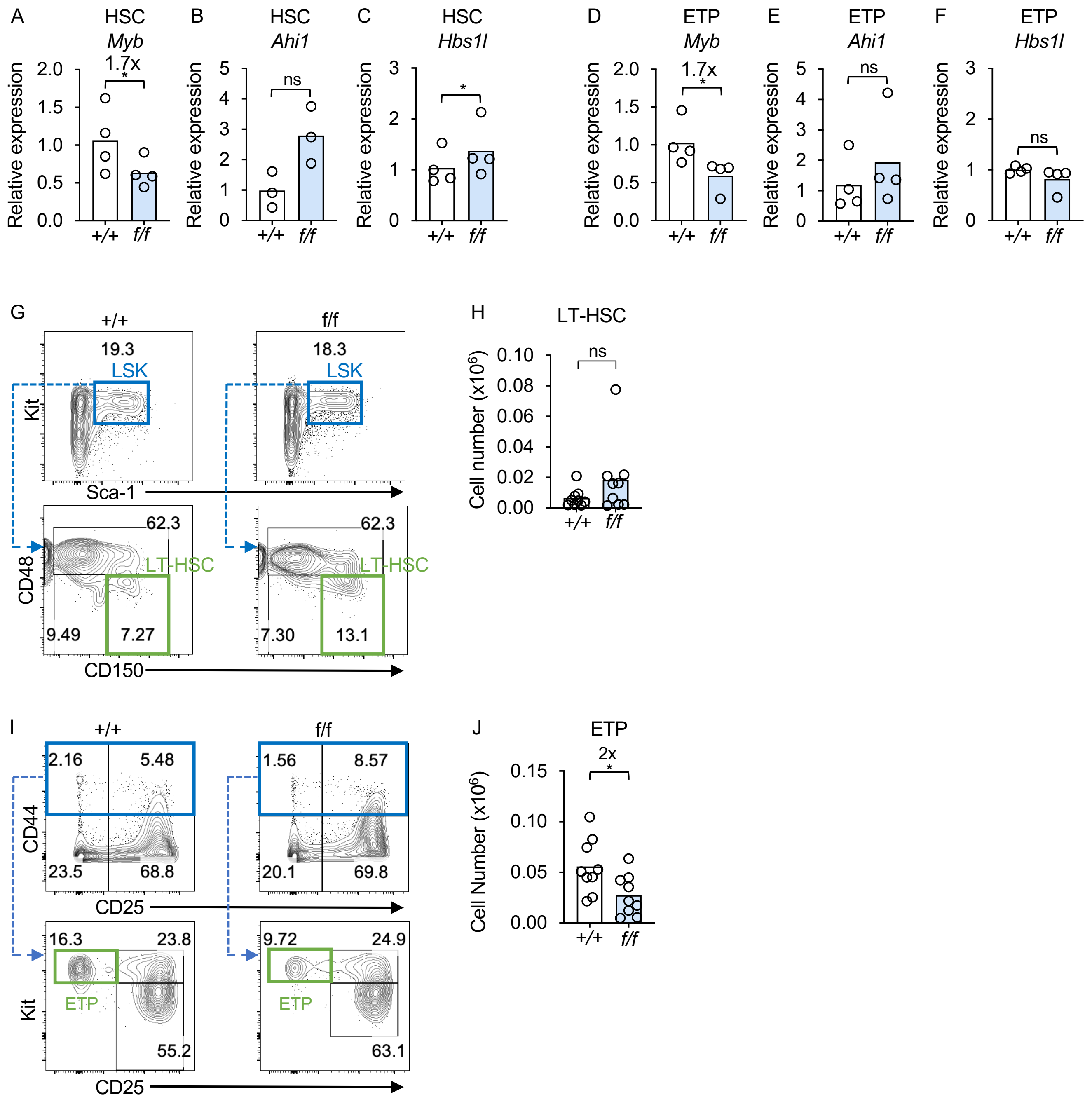

Figure S7. The H-Me floxed allele is hypomorphic. A-F) qRT-PCR of *Myb* (A, D), *Ahi1* (B, E), and *Hbs1l* (C, F) in sorted LT-HSCs (A-C) and ETPs (D-F) from H-Me$^{+/+}$ (+/+) and H-Me$^{f/f}$ (f/f) mice. G-J) Representative flow cytometric plots (G, I) and absolute numbers (H, J) of LT-HSCs (G-H) and ETPs (I-J), Subsets were defined in Fig. 2. 5-8-week-old mice were analyzed. *P<0.05

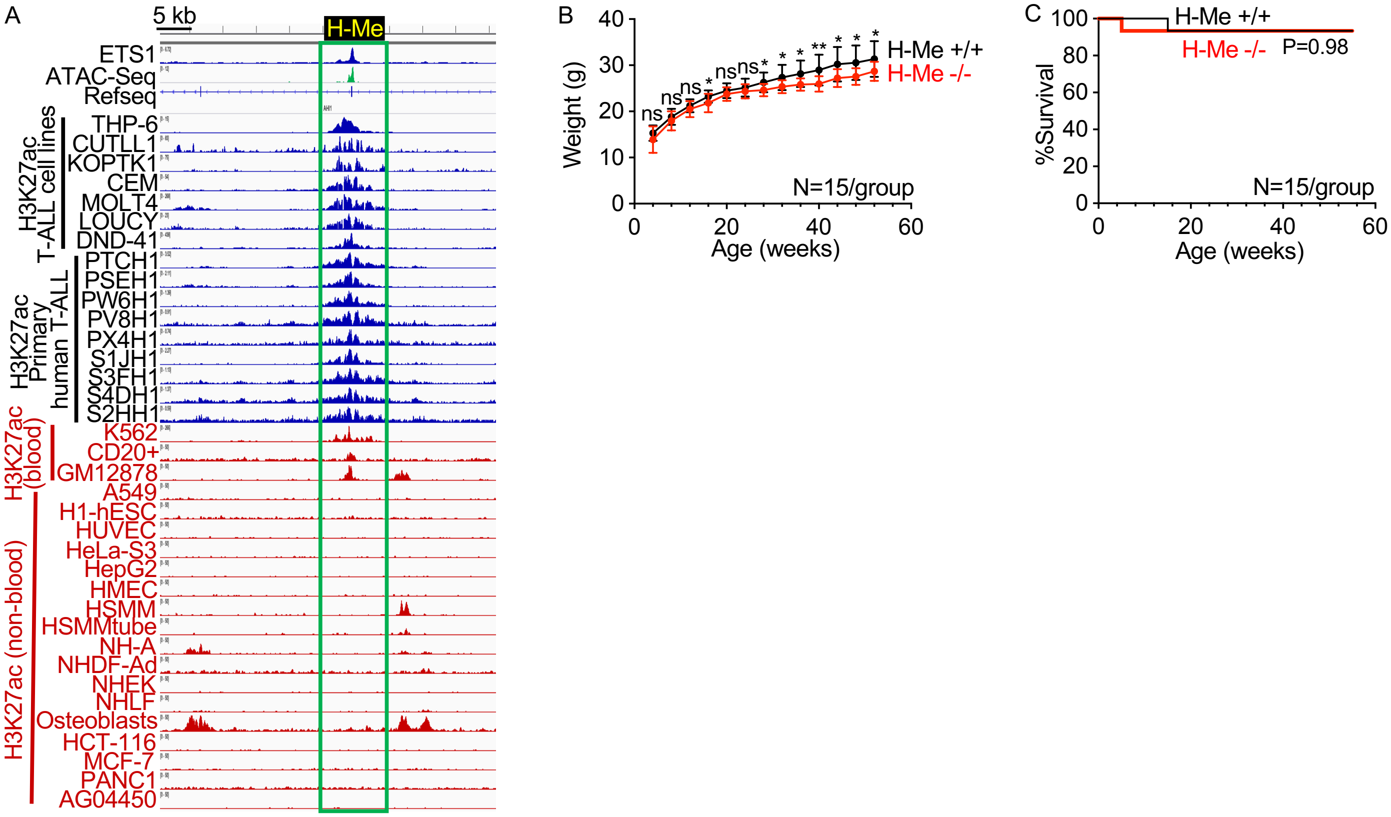

Figure S8. Ubiquitous deletion of the H-Me does not affect survival. A) H3K27ac ChIP-seq profiles at H-Me in T-ALL cells, other blood cells, and non-blood cells. ENCODE datasets are in red. Blueprint primary T-ALL and T-ALL cell line datasets are in blue (GSE76783, GSE29600, and GSE138516). B-C) Weights (B) and survival (C) of *H-Me*$^{-/-}$ (-/-) and littermate control *H-Me*$^{+/+}$ (+/+) mice over one year. *P<0.05.

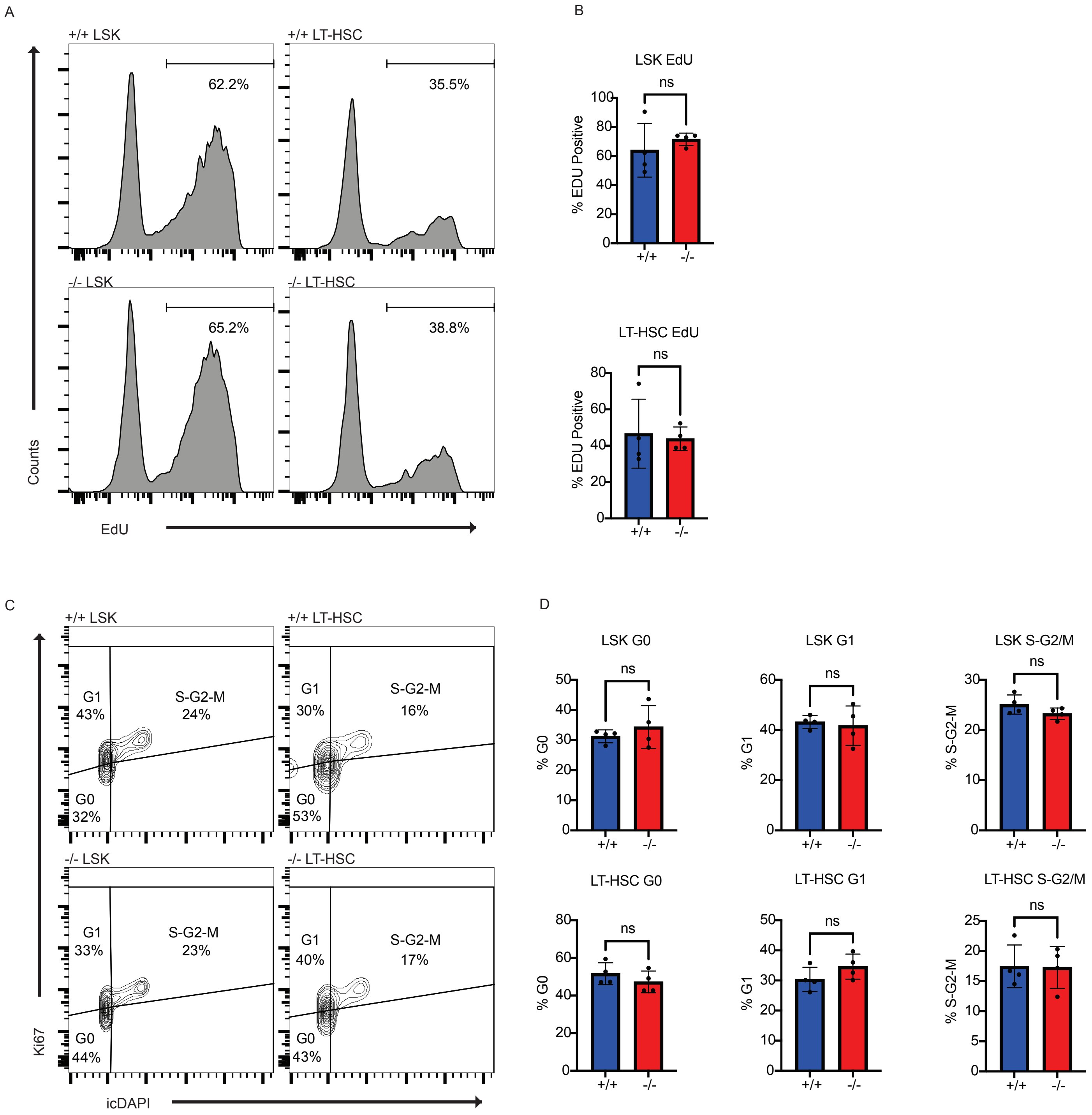

Figure S9. *H-Me*$^{+/+}$ and *H-Me*$^{-/-}$ hematopoietic stem and progenitor cells display comparable cell cycle activity in 10-13-week-old mice. A-B) 10-13-week-old *H-Me*$^{+/+}$ (+/+) and *H-Me*$^{-/-}$ (-/-) mice (N=4/group) were injected with 1mg EdU followed by 72hrs of continuous labeling via drinking water (0.3mg/mL). Bone marrow Lin^{-}Sca1^{+}cKit^{hi} (LSK) progenitors and SLAM LT-HSC populations were measured for %EdU positive cells (representative histograms in (A) and scatterplots in (B)). C-D) LSK and SLAM LT-HSC populations were stained with icDAPI and Ki67 to identify G0, G1, and S-G2-M cell cycle distribution (representative dot plots in (C) and scatterplots in (D)). Mean values were compared with Student's t-test.

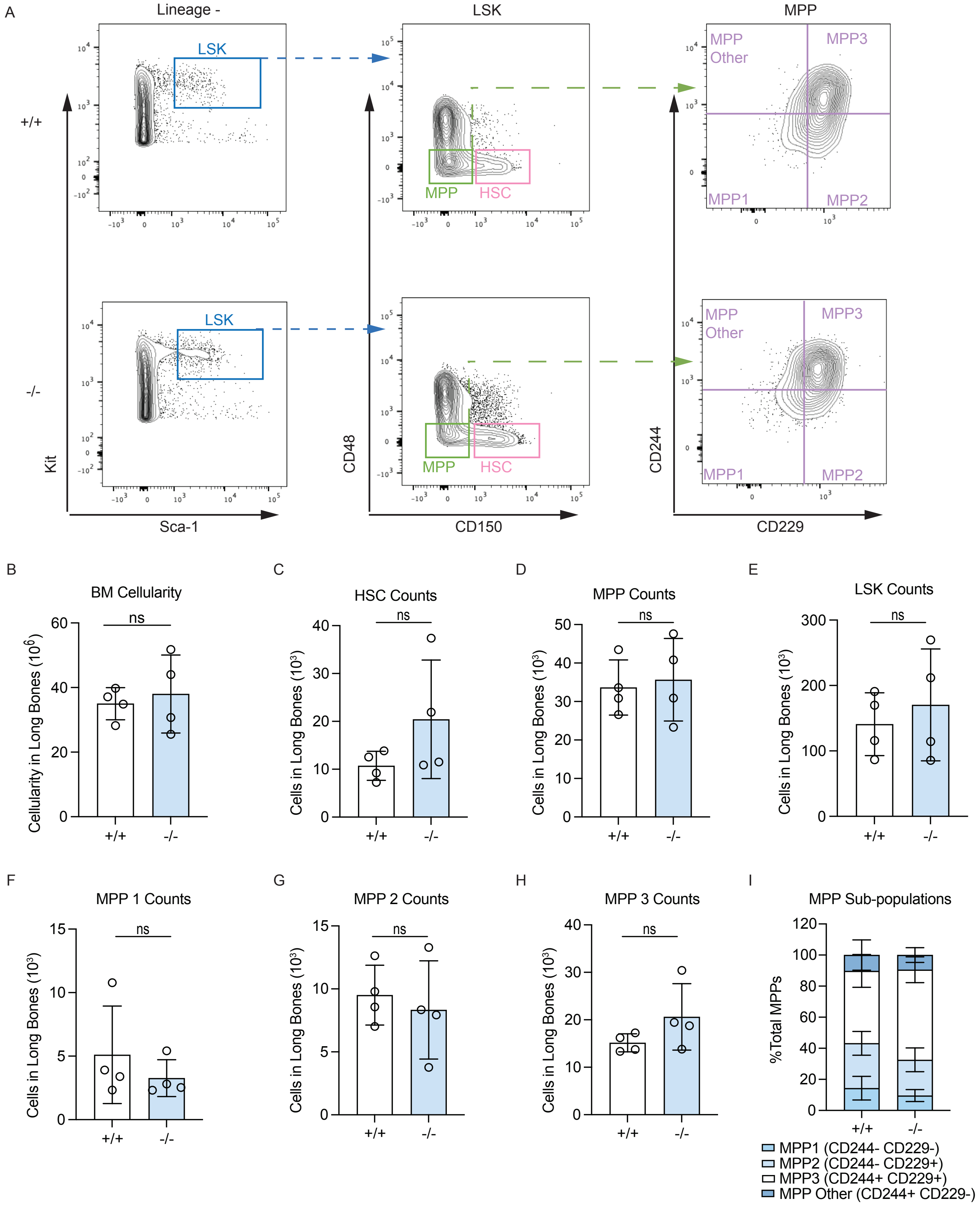

Figure S10. Germline H-Me deficiency does not impact HSPCs at age 10-13 weeks. A) Representative flow plots of HSC and MPP gating for *H-Me*$^{+/+}$ (top) and H-Me$^{-/-}$ (bottom). B) Total cellularity in long bones, calculated for two legs. C-E) Total number of HSCs (C), total MPPs (D), and LSKs (E) in one long bone, respectively. F-H) Total number of MPP1 (F), MPP2 (G), MPP3 (H), respectively, in one leg. I) Percentage of MPP subpopulations, including the undefined, MPP Other, population in the total MPP population.

A

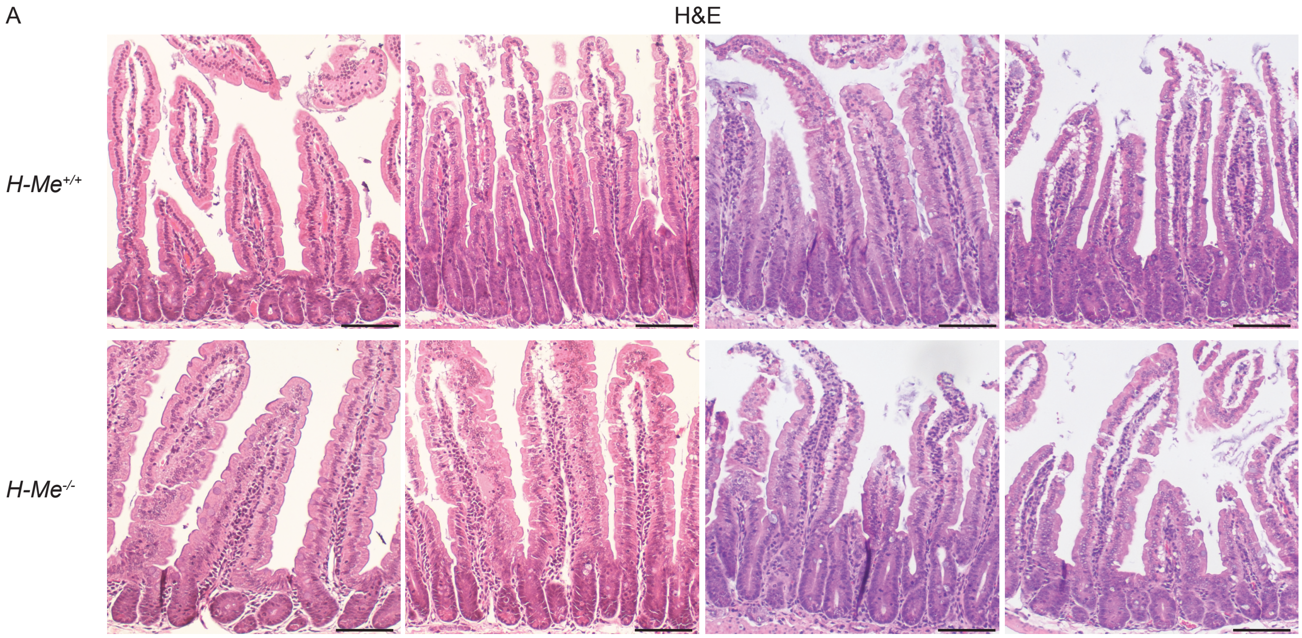

B

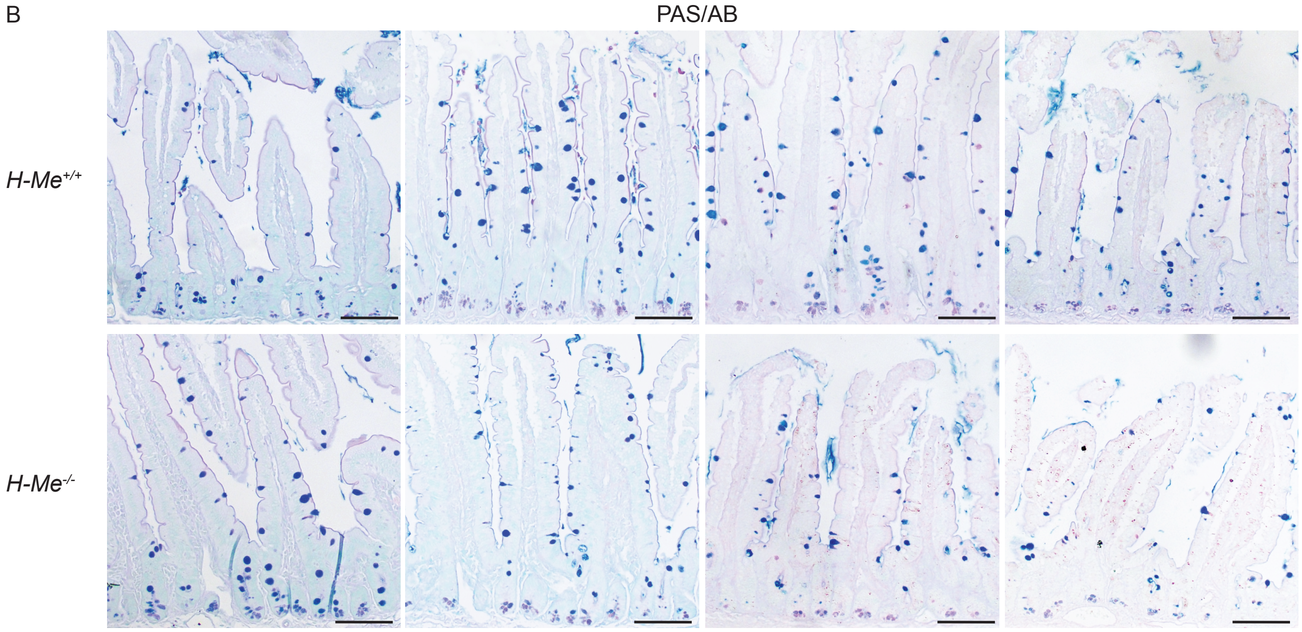

Figure S11. Deletion of the H-Me has no effect on intestine morphology in 10-13-week-old mice. A) H&E staining of duodenal tissue sections from wildtype (*H-Me*$^{+/+}$, top row) and knockout (*H-Me*$^{-/-}$, bottom row) mice. B) PAS/AB staining of duodenal tissue sections from wildtype (*H-Me*$^{+/+}$, top row) and knockout (*H-Me*$^{-/-}$, bottom row) mice. Four individual mice (10-13 weeks of age) from each experimental group are shown. Scale bars = 100μm.

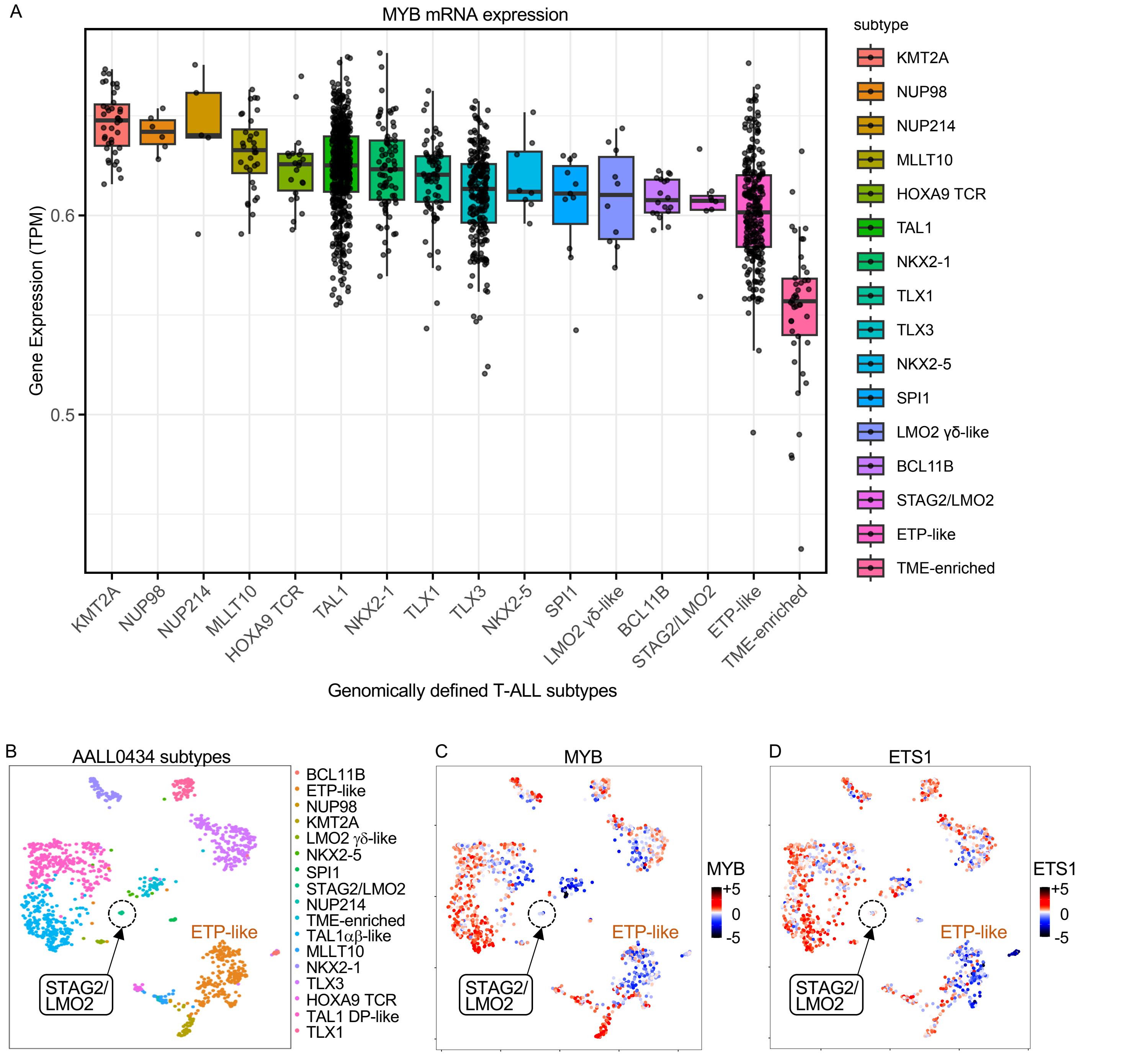

Figure S12. MYB is highly expressed across conventional T-ALL subtypes. A-D) TPM boxplot (A) and UMAP scatterplots (B-D) of AALL0434 patient RNA-seq samples (5) grouped by genomically defined subtypes (B, adapted from(6)) showing expression of MYB (A, C) and ETS1 (D). Heatmaps of expression Z-scores are shown in (C) and (D). Note that the TME-enriched subtype shows high microenvironmental involvement, which affects gene expression values.

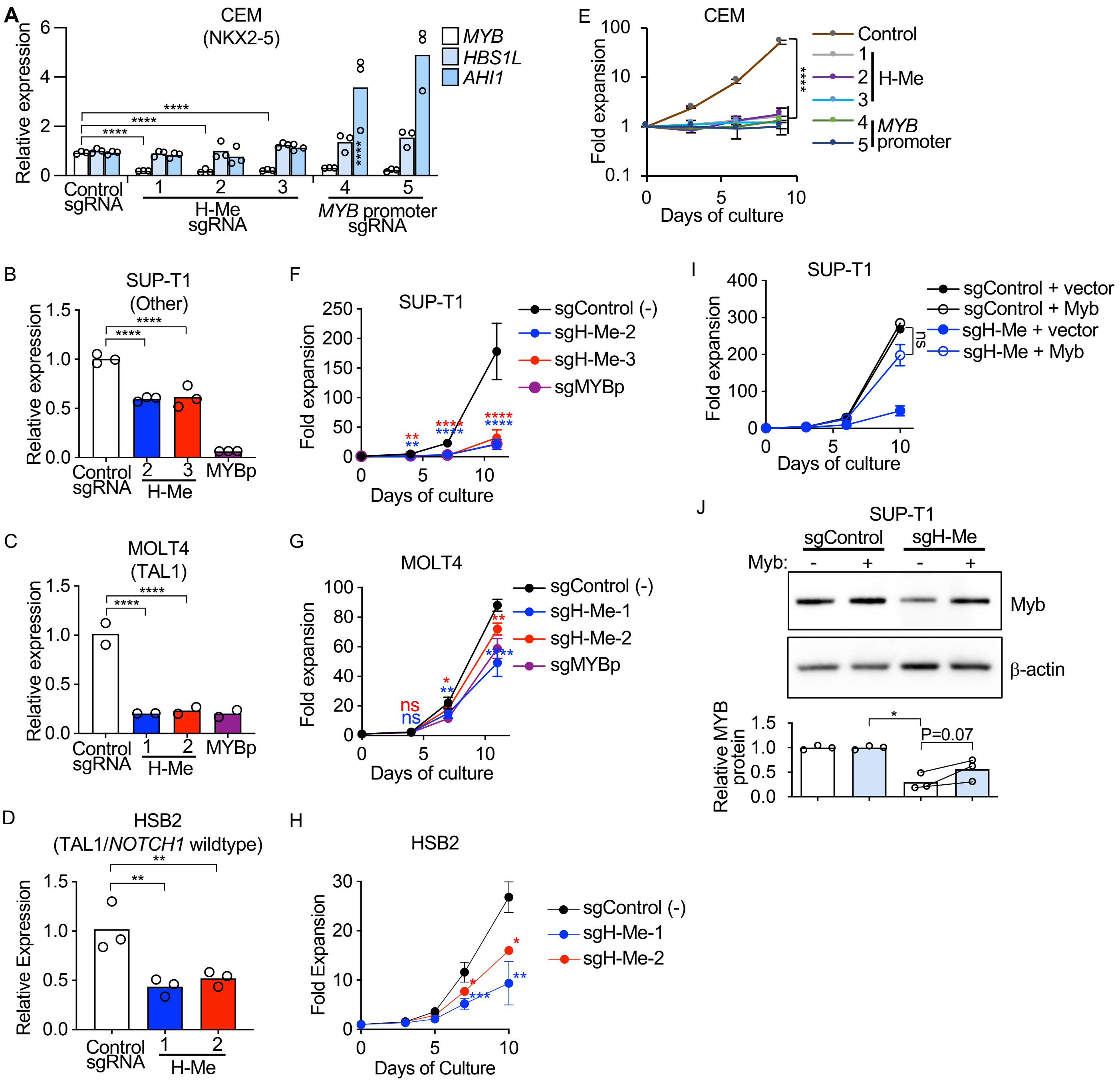

Figure S13. The H-Me promotes cell population growth in diverse conventional T-ALL subtypes. A-H) *MYB* qRT-PCR (A-D) and cell growth assays (E-H) of CEM (related to THP-6; A, E), SUP-T1 (B, F), MOLT4 (C, G), and HSB2 (D, H) cells co-transduced with constitutive (CEM) or doxycycline-inducible (SUP-T1, MOLT4, HSB2) dCas9-KRAB, TET3G, and indicated sgRNAs. Doxycycline was added 1 day after transduction. RNA was harvested 4 days after transduction. I-J) Growth assay (I) and MYB Western blot (J) on the 6th day of doxycycline treatment of SUP-T1 cells transduced with *Myb*, doxycycline-inducible dCas9-KRAB, TET3G, and sgRNAs.

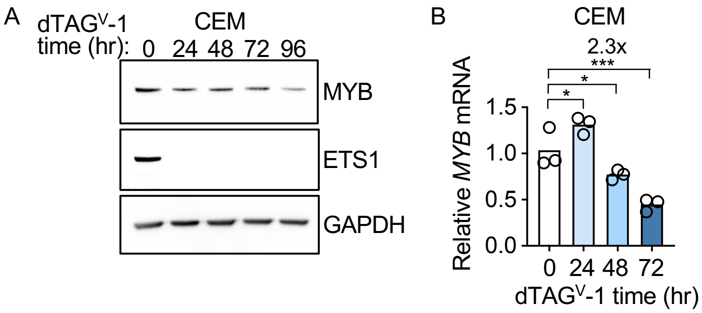

Figure S14. ETS1 regulates *MYB* expression in CEM cells (related to THP-6 cells). A-B) Western blots of ETS1 and MYB proteins (A) and *MYB* qRT-PCR (B) showing the effect of 500nM dTAG^{V}-1 in inducing ETS1 degradation in ETS1-FKBP^{F36V} knock-in CEM cells. *P<0.05; ***P<0.001.

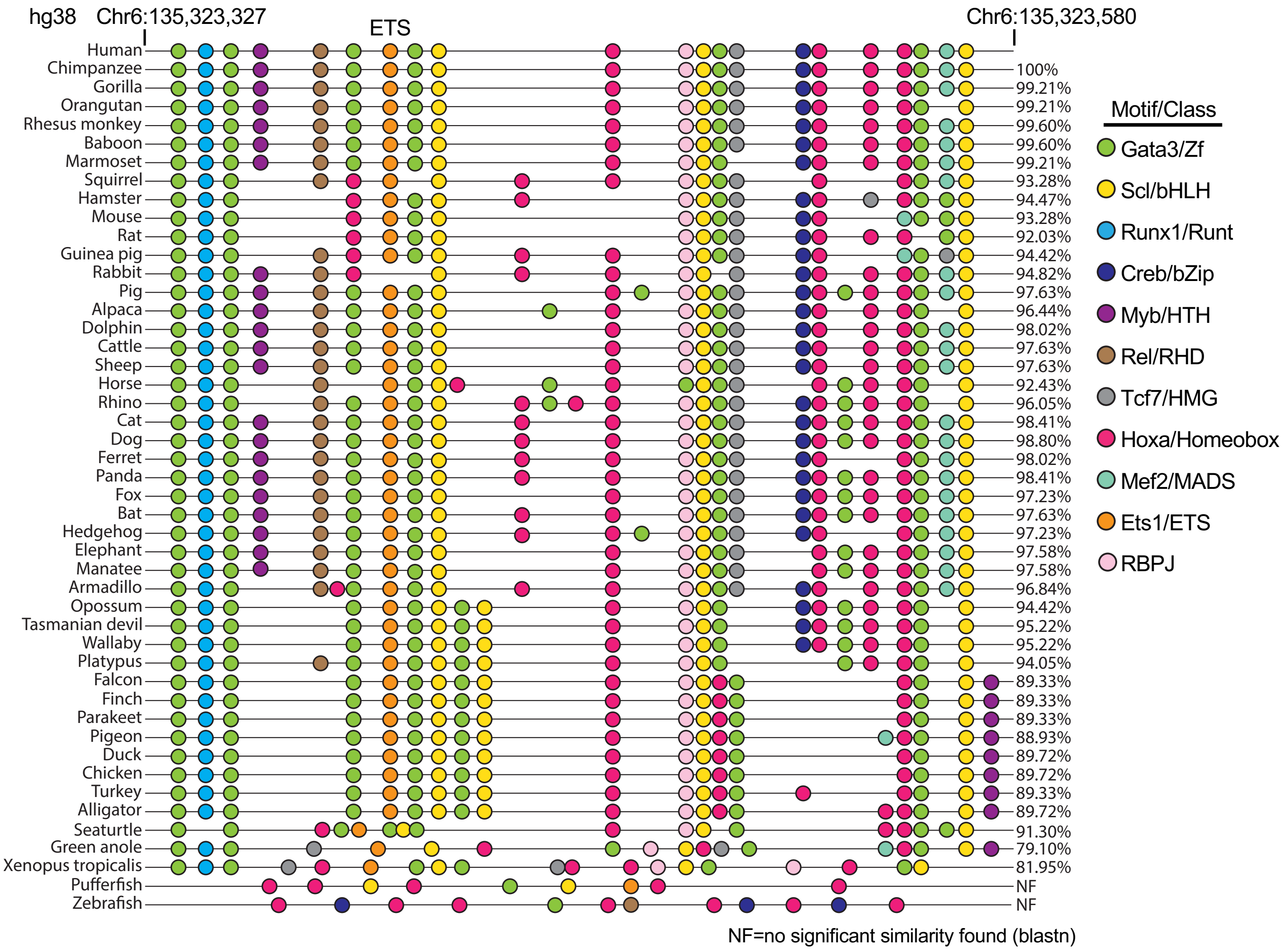

Figure S15. The H-Me is highly conserved, including a single ETS motif. Bulletin board schematic of HOMER motif analyses of the H-Me showing specific motifs/motif classes across several species. Percents represent sequence similarities to human. ETS site marked in orange.

A

H-Me	Sequence of 250bp "Reverse ChIP" probe -- chr6:135,323,327-135,323,580 (hg19)
Wildtype (WT)	AAGAGAGAGATAAGACCACCACAGCTTTATCACAACTGTACCACAGAGAAAACCCCCAACATT<br>CACTTCCTCTTGCAGAAGAGCAGCTGAAACCAAAGCAGTACTTTGGTAAGTGTGAGGCTGTCT<br>CAAGGTTTGCTAATGGGATGAGGGGTGGTGGGAACACAGCAGGAGAAGCAAGATAAAAGACTG<br>TGGGATTGAGAAATGGTTACGAGCATTCAATACACTAAGATTAGCTTCAAAATAGCAGATGTTAA
ETS mutant (m)	AAGAGAGAGATAAGACCACCACAGCTTTATCACAACTGTACCACAGAGAAAACCCCCAACATT<br>----TTCTCTTGCAGAAGAGCAGCTGAAACCAAAGCAGTACTTTGGTAAGTGTGAGGCTGTCT<br>CAAGGTTTGCTAATGGGATGAGGGGTGGTGGGAACACAGCAGGAGAAGCAAGATAAAAGACTG<br>TGGGATTGAGAAATGGTTACGAGCATTCAATACACTAAGATTAGCTTCAAAATAGCAGATGTTAA

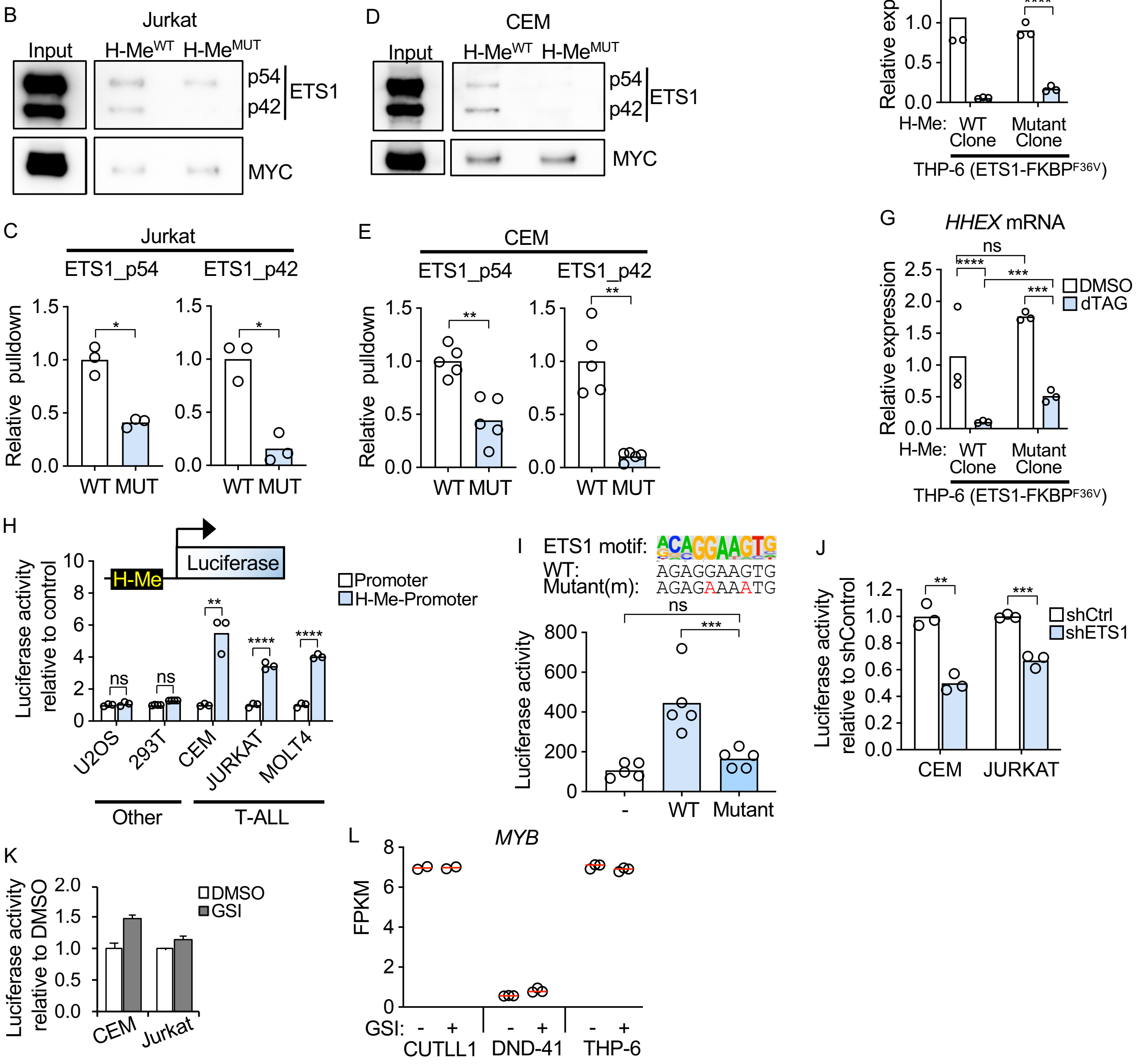

Figure S16. ETS1 binds the H-Me ETS site and activates transcription. A) Sequences of the human wildtype and ETS-mutated H-Me DNA fragments used in "reverse ChIP" and reporter assays. B-E) Representative Western blots (B, D) and quantitative Image J analyses (C, E) showing the effect of the ETS motif mutation on ETS1 binding (p54 and p42 isoforms) in "reverse ChIP" compared to MYC as a control in in Jurkat cells (B-C) and CEM cells (D-E, related to THP-6 cells). F-G) Expression of ETS1 target genes *LYL1* (F), and *HHEX* (G) in a subclone of ETS1-FKBP^{F36V} knock-in THP-6 cells after homozygous CRISPR/Cas9 gene editing and homology directed repair creating homozygous partial ETS site mutations (Fig. 8F). H-J) Luciferase reporter assays measuring the effect of the H-Me (A) in a panel of cell lines (H), the effect of mutating the ETS site in CEM cells in this reporter (I), and the effect of *ETS1* knockdown on this reporter using previously reported shETS1 constructs (1) in indicated T-ALL cell lines (J). K) H-Me luciferase assay on indicated cell lines treated with 1mM GSI (DBZ) or DMSO control. L) *MYB* FPKMs in indicated T-ALL cell lines treated with DMSO or GSI (GSE90715; GSE116873; GSE138659).

Fig. S17

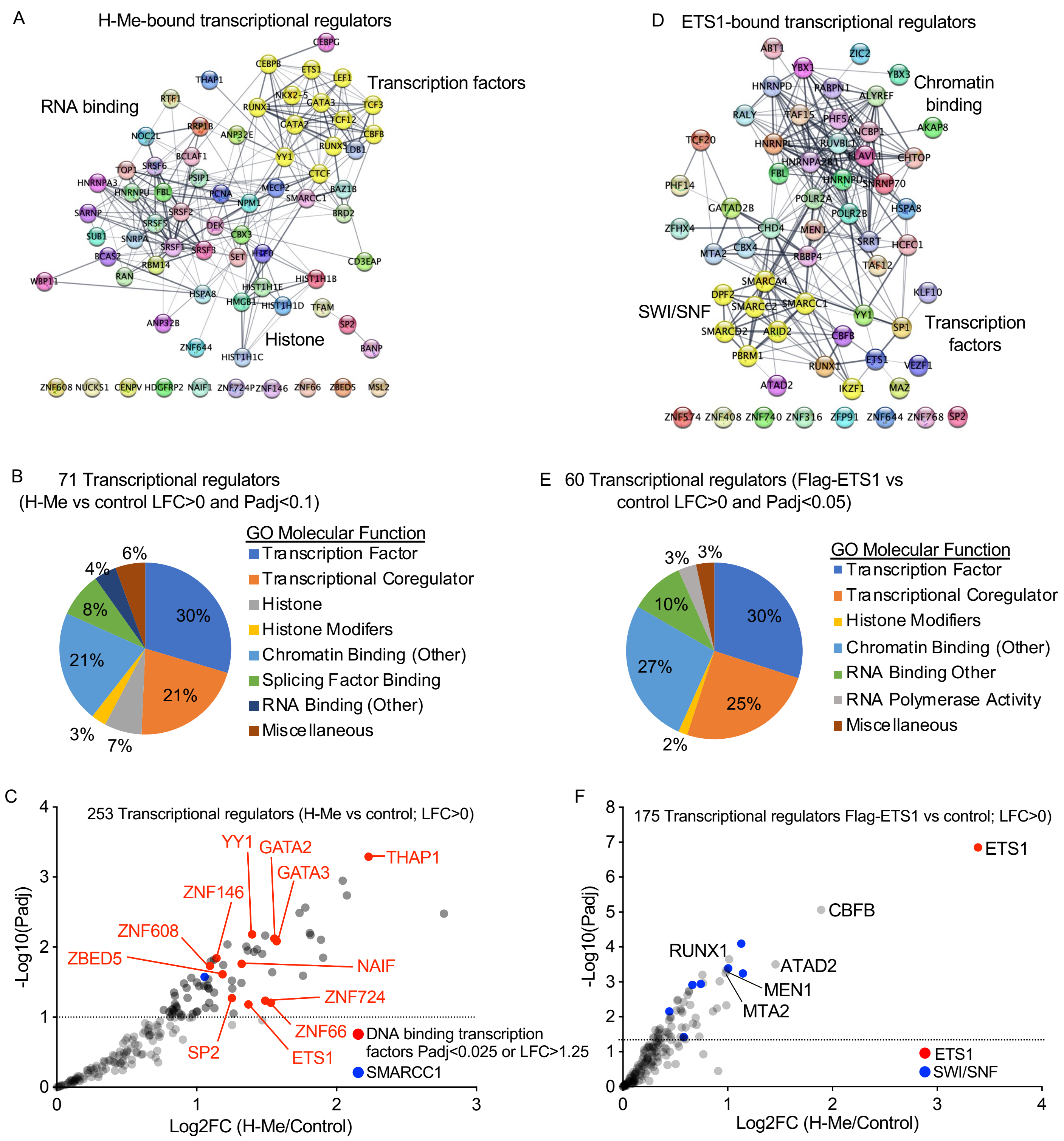

Figure S17. The SWI/SNF complex is a top-ranked candidate cofactor that binds the H-Me and ETS1. A-C) Cytoscape String plot (A), Gene Otology (GO) molecular functions (B) and volcano plot (C) of the transcriptional regulators that were differentially pulled down by H-Me "reverse ChIP" in CEM cells relative to control beads based on FragPipe analysis. Highlighted genes in (A) are first neighbors of ETS1. D-F) Cytoscape String plot (D), GO function (E) and volcano plot (F) of the transcriptional regulators that were differentially pulled down by Flag antibody co-IP comparing Flag-ETS1-transduced and vector control-transduced CEM cells based on FragPipe analysis. Yellow cluster (D) and blue dots (F) are cBAF and/or PBAF subunits.

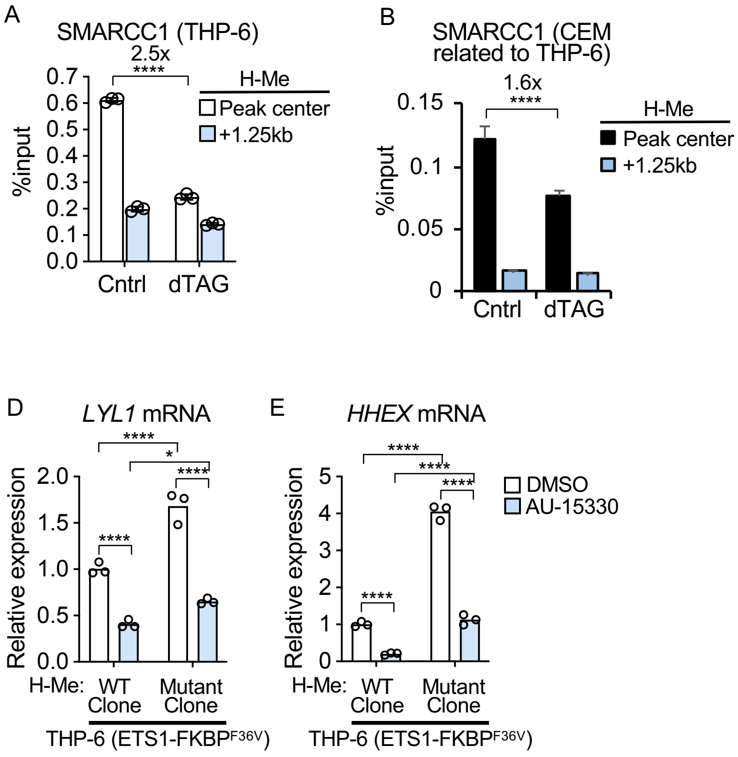

Figure S18. ETS1 recruits the cBAF complex to activate the H-Me in THP-6/CEM cells. A-B) SMARCC1 qChIP using primers at the H-Me peak center or a negative control site 1.25kb downstream in ETS1-FKBP^{F36V} knock-in THP-6 cells (A) or CEM cells (B, related to THP-6 cells) treated in triplicate with DMSO control or 500nM dTAG^{V}-1 (dTAG) to degrade ETS1. C) H3K27ac qChIP in ETS1-FKBP^{F36V} knock-in THP-6 cells treated in triplicate with DMSO control or 500nM dTAG^{V}-1 (dTAG) to degrade ETS1. D-E) *LYL1* (D), and *HHEX* (E) qRT-PCR in ETS1-FKBP^{F36V} knock-in THP-6 cells with ETS-binding site mutant H-Me (Fig. 8F) treated with DMSO vs AU-15330. *P<0.05; ****P<0.0001.

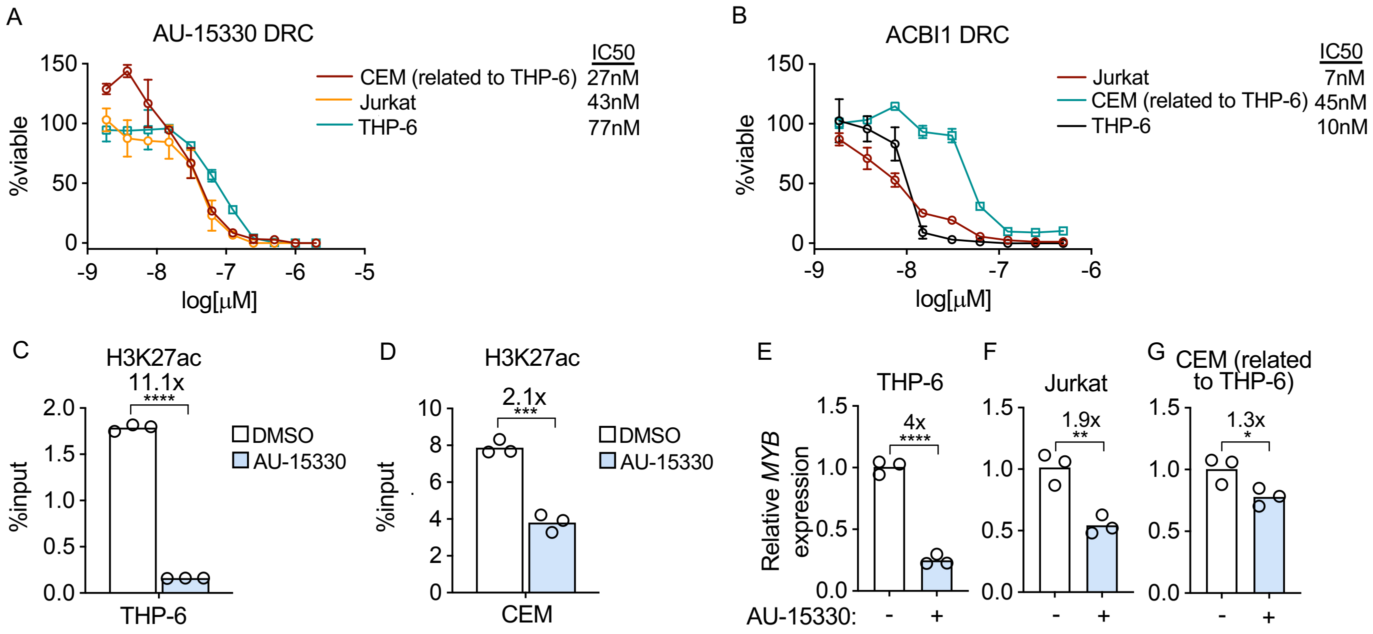

Figure S19. cBAF inhibitors inactivate the H-Me and downregulate *MYB* in two T-ALL contexts. A-B) Dose response curves of indicated T-ALL cell lines treated with SMARCA2/4 PROTACs AU-15330 (A) and ACBI1 (B). C-D) H3K27ac qChIP of DMSO-treated or AU-15330-treated THP-6 cells (C) or CEM cells (D, related to THP-6). E-G) *MYB* qRT-PCR of DMSO-treated or AU-15330-treated THP-6 cells (E), Jurkat cells (F) or CEM cells (G, related to THP-6). *P<0.05; **P<0.01; ***P<0.001; ****P<0.0001.

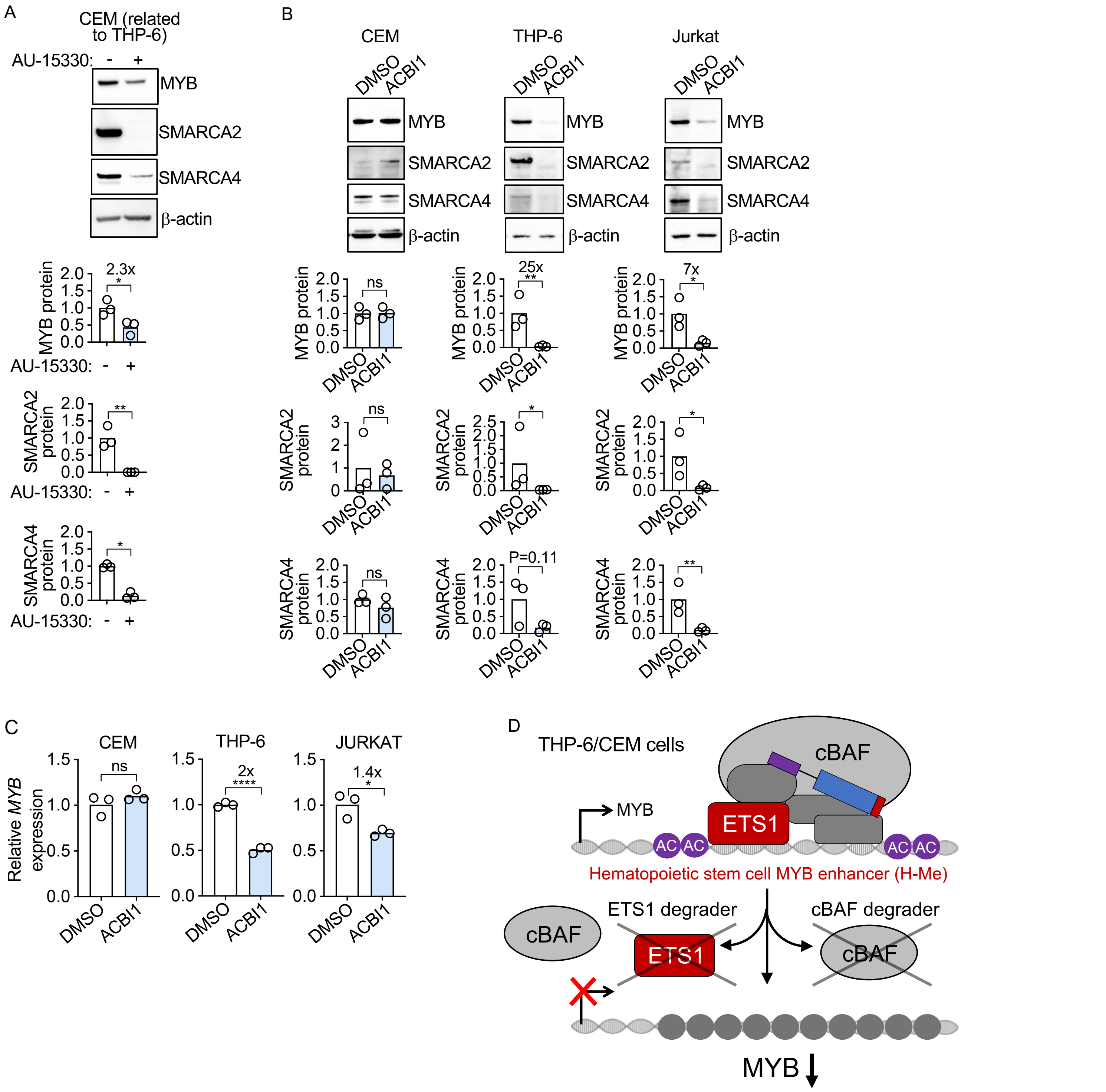

Figure S20. cBAF inhibitors downregulate *MYB* in two T-ALL contexts. A) Western blot for indicated proteins of DMSO-treated or AU-15330-treated CEM cells (related to THP-6). B-C) Western blot for indicated proteins (B) and *MYB* qRT-PCR (C) of DMSO-treated or ACBI1-treated CEM, THP-6, and Jurkat cells. C) Model of the H-Me in THP-6/CEM cells. ETS1 recruits cBAF to the H-Me to remodel chromatin, which facilitates transcription regulator binding and activation of this enhancer (H3K27ac deposition) in THP-6 T-ALL cells. ETS1 and cBAF degraders inactivate the H-Me, resulting in decreased *MYB* expression. Model is based on this manuscript and McCarter et al (1). *P<0.05; **P<0.01.

Supplemental methods

Study approval

Mouse experiments were performed according to NIH guidelines and approved protocols from the Institutional Animal Care and Use Committee at the University of Michigan (Ann Arbor).

Gene editing

ETS1-FKBP^{D36V} cell lines were generated using a ETS1^{HDR}-FKBP^{F36V}-2xHA-P2A-mCherry template (constructed based on the protocol from(7), Alt-R^{TM} Cas9 protein (Catalog Number: 1072533), Alt-R^{TM} tracrRNA (Catalog Number: 1072533), and custom Alt-R^{TM} tracrRNA sgRNAs purchased from IDT and electroporated into THP-6 and CEM/SS cells. 24 hours prior to electroporation, cells were treated with 10mM Farrerol (Selleck Chem, Catalog Number: S9552) and left to incubate overnight. 100uM Alt-R CRISPR-Cas9 tracerRNA and 100uM Alt-R CRISPR-Cas9 crRNA were heated at 95C for 5 minutes. 50mM of the above complex and 62mM of the Alt-R Cas9 Enzyme were incubated at room temperature for 10-20 minutes. Cells were resuspended in Buffer R from the Invitrogen Neon Transfection Kit (Catalog Number: MPK1096B) to reach a final concentration of 20million cells/mL. Electroporation was performed with Neon^{TM} transfection system (Fisher, Catalog Number: NEON1SK) using the following conditions: 2.4 x 10^5 farrerol-treated cells, 60pmol RNP complex, and 1.25ug ETS1^{HDR}-FKBP^{F36V}-2xHA-P2A-mCherry in Neon^{TM} buffer R to final volume of 10uL; Pulse Voltage: 1325V, Pulse Width: 10ms, Pulse Number: 3. Single cell colonies were screened for homozygous recombination by PCR. Knockdown experiments were performed on homozygous recombinants by supplementing media with 500nM dTagV-1 (Biotechne, Catalog Number: 6914) for 24-96 hours. Western blot was performed as previously published (1). Sequence editing of the ETS H-Me binding site in ETS1-FKBP^{F36V} THP-6 cells was carried out using the aforementioned protocol using Alt-R^{TM} Cas9-GFP (Catalog number: 10008100) and addition of 5mM Alt-R^{TM} HDR template sequence (Catalog

number: 1072533) into the electroporation reaction. 24 hours later, GFP expressing cells were sorted by FACS. 5-7 days later, the population was single cell cloned by limiting dilution.

CRISPRi screening

Previously published ATAC-seq and H3K27Ac ChIP-seq datasets (1) for THP-6 cells was used to score enhancers by "Activity-by-Contact" score (8). Elements with high "Activity" scores (>5, 14,080 elements) were intersected with dynamic ETS1 intervals. Dynamic ETS1 enhancers were defined as genomic sites with significantly decreased (Log2FC<-0.5, FDR<0.05) H3K27Ac and ETS1 ChIP-seq signal upon *ETS1* knockdown in THP-6 cells (1). Homer annotatePeaks(9) was used to determine H3K27Ac ChIP-seq signal normalized to 10 million reads across these intervals in 9 primary T-ALL samples (Blueprint), and elements with low mean ChIP-seq intensity<25 were eliminated. FlashFry (10) was used to identify and score sgRNA sequences across the remaining 1,433 elements. sgRNAs were filtered to retain sgRNAs with low predicted off-target and high predicted on-target effects, and downsampled to 20 sgRNA per 1kB. 866 Non-targeting controls and 200 pan-essential gene targeting controls ("Dolcetto" genome-wide library (11)) were included as previously described (12). sgRNA library cloning was performed as previously described (12). CRISPRi negative selection screen, including sequencing library preparation and analysis was performed as previously described with 3 replicates for each time point and 400X coverage (13) with the exception that doxycycline-dependent dCas9-KRAB expressing THP-6 cells were used. Samples were collected before addition of doxycycline (T0) and after 15 doublings (T15). Analysis was performed using MaGeCK-RRA software (14) with Log2FC normalization to non-targeting control sgRNAs. Primer sequences are in Table S1.

Viral production

High titer retroviral or lentiviral supernatant was produced using transient transfection of 293T cells (RRID: CVCL_0063) and assessed for GFP or NGFR titer by transducing 8946 cells and measuring %GFP and %NGFR two days later.

Mouse intestine analysis

Duodenal tissue was fixed overnight in 4% paraformaldehyde, processed, and paraffin embedded as previously described (1). Sections (4mm) were deparaffinized, and either H&E stained (Vector H&E kit, H-3502), or PAS/AB stained (Newcomer Supply: PAS kit 9162B and AB 1003A) according to the manufacturer's directions.

Human T-ALL xenografts

CRISPRi cells were lentivirally transduced with sgRNAs within the sgOPTI backbone and cultured in puromycin (2-5µg/mL) for 3-5 days. 2-3 million cells per mouse were tail IV injected into age and sex-matched NOD *scid* gamma (NSG) mice. 2-5 days later, mice were started on 2g/L doxycycline water (doxycycline HCl, Research Products International, CAS 10592-13-9) with 50g/L sucrose. Peripheral blood blast count was analyzed by tail vein bleeding and flow cytometric analysis for human $CD45^+$ GFP^+ cells.

Cell cycle staining

EdU. Mice were injected with 1mg 5-Ethynyl-2'-deoxyuridine (EDU; Cayman Chemical) and then placed on 0.3mg/mL EDU dissolved in drinking water for 72hrs. Following the labeling period, mice were sacrificed and bone marrow was harvested from spine, hips, and legs via grinding with mortar and pestle. Samples were then filtered with 60uM filter and incubated for 20 minutes on ice with anti-cKit magnetic beads (Miltenyi). Samples were then washed twice in HBSS + 2% bovine calf serum (BCS) and cKit positive cells were separated using an autoMACS Neo separator (Miltenyi). The positively isolated fraction was stained with the following antibodies:

CD2-PE, CD3-PE, CD5-PE, CD8-PE, Gr1-PE, TER119-PE, B220-PE, cKit-APC, CD48-AlexaFluor700, and CD150-PE-Cy7 (all from Biolegend) for 30 minutes. Samples were washed and then fixed using the eBioscience FOXP3/Transcription Factor kit (Invitrogen) per manufacturer protocol. Samples were then subjected to Click-iT Plus Alexa Fluor 488 picolyl azide reaction (Invitrogen) per manufacturer protocol. Samples were washed and intracellular DAPI labeling was then performed (1mg/mL DAPI diluted 1:100 in FoxP3 Perm/Wash buffer for 20 minutes at room temperature in the dark). Samples were then washed 2X in HBSS + 2% BCS and analyzed on a BD LSRFortessa.

Ki67. Bone marrow was prepared and cKit+ cells were isolated as above. Cell surface staining and fixation were performed as above. Following fixation, samples were stained with antiKi67-FITC (Biolegend) at a 1:10 dilution in Perm/Wash buffer for 40 minutes at RT in the dark. Samples were then washed with Perm/Wash buffer and intracellular DAPI staining was performed as above.

Mx1Cre bone marrow transplantation

Bone marrow stem and progenitor cells of *Mx1Cre H-Me*$^{+/+}$ *or Mx1Cre H-Me*$^{f/f}$ mice were transduced with an activated Notch1 allele (*ΔE/Notch1*). Transduced cells were injected into irradiated C57BL/6 mice to generate primary T-ALL tumors as previously described (1, 2, 15). Five weeks post-transplant, recipient mice were injected with 40ug pI-pC (GE Healthcare, Catalog Number: 27-4732-01) five times every other day totaling 200μg pI-pC injected.

H-Me knockout bone marrow transplantation

Bone marrow stem and progenitor cells of *H-Me*$^{+/+}$ or *H-Me*$^{-/-}$ mice were transduced with an activated Notch1 allele (ΔE/Notch1). Transduced cells were injected into irradiated C57BL/6 mice to generate primary T-ALL tumors as previously described (1, 2, 15).

Competitive bone marrow transplantation

4-week-old Mx1-Cre *H-Me*$^{+/+}$ or Mx1-Cre *H-Me*$^{f/f}$ mice were injected with 40ug pI-pC five times every other day totaling 200ug. 4 weeks post pI-pC injection, a 3:1 ratio of experimental (CD45.2) / competitor cells (CD45.1) were injected into irradiated 564 B6-Ly5.1/Cr mice for primary transplants. 4 months post primary transplant, whole bone marrow from primary recipients was injected into irradiated 564 B6-Ly5.1/Cr mice for secondary transplants.

Flow cytometry

Flow cytometry and cell sorting was performed as previously described (15).

Quantitative RT-PCR

Quantitative RT-PCR was performed as previously published (15).

Luciferase reporter assay

H-Me^{WT}, H-Me^{Mut} constructs and pDMPolI 1 (Renilla) were transfected into CEM/SS cells using DMRIE-C transfection reagent (Invitrogen, Catalog Number: 10459014). 48-hours post transfection, Luciferase activity was determined using the Dual-Luciferase® Reporter Assay System (Promega, Catalog Number: E1910) and GloMax Illuminator.

ChIP-seq library preparation, sequencing, alignment, filtering, motif analysis, display file generation, and peak calling

ChIP-Seq library preparation, sequencing, alignment, filtering, motif analysis, track generation, peak calling, and overlap analysis was performed as previously published (1).

"Reverse ChIP"

Reverse ChIP was performed as previously described (16). Human T-ALL cells were lysed and purified for nuclear protein using ThermoFisher NE-PER kit (catalog number: 78833) and incubated with Dynabeads™ M-280 Streptavidin (Fisher, Catalog Number: 11205D) in Buffer G (20mM Tris pH7.4, 10% Glycerol, 0.1M KCl, 0.2mM EDTA, 10mM potassium glutamate, 0.04% Igepal, 2mM DTT) for 1 hour at 4C to remove non-specific DNA binding factors. H-Me^{WT} and H-Me^{Mut} constructs were PCR amplified with biotinylated tagged primers to create a double-stranded DNA fragment (chr6:135,323,327-135,323,580) and incubated with Dynabeads™ M-280 Streptavidin for three hours in Buffer DW (20mM Tris pH7.4, 10% Glycerol, 0.1M KCl, 0.2mM EDTA, 10mM potassium glutamate, 0.04% Igepal, 2mM DTT). DNA-conjugated streptavidin beads were then blocked using Blocking Buffer (20mM HEPES-NaOH, 0.05mg/mL BSA, 0.3M KCl, 0.02% NP-40, 5mg/mL polyvinylpyrrolidone, 0.05mg/mL glycogen, 2.5mM dithiothreitol) at room temperature for 1 hour and then washed with Buffer DW. Blocked DNA-conjugated streptavidin beads were then incubated with cleared nuclear protein overnight at 4C and washed with Buffer G. For tandem mass tagged and multiplexed spectrometry, beads were washed with PBS and submitted to University of Michigan Proteomics Resource Facility (PRF) with TMT™ Isobaric Label Reagent Set (Fisher, Catalog Number: PI90061). For western blot analysis proteins were eluted using 4x NuPAGE™ LDS Sample Buffer (Invitrogen, Catalog Number: NP0008) and boiled at 95°C for 5 minutes.

FLAG co-immunoprecipitation

The FLAG-ETS1 construct was transduced into CEM/SS cells and sorted for purity. Cells were then lysed in lysis buffer (10% Glycerol, 150mM NaCl, 50mM HEPES.NaOH, 2mM EDTA, 0.1%

Igepal) and centrifuged to remove cell debris. Lysates were then incubated with EZview anti-FLAG beads (Sigma, Catalog Number E6779) overnight and washed with lysis buffer. For mass spectrometry-based proteomics analysis, beads were washed with PBS and submitted to University of Michigan Proteomics Resource Facility (PRF). Samples were analyzed in triplicate (3 ETS1 bait immunoprecipitations and 3 controls). Cell lysates were digested with trypsin, the resulting peptide samples labeled with the TMT™ 6-plex Isobaric Label Reagent Set (Fisher, Catalog Number: PI90061) and analyzed using a Thermo Fisher Orbitrap Fusion mass spectrometer. For western blot analysis proteins were eluted using 4x NuPAGE™ LDS Sample Buffer (Invitrogen, Catalog Number: NP0008) and boiled at 95°C for 5 minutes.

Endogenous co-immunoprecipitation

CEM/SS cells were lysed in lysis buffer (10% Glycerol, 150mM NaCl, 50mM HEPES.NaOH, 2mM EDTA, 0.1% Igepal) and centrifuged to remove cell debris. Lysates were then incubated with 20ug SMARCC1 antibody (CST, Catalog Number: 11956) overnight. 24-hours later lysates were incubated with Dynabeads™ Protein G (Invitrogen, Catalog Number: 10004D) for 2 hours followed by magnetic pull down and lysis buffer wash. Proteins were eluted using 4x NuPAGE™ LDS Sample Buffer (Invitrogen, Catalog Number: NP0008) and boiled at 95°C for 5 minutes.

Mass spectrometry data analysis

The acquired MS data from both the H-Me pull-down and Flag-ETS1 co-IP experiments were analyzed using the FragPipe (17-19) computational platform (v20.1). Mass spectrometry raw data were converted into mzML format using the MSconvert tool from the ProteinWizard software suite, and MS2 spectra were searched using MSFragger (v3.8) against the Uniprot human reference proteome database appended with an equal number of decoy sequences. The search was restricted to tryptic peptides, allowing up to two missed cleavage sites. Carbamidomethylation of cysteine (57.02146 Da) and TMT labeling of lysine (229.16293 Da) were specified as fixed

modifications. Oxidation of methionine (15.9949 Da), N-terminal protein acetylation (+42.0106 Da) and TMT labeling of both peptide N-terminus and serine (229.16293 Da) were specified as variable modifications. The rest parameters were kept as default settings. The search results were further processed using MSBooster for rescoring and Percolator for validation. The resulting Pin files were processed by Philosopher to assemble peptides to proteins and apply the 1% FDR filtering at PSM, peptide and protein levels. The PSM output file from Philosopher were further processed using TMT-Integrator to generate summary reports for protein and peptide quantification. the gene level quantification report (abundance_gene_MD.tsv) was used for downstream analysis. Differential analysis was performed using the linear model provided by the limma package. The log2 fold change (log2FC or "LFC" in the figures) was represented by limma's moderated t-statistic, and a moderated p-value of 0.01 was used to identify up and down regulated proteins. Gene Ontology (GO) analysis was performed with ClusterProfiler, examining the differential expression results. In addition, the H-Me pull-down mass spectrometry data was also analyzed using Thermo Fisher's Proteome Discoverer (PD) with default parameters for TMT6 analysis, followed by the same downstream analysis. List of transcriptional regulators used to filter the mass spectrometry results is shown in Table S6.

ATAC-seq library preparation

Cell suspensions were brought to the UM Epigenomics Core for ATAC-Seq library preparation. We used the Omni-ATAC-Seq for cells protocol (20). Briefly, 50,000 cells were pelleted and resuspended in ATAC-RSB supplemented with Digitonin, Tween-20, and IGEPAL-CA 360. Cells were incubated on ice for 3 minutes before addition of ATAC-RSB with Tween-20, and centrifugation at 500RCF for 10 min at 4C to pellet nuclei. After carefully removing the supernatant, nuclei were resuspended in 50ul transposition buffer and incubated at 37C for 30 minutes in a thermomixer set to mixing at 1000RPM. The transposition reaction was stopped by

addition of Qiagen's MinElute PCR Purification Binding Buffer, and the transposed DNA was cleaned up according to the manufacturer's protocol. DNA was eluted in 20ul EB buffer, and processed for the 5-cycle Pre-Amplification step, followed by qPCR determination of additional cycles as stated in the protocol. Each sample was further PCR amplified according to the calculated number of additional cycles. The final libraries were cleaned using Qiagen MinElute columns followed by Mag-Bind TotalPure NGS, quantified with the Qubit HS dsDNA kit, and assessed for quality on a TapeStation HS D1000 kit. The libraries were pooled and quantitated by qPCR using KAPA's Illumina Library quantitation kit before PE-150 sequencing on a 10B 300 cycle shared flowcell on the NovaSeq X Plus at the Advanced Genomics Core (University of Michigan).

ATAC-seq sequencing, alignment, filtering, and display file generation

The University of Michigan Epigenomics core used FastQC (https://github.com/s-andrews/FastQC) (v0.11.8) to assess the overall quality of each sequenced sample. They used TrimGalore (https://github.com/FelixKrueger/TrimGalore) (v0.4.5) and cutadapt (21) (v1.15) with the following parameters: –nextera -e 0.1 –stringency 6 –length 20 –nextseq 20. They aligned trimmed reads to with Bowtie2 (22) (v2.3.4.1) with the following parameters: -X 2000 –no-mixed –no-discordant, and defaults multi-seed length of 20bp with 0 mismatches. Duplicate reads were marked with Picard (https://github.com/broadinstitute/picard) (v2.20.2). Alignments to autosomes and sex chromosomes were kept (i.e. mitochondrial reads were removed), duplicates marked by Picard were removed, and alignments below a MAPQ threshold were removed. These filtering steps were performed with samtools (23) (v1.2) and the parameters: -q 10 -F 1024. Reads completely overlapping blacklisted regions (ENCODE Blacklist Regions; https://sites.google.com/site/anshulkundaje/projects/blacklists) were removed with bedtools (24) (v2.28.0). Sample-wise peaks were called with MACS2 (25) (v2.1.2) with flags: -f BAM –nomodel –shift -100 –extsize 200. Peaks over all samples were merged with bedops (26) (v2.4.36) for the

purpose of principal component analysis and unsupervised clustering to assess the similarity of samples. Finally, MultiQC (27) (v1.7) generated a report combining FastQC, trimming, alignment, and duplicate calling over all the samples. For ATAC specific QC metrics we use ataqv (Ataqv) (v1.0.0) (https://github.com/ParkerLab/ataqv).

Differential peak analysis

Differential peak analysis was performed using DiffBind (version 3.6.5). A binding matrix with scores based on read counts for each sample was calculated using dba.count() function. Then the comparisons of interest were modeled using dba.contrast() function. DESeq2 was used as the underlying quantification and differential testing method.

Primary cell T-ALL ATAC-seq

The use of patient samples was approved by the institutional review board at St. Jude Children's Research Hospital and these patient samples were part of a cohort that was previously published and described (28). Written informed consent was provided by all patients and/or their legal guardians. ATAC-seq was performed using the Fast-ATAC (29) protocol on 10,000 cells and utilized identical protocols as previously described (28). Following cell pelleting, transposition reactions (25 mL TD buffer, 2.5 mL TDE1 and 22 mL nuclease free water) were performed at 37 °C using a thermomixer (300 rpm) in the presence of digitonin (0.5 mL 1% digitonin). Following DNA purification (MinElute PCR Purification Kit, cat # 28004), next generation sequencing libraries were amplified using NEBNext 2x PCR master mix (New England Biolabs, cat # M0541L) and indexing primers and sequenced on a NovaSeq 6000 or NovaSeq X+ (Illumina) using 150bp paired-end sequencing. ATAC-seq next-generation sequencing reads were analyzed as previously outlined(28). Reads were trimmed (using TrimGalore v0.6.6) (30), mapped to the hg19 genome (using Bowtie2 v2.2.9) (22), filtered for quality (using the Samtools (23) command "view"

and the options “-q 20 -b”), sorted (Picard v1.141)(31) and mitochondrial reads were removed. ATAC-seq peaks were identified using MACS2 v2.1.1(25) using the “BAMPE” option.

Enhancer motif and conservation analysis

To extract conserved sequences from multiple vertebrate species corresponding to the H-Me NFR (chr6: 135323327-135323580), evolutionarily conserved sub-regions were manually defined based on clusters of predicted evolutionarily conserved elements in hg38 (Phastcons vertebrate 100-way). We then extracted ungapped sequences aligned to the hg38 intervals consisting of the longest mappable region with base matching ratio of 0.1 for each of 47 vertebrate species for which pairwise MULTIZ chain files to hg38 were available. HOMER known motif analysis was then applied to the syntenic interval for each species, and specific motifs and motif families of interest were selected for visualization.

Supplemental references

1. McCarter AM, Della Gatta G, Melnick A, Kim E, Sha C, Wang Q, et al. Combinatorial ETS1-dependent control of oncogenic NOTCH1 enhancers in T-cell leukemia. *Blood Cancer Discovery.* 2020;1(2):178-97.
2. Melnick AF, Mullin C, Lin K, McCarter AC, Liang S, Liu YE, et al. Cdc73 protects Notch-induced T-cell leukemia cells from DNA damage and mitochondrial stress. *Blood.* 2023;142(25):2159-74.
3. Herranz D, Ambesi-Impiombato A, Palomero T, Schnell SA, Belver L, Wendorff AA, et al. A NOTCH1-driven MYC enhancer promotes T cell development, transformation and acute lymphoblastic leukemia. *Nat Med.* 2014;20(10):1130-7.
4. Yashiro-Ohtani Y, Wang H, Zang C, Arnett KL, Bailis W, Ho Y, et al. Long-range enhancer activity determines Myc sensitivity to Notch inhibitors in T cell leukemia. *Proc Natl Acad Sci U S A.* 2014.
5. Polonen P, Di Giacomo D, Seffernick AE, Elsayed A, Kimura S, Benini F, et al. The genomic basis of childhood T-lineage acute lymphoblastic leukaemia. *Nature.* 2024;632(8027):1082-91.
6. Wang Q, Boccalatte F, Xu J, Gambi G, Nadorp B, Akter F, et al. Native stem cell transcriptional circuits define cardinal features of high-risk leukemia. *J Exp Med.* 2025;222(4).
7. Layden HM, Eleuteri NA, Hiebert SW, and Stengel KR. A protocol for rapid degradation of endogenous transcription factors in mammalian cells and identification of direct regulatory targets. *STAR Protoc.* 2021;2(2):100530.
8. Fulco CP, Nasser J, Jones TR, Munson G, Bergman DT, Subramanian V, et al. Activity-by-contact model of enhancer-promoter regulation from thousands of CRISPR perturbations. *Nat Genet.* 2019;51(12):1664-9.
9. Heinz S, Benner C, Spann N, Bertolino E, Lin YC, Laslo P, et al. Simple combinations of lineage-determining transcription factors prime cis-regulatory elements required for macrophage and B cell identities. *Mol Cell.* 2010;38(4):576-89.
10. McKenna A, and Shendure J. FlashFry: a fast and flexible tool for large-scale CRISPR target design. *BMC Biol.* 2018;16(1):74.
11. Sanson KR, Hanna RE, Hegde M, Donovan KF, Strand C, Sullender ME, et al. Optimized libraries for CRISPR-Cas9 genetic screens with multiple modalities. *Nat Commun.* 2018;9(1):5416.
12. Wang T, Lander ES, and Sabatini DM. Single Guide RNA Library Design and Construction. *Cold Spring Harb Protoc.* 2016;2016(3):pdb prot090803.
13. Iyer AR, Gurumurthy A, Kodgule R, Aguilar AR, Saari T, Ramzan A, et al. Selective Enhancer Dependencies in MYC -Intact and MYC -Rearranged Germinal Center B-cell Diffuse Large B-cell Lymphoma. *bioRxiv.* 2023.
14. Li W, Xu H, Xiao T, Cong L, Love MI, Zhang F, et al. MAGeCK enables robust identification of essential genes from genome-scale CRISPR/Cas9 knockout screens. *Genome Biol.* 2014;15(12):554.
15. Pinnell N, Yan R, Cho HJ, Keeley T, Murai MJ, Liu Y, et al. The PIAS-like Coactivator Zmiz1 Is a Direct and Selective Cofactor of Notch1 in T Cell Development and Leukemia. *Immunity.* 2015;43(5):870-83.
16. Unnikrishnan A, Guan YF, Huang Y, Beck D, Thoms JA, Peirs S, et al. A quantitative proteomics approach identifies ETV6 and IKZF1 as new regulators of an ERG-driven transcriptional network. *Nucleic Acids Res.* 2016;44(22):10644-61.
17. Kong AT, Leprevost FV, Avtonomov DM, Mellacheruvu D, and Nesvizhskii AI. MSFragger: ultrafast and comprehensive peptide identification in mass spectrometry-based proteomics. *Nat Methods.* 2017;14(5):513-20.

18. da Veiga Leprevost F, Haynes SE, Avtonomov DM, Chang HY, Shanmugam AK, Mellacheruvu D, et al. Philosopher: a versatile toolkit for shotgun proteomics data analysis. *Nat Methods.* 2020;17(9):869-70.
19. Djomehri SI, Gonzalez ME, da Veiga Leprevost F, Tekula SR, Chang HY, White MJ, et al. Quantitative proteomic landscape of metaplastic breast carcinoma pathological subtypes and their relationship to triple-negative tumors. *Nat Commun.* 2020;11(1):1723.
20. Corces MR, Trevino AE, Hamilton EG, Greenside PG, Sinnott-Armstrong NA, Vesuna S, et al. An improved ATAC-seq protocol reduces background and enables interrogation of frozen tissues. *Nat Methods.* 2017;14(10):959-62.
21. Martin M. Cutadapt removes adapter sequences from high-throughput sequencing reads. *EMBnetjournal.*17(1).
22. Langmead B, and Salzberg SL. Fast gapped-read alignment with Bowtie 2. *Nat Methods.* 2012;9(4):357-9.
23. Li H, Handsaker B, Wysoker A, Fennell T, Ruan J, Homer N, et al. The Sequence Alignment/Map format and SAMtools. *Bioinformatics.* 2009;25(16):2078-9.
24. Quinlan AR, and Hall IM. BEDTools: a flexible suite of utilities for comparing genomic features. *Bioinformatics.* 2010;26(6):841-2.
25. Zhang Y, Liu T, Meyer CA, Eeckhoute J, Johnson DS, Bernstein BE, et al. Model-based analysis of ChIP-Seq (MACS). *Genome Biol.* 2008;9(9):R137.
26. Neph S, Kuehn MS, Reynolds AP, Haugen E, Thurman RE, Johnson AK, et al. BEDOPS: high-performance genomic feature operations. *Bioinformatics.* 2012;28(14):1919-20.
27. Ewels P, Magnusson M, Lundin S, and Kaller M. MultiQC: summarize analysis results for multiple tools and samples in a single report. *Bioinformatics.* 2016;32(19):3047-8.
28. Barnett KR, Mobley RJ, Diedrich JD, Bergeron BP, Bhattarai KR, Monovich AC, et al. Epigenomic mapping reveals distinct B cell acute lymphoblastic leukemia chromatin architectures and regulators. *Cell Genom.* 2023;3(12):100442.
29. Corces MR, Buenrostro JD, Wu B, Greenside PG, Chan SM, Koenig JL, et al. Lineage-specific and single-cell chromatin accessibility charts human hematopoiesis and leukemia evolution. *Nat Genet.* 2016;48(10):1193-203.
30. Krueger F. Trim galore. A wrapper tool around Cutadapt and FastQC to consistently apply quality and adapter trimming to FastQ files 516. 2015.
31. Institute B. Picard Toolkit. Available online at: http://broadinstitute.github.io/picard. http://broadinstitute.github.io/picard. 2019.
