# Supplementary material for: The hematopoietic stem cell MYB enhancer is essential for and recurrently amplified during T cell leukemogenesis
Source: J Clin Invest. 2025 Oct 23;136(1):e187998. doi: 10.1172/JCI187998 (PMC12721900; doi:10.1172/JCI187998)

Full unedited gel for Figure 8A

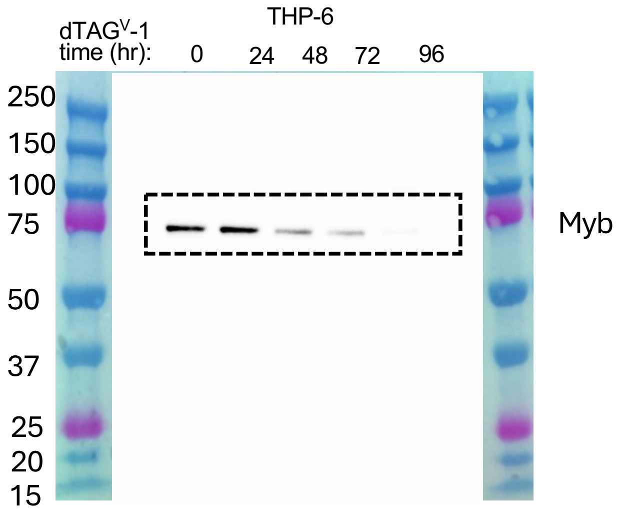

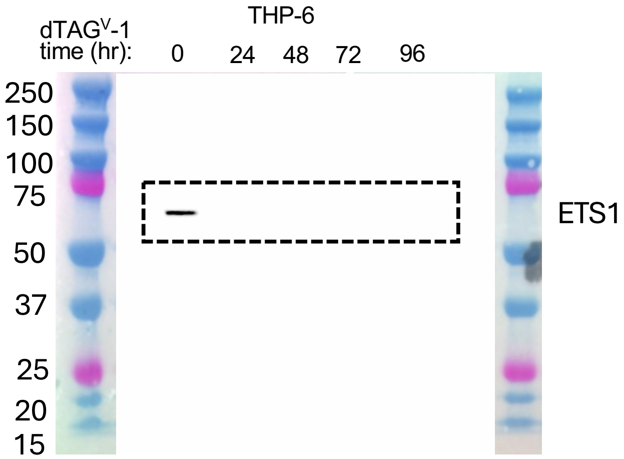

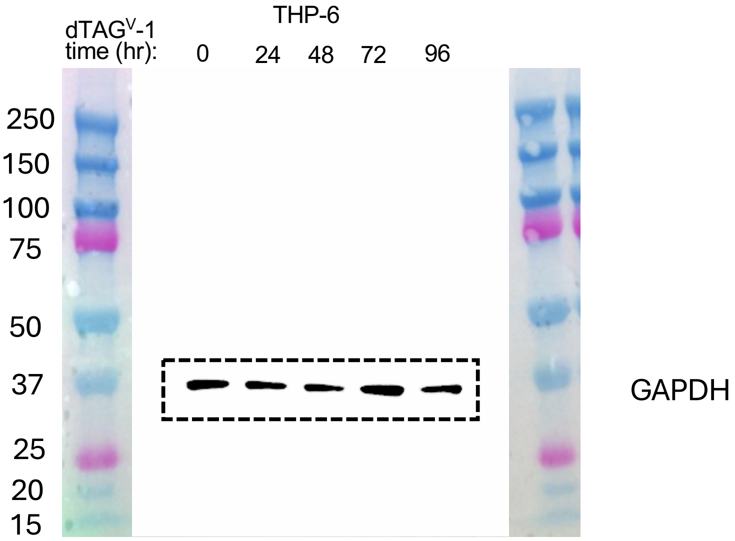

Full unedited gel for Figure 8C

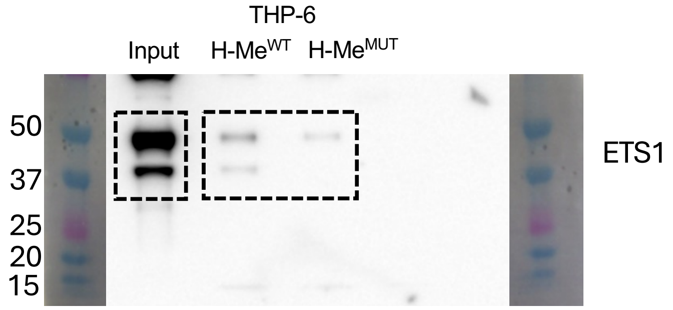

Blot Cut at 75kDa Marker

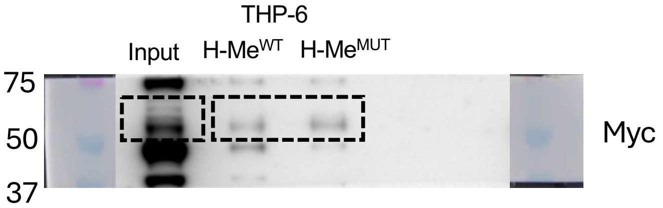

Blot Cut at 75kDa and 37kDa Marker

Full unedited gel for Figure 8K

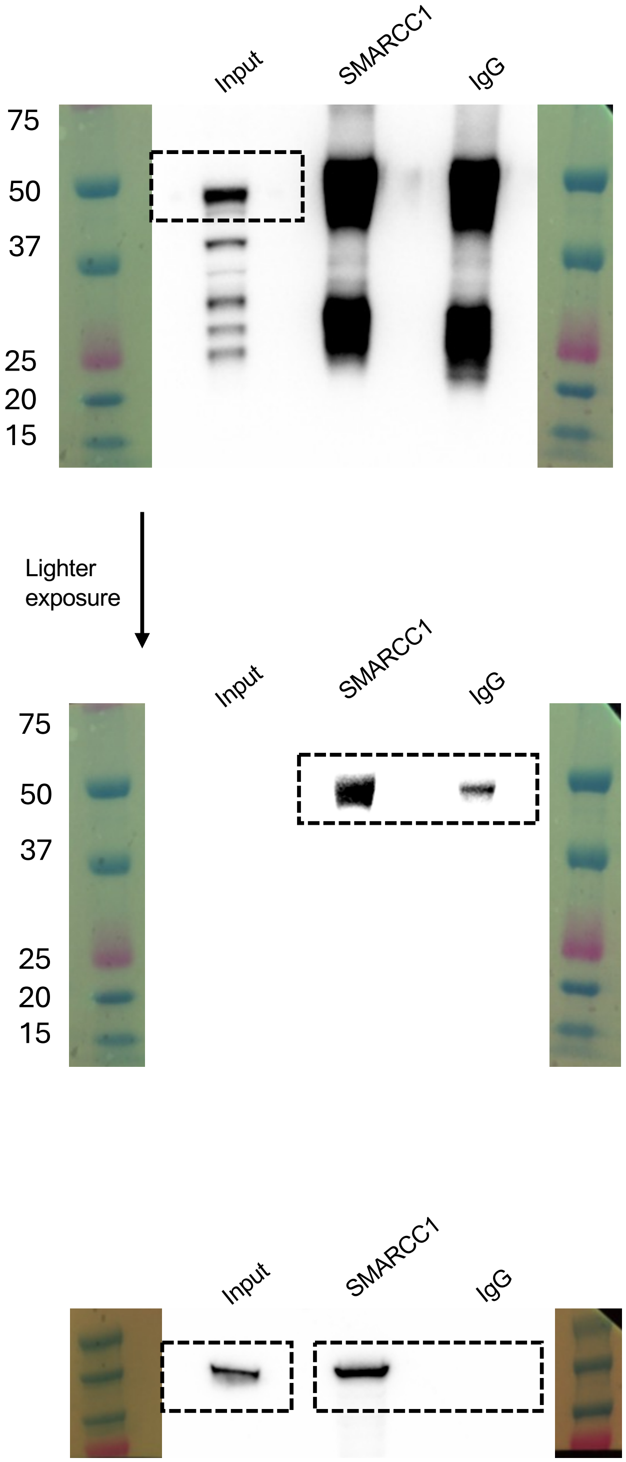

ETS1
Blot Cut at 75kDa

ETS1
Blot Cut at 75kDa

SMARCC1
Blot Cut at 75kDa

Full unedited gel for Figure 8J

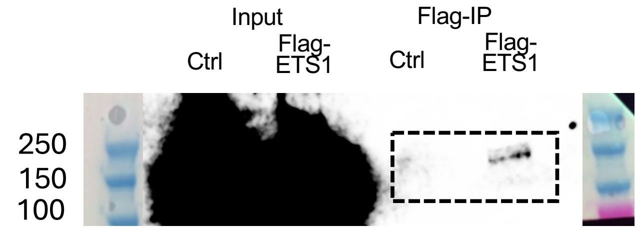

SMARCC1
Blot Cut at 75kDa

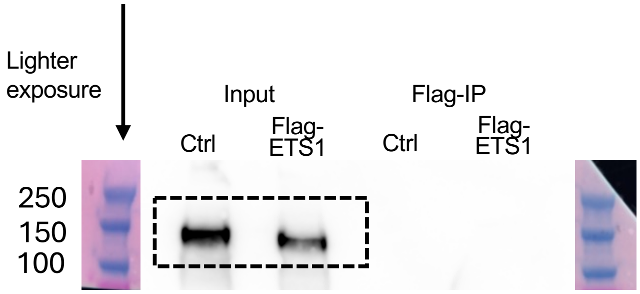

SMARCC1
Blot Cut at 75kDa

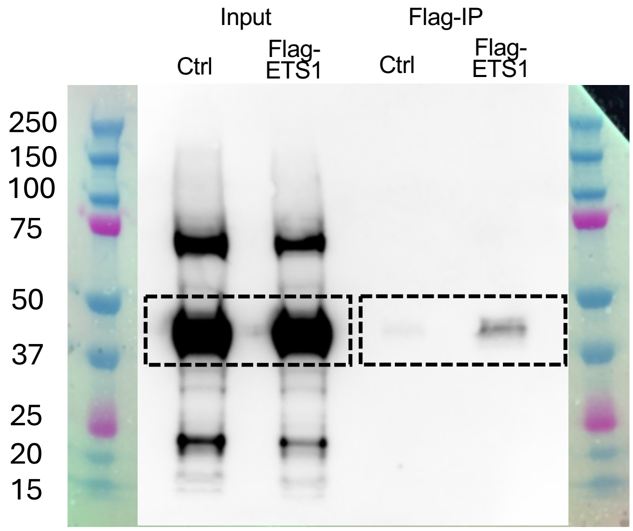

SMARCB1

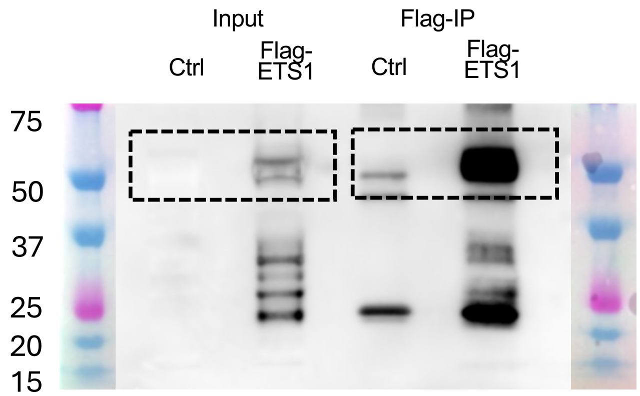

FLAG
Blot Cut at 75kDa

Full unedited gel for Figure 8O and S20A

Blots were split to avoid giving impression that CEM cells and THP-6 are not related.

Figure S20A Figure 8O

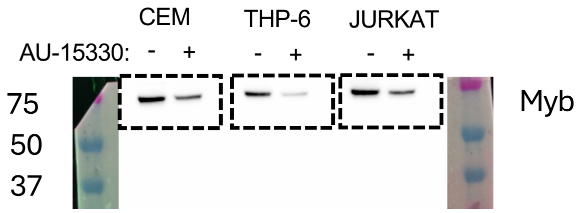

Blot Cut at 75kDa and 25kDa

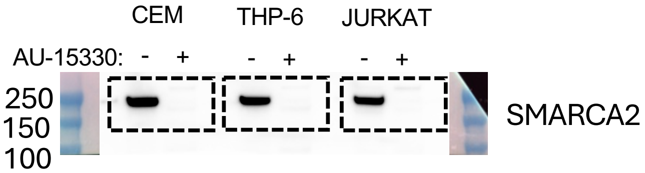

Blot Cut at 75kDa

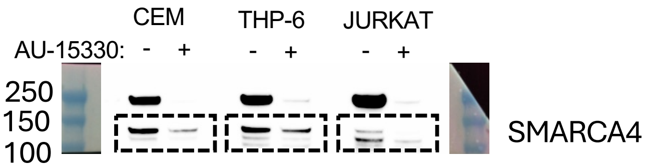

Blot Cut at 75kDa

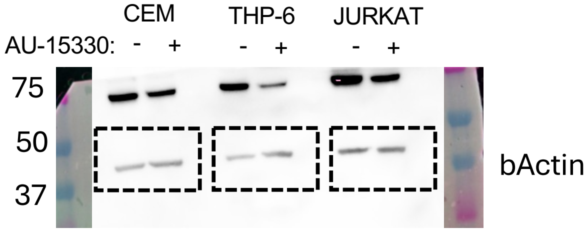

Blot Cut at 75kDa and 25kDa

Full unedited gel for Figure S13J

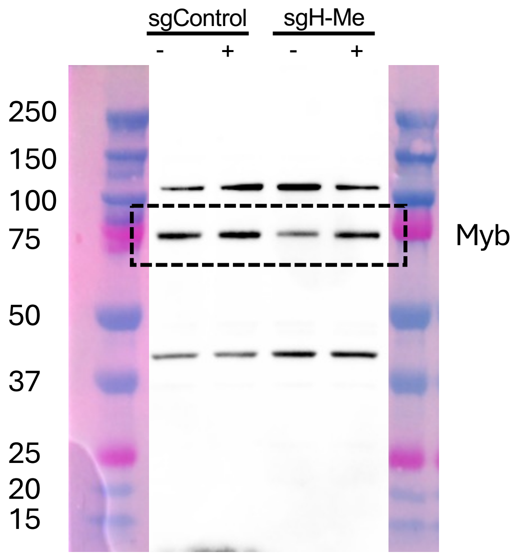

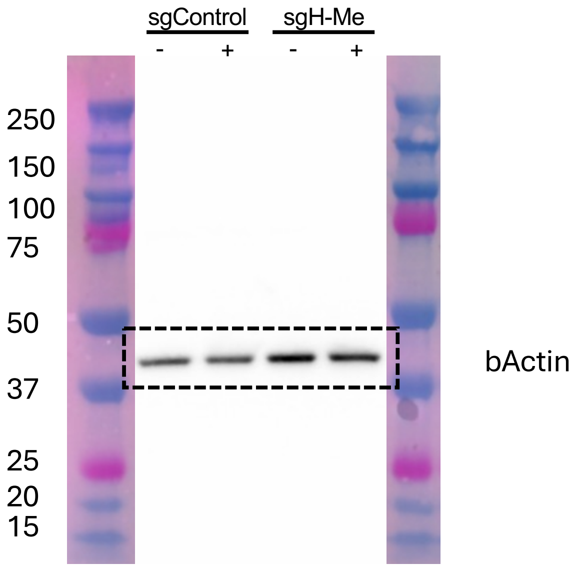

Full unedited gel for Figure S14A

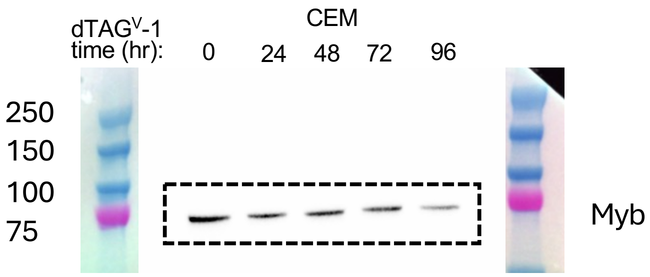

Blot Cut at 50kDa Marker

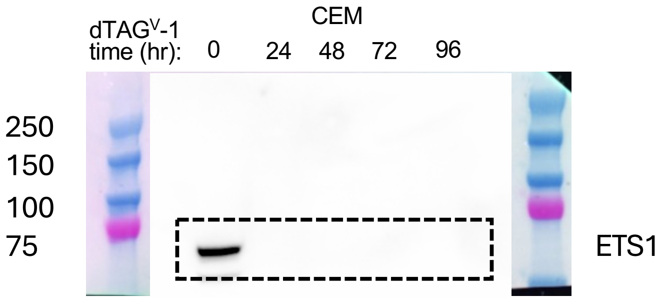

Blot Cut at 50kDa Marker

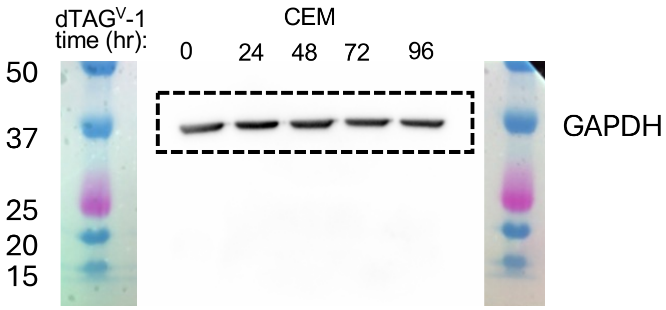

Blot Cut at 50kDa Marker

Full unedited gel for Figure S16B

Fig. S16B

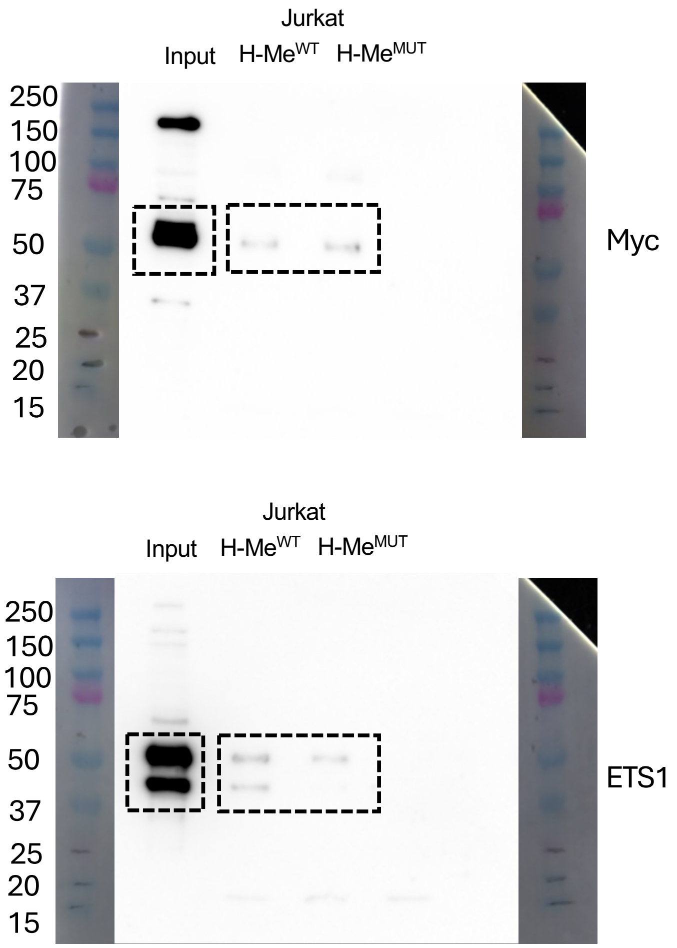

Full unedited gel for Figure S16D

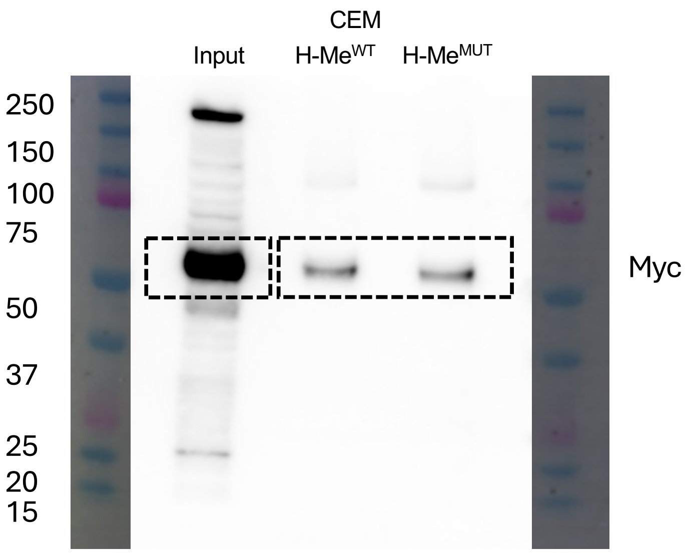

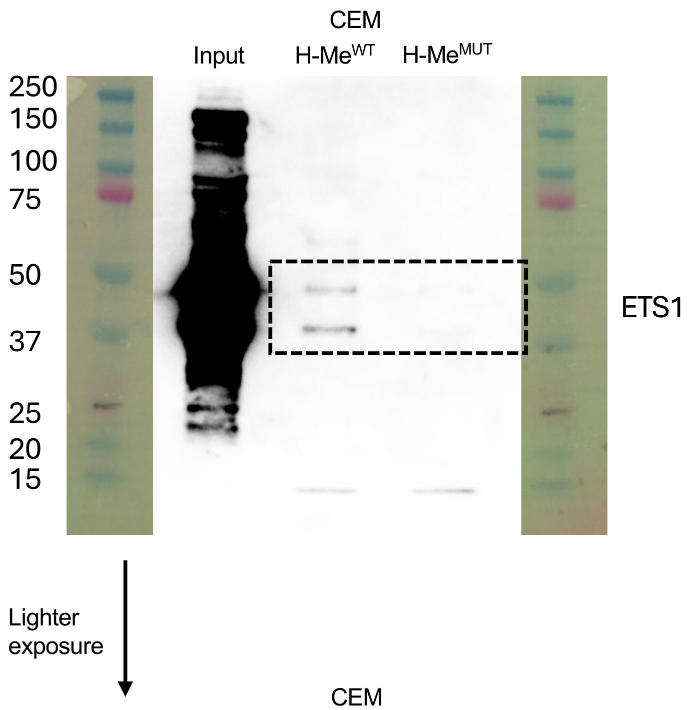

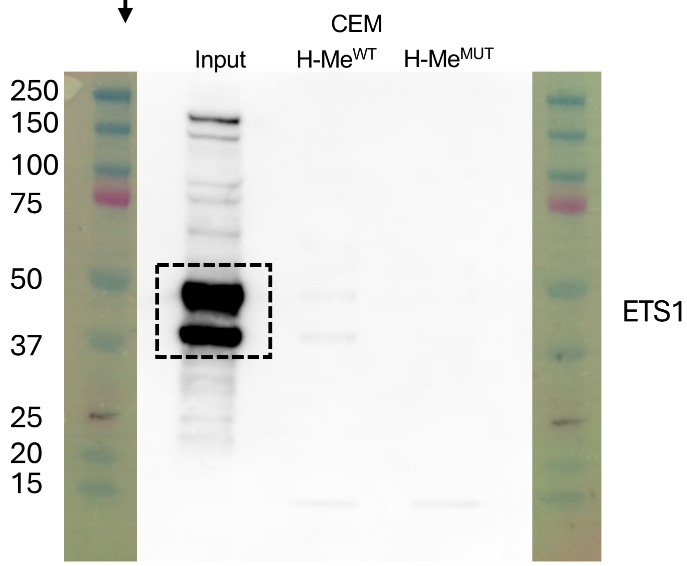

Full unedited gel for Figure S20B

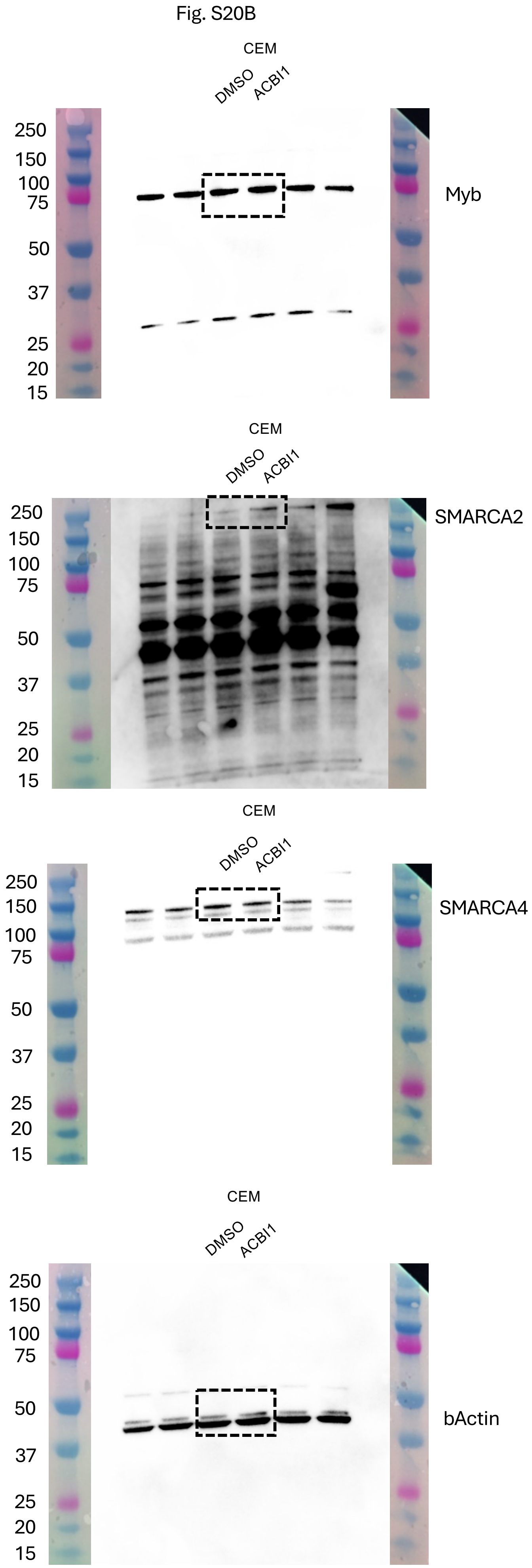

Full unedited gel for Figure S20B (continued)

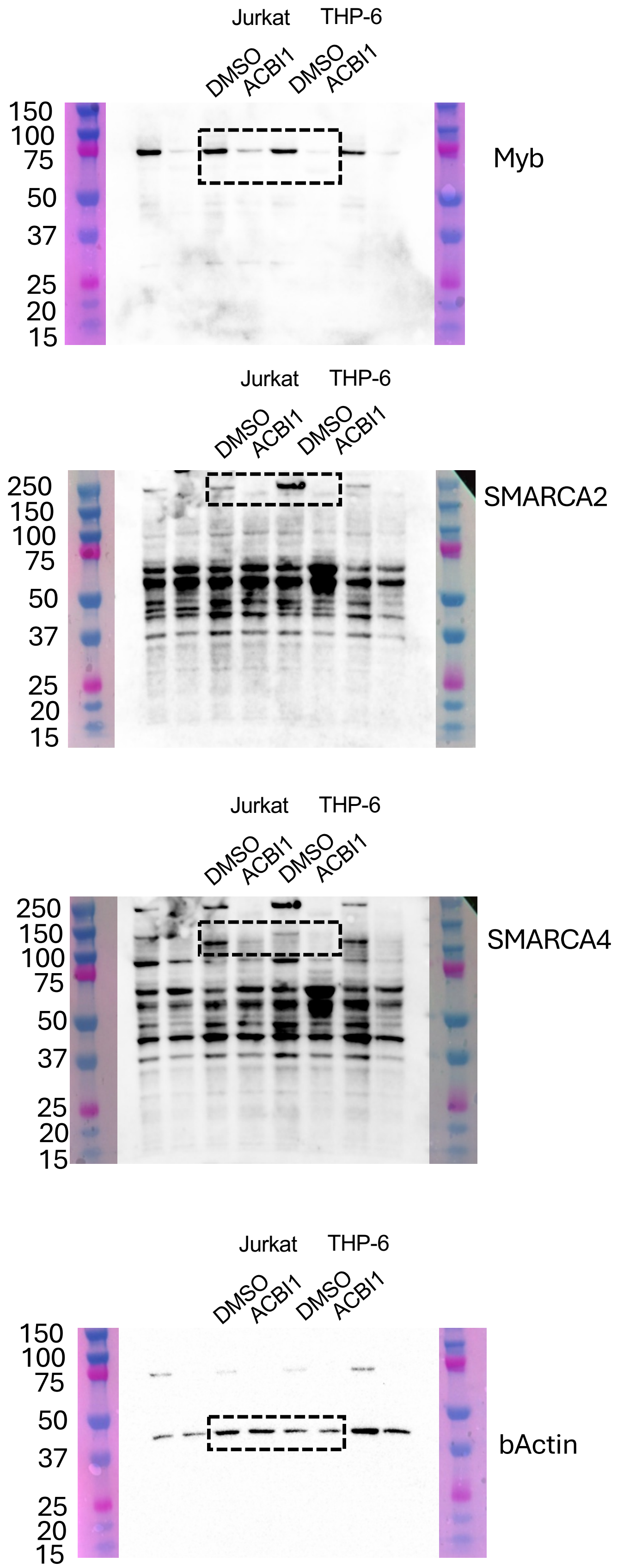

Supplement: Unedited blot and gel images [file jci-136-187998-s156.pdf]
